# Ray Meta: scalable *de novo *metagenome assembly and profiling

**DOI:** 10.1186/gb-2012-13-12-r122

**Published:** 2012-12-22

**Authors:** Sébastien Boisvert, Frédéric Raymond, Élénie Godzaridis, François Laviolette, Jacques Corbeil

**Affiliations:** 1 Infectious Diseases Research Center, CHUQ Research Center, 2705, boul. Laurier, Québec (Québec), G1V 4G2, Canada; 2 Faculty of Medicine, Laval University, 1050, av. de la Médecine, Québec (Québec), G1V 0A6, Canada; 3 Department of Computer Science and Software Engineering, Faculty of Science and Engineering, Laval University, 1065, av. de la Médecine, Québec (Québec), G1V 0A6, Canada; 4 Department of Molecular Medicine, Faculty of Medicine, Laval University, 1050, av. de la Médecine, Québec (Québec), G1V 0A6, Canada

**Keywords:** metagenomics, message passing, scalability, *de novo *assembly, profiling, next-generation sequencing, parallel, distributed

## Abstract

Voluminous parallel sequencing datasets, especially metagenomic experiments, require distributed computing for *de novo *assembly and taxonomic profiling. Ray Meta is a massively distributed metagenome assembler that is coupled with Ray Communities, which profiles microbiomes based on uniquely-colored k-mers. It can accurately assemble and profile a three billion read metagenomic experiment representing 1,000 bacterial genomes of uneven proportions in 15 hours with 1,024 processor cores, using only 1.5 GB per core. The software will facilitate the processing of large and complex datasets, and will help in generating biological insights for specific environments. Ray Meta is open source and available at http://denovoassembler.sf.net.

## Background

While voluminous datasets from high-throughput sequencing experiments have allowed new biological questions to emerge [1,2], the technology's speed and scalability are not yet matched by available analysis techniques and the gap between them has been steadily growing [3,4]. The de Bruijn graph is a structure for storing DNA words - or k-mers - that occur in sequence datasets [5,6]. Recent work showed that adding colors to a de Bruijn graph can allow variants to be called even in the absence of a complete genome reference [7].

The field of metagenomics is concerned with the analysis of communities by sampling the DNA of all species in a given microbial community. The assembly of metagenomes poses greater and more complex challenges than single-genome assembly as the relative abundances of the species in a microbiome are not uniform [8]. A compounding factor is the genetic diversity represented by polymorphisms and homologies between strains, which increases the difficulty of the problem for assemblers [8]. Moreover, the underlying diversity of the sample increases its complexity and adds to the difficulties of assembly. Last but not least, DNA repeats can produce misassemblies [9] in the absence of fine-tuned, accurate computational tools [10].

The microbial diversity in microbiomes contains the promise of finding new genes with novel and interesting biological functions [11]. While the throughput in metagenomics is increasing fast, bottlenecks in the analyses are becoming more apparent [12], indicating that only equally parallel - and perhaps highly distributed - analysis systems can help bridge the scalability gap. Parallel sequencing requires parallel processing for bioprospecting and for making sense of otherwise largely unknown sequences.

Environmental microbiomes have been the subject of several large-scale investigations. Viral genome assemblies have been obtained from samples taken from hot springs [13]. Metabolic profiling of microbial communities from Antarctica [14] and the Arctic [15] provided novel insights into the ecology of these communities. Furthermore, a new Archaea lineage was discovered in a hypersaline environment by means of metagenomic assembly [16]. The metabolic capabilities of terrestrial and marine microbial communities have been compared [17]. The structure of communities in the environment has been reconstructed [18]. All these studies show that environmental microbiomes are reservoirs of genetic novelty [19], which bioprospecting aims at discovering.

Through metagenomic analysis, the interplay between host and commensal microbial metabolic activity can be studied, promising to shed light on its role in maintaining human health. Furthermore, precisely profiling the human microbial and viral flora at different taxonomic levels as well as functional profiling may hint at improved new therapeutic options [20]. To that end, the human distal gut microbiome of two healthy adults was analyzed by DNA sequencing [21], and subsequently the human gut microbiome of 124 European individuals was analyzed by DNA sequencing from fecal samples by the MetaHIT consortium [22]. Another study proposed that there are three stable, location-independent, gut microbiome enterotypes [23]. Finally, the structure, function and diversity of the healthy human microbiome were investigated by the Human Microbiome Project Consortium [24].

With 16S rRNA gene sequencing, species representation can be extracted by taxonomic profiling [25]. However, using more than one marker gene produces better taxonomic profiles [26,27]. Furthermore, a taxonomy based on phylogenetic analyses helps in the process of taxonomic profiling [28]. While taxonomic profiles are informative, functional profiling is also required to understand the biology of a system. To that end, gene ontology [29] can assign normalized functions to data.

Although not designed for metagenomes, distributed software for single genomes, such as ABySS [30] and Ray [31], illustrate how leveraging high-performance and parallel computing could greatly speed up the analysis of the large amount of data generated by metagenome projects. Notably, sophisticated parallel tools are easily deployed on cloud computing infrastructures [32] or on national computing infrastructures through their use of a cross-platform, scalable method called the message-passing interface.

Taxonomic profiling methods utilize alignments [26,27,33-36]or hidden Markov models [37] or both[38]. Few methods are available for metagenome *de novo *assembly (MetaVelvet [39], Meta-IDBA [40] and Genovo [41]), none couples taxonomic and ontology profiling with *de novo *assembly, and none is distributed to provide scalability. Furthermore, none of the existing methods for *de novo *metagenome assembly distributes memory utilization over more than one compute machine. This additional difficulty plagues current metagenome assembly approaches.

The field of metagenomic urgently needs distributed and scalable processing methods to tackle efficiently the size of samples and the assembly and profiling challenges that this poses. Herein we show that Ray Meta, a distributed processing application, is suited for metagenomics. We present results obtained by *de novo *metagenome assembly with coupled profiling. With Ray Meta, we show that the method scales for two metagenomes simulated to incorporate sequencing errors: a 100-genome metagenome assembled from 400 × 10^6 ^101-nucleotide reads and a 1,000-genome metagenome assembled from 3 × 10^9 ^100-nucleotide reads. Ray Communities utilizes bacterial genomes to color the assembled de Bruijn graph. The Greengenes taxonomy [28] was utilized to obtain the profiles from colored k-mers. Other taxonomies, such as the NCBI taxonomy, can be substituted readily. We also present results obtained by *de novo *metagenome assembly and taxonomic and functional profiling of 124 gut microbiomes. We compared Ray Meta to MetaVelvet and validated Ray Communities with MetaPhlAn taxonomic profiles.

## Results

### Scalability

In order to assess the scalability of Ray Meta, we simulated two large datasets. Although a simulation does not capture all genetic variations (and associated complexity) occurring in natural microbial populations, it is a way to validate the correctness of assemblies produced by Ray Meta and the abundances predicted by Ray Communities. The first dataset contained 400 × 10^6 ^reads, with 1% as human contamination. The remaining reads were distributed across 100 bacterial genomes selected randomly from GenBank. The read length was 101 nucleotides, the substitution error rate was 0.25% and reads were paired. Finally, the proportion of bacterial genomes followed a power law (with exponent -0.5) to mimic what is found in nature (see the section on Materials and methods). The number of reads for this 100-genome metagenome roughly corresponds to the number of reads generated by one lane of an Illumina HiSeq 2000 flow cell (Illumina, Inc.). Table S1 in Additional file 1 lists the number of reads for each bacterial genome and for the human genome. This dataset was assembled by Ray Meta using 128 processor cores in 13 hours, 26 minutes, with an average memory usage of 2 GB per core. The resulting assembly contained 22,162 contigs with at least 100 nucleotides and had an N50 of 152,891. The sum of contig lengths was 345,945,478 nucleotides. This is 93% of the sum of bacterial genome lengths, which was 371,623,377 nucleotides. Therefore, on average there were 3,459,454 assembled nucleotides and 221 contigs per bacterial genome, assuming that the bacterial genomes were roughly of the same size and same complexity and that the coverage depth was not sufficient to assemble incorporated human contamination. Using the known reference sequences, we validated the assembly using MUMmer to assess the quality. There were 11,220 contigs with at least 500 nucleotides. Among these, 152 had misassemblies (1.35%). Any contig that did not align as one single maximum unique match with a breadth of coverage of at least 98.0% was marked as misassembled. The number of mismatches was 1,108 while the number of insertions or deletions was 597.

To further investigate the scalability of our approach for *de novo *metagenome assembly, we simulated a second metagenome. This one contained 1,000 bacterial genomes randomly selected from GenBank as well as 1% of human sequence contamination. The proportion of the 1,000 bacterial genomes was distributed according to a power law (with exponent -0.3) and the number of reads was 3 × 10^9 ^(Table S2 in Additional file 1). This number of reads is currently generated by one Illumina HiSeq 2000 flow cell (Illumina, Inc.). This second dataset, which is larger, was assembled *de novo *by Ray Meta in 15 hours, 46 minutes using 1,024 processor cores with an average memory usage of 1.5 GB per core. It contained 974,249 contigs with at least 100 nucleotides; N50 was 76,095 and the sum of the contig lengths was 2,894,058,833, or 80% of the sum of bacterial genome lengths (3,578,300,288 nucleotides). Assuming a uniform distribution of assembled bases and contigs and that human sequence coverage depth was not sufficient for its *de novo *assembly, there were, on average, 974 contigs and 2,894,058 nucleotides per bacterial genome. To validate whether or not the produced contigs were of good quality, we compared them to the known references. There were 196,809 contigs with at least 500 nucleotides. Of these, 2,638 were misassembled (1.34%) according to a very stringent test. There were 59,856 mismatches and 13,122 insertions or deletions.

**Figure 1:**
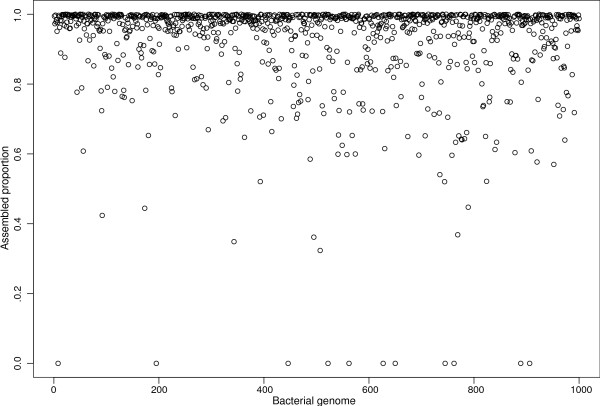
**Assembled proportions of bacterial genomes for a simulated metagenome with sequencing errors**. 3 × 10^9 ^100-nucleotide reads were simulated with sequencing errors (0.25%) from a simulated metagenome containing 1,000 bacterial genomes with proportions following a power law. Having 1,000 genomes with power law proportions makes it impossible to classify sequences with their coverage. This large metagenomic dataset was assembled using distributed de Bruijn graphs and profiled with colored de Bruijn graphs. Highly similar, but different genomes, are likely to be hard to assemble. This figure shows the proportion of each genome that was assembled *de novo *within the metagenome. Of the bacterial genomes, 88.2% were assembled with a breadth of coverage of at least 80.0%.

Next, we sought to quantify the breadth of assembly for the bacterial genomes in the 1,000-genome dataset. In other words, the assembled percentage was calculated for each genome present in the 1,000-genome metagenome. Many of these bacterial genomes had a breadth of coverage (in the *de novo *assembly) greater than 95% (Figure 1).

### Estimating bacterial proportions

Another problem that can be solved with de Bruijn graphs is estimating the genome nucleotide proportion within a metagenome. Using Ray Communities, the 100-genome and 1,000-genome datasets *de novo *assembled de Bruijn graphs were colored using all sequenced bacterial genomes (Table S4 in Additional file 1) in order to identify contigs and to estimate bacterial proportions in the datasets. Ray Communities estimates proportions by demultiplexing k-mer coverage depth in the distributed de Bruijn graph (see the section on Demultiplexing signals from similar bacterial strains in Materials and methods). Because coloring occurs after *de novo *assembly has completed, the reference sequences are not needed for assembling metagenomes.

**Figure 2:**
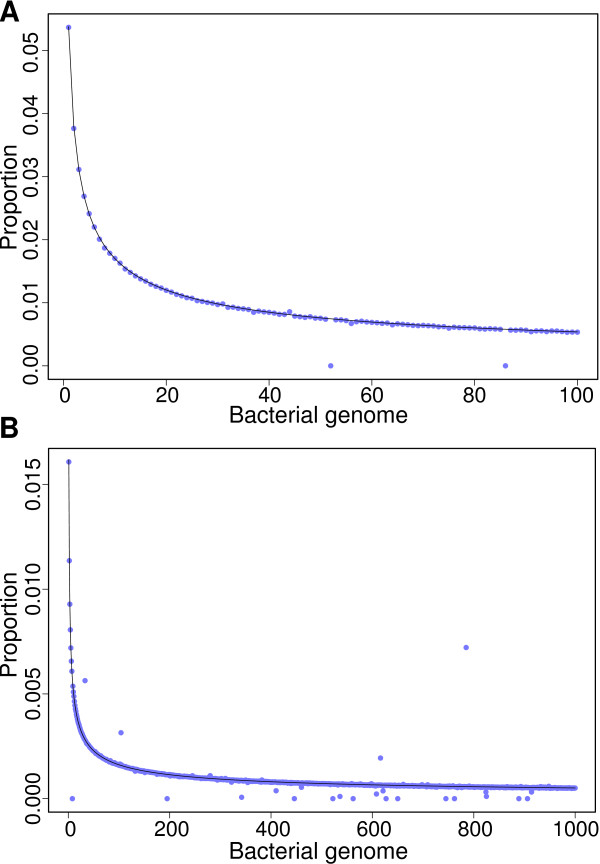
**Estimated bacterial genome proportions**. For the two simulated metagenomes (100 and 1,000 bacterial genomes, respectively), colored de Bruijn graphs were utilized to estimate the nucleotide proportion of each bacterial genome in its containing metagenome. Genome proportions in metagenomes followed a power law. Black lines show the expected nucleotide proportion for bacterial genomes while blue points represent proportions measured by colored de Bruijn graphs. **(A) **For the 100-genome metagenome, only two bacterial genomes were not correctly measured (2.0%), namely *Methanococcus maripaludis *X1 and *Serratia *AS9. *Methanococcus maripaludis *X1 was not detected because it was duplicated in the dataset as *Methanococcus maripaludis *XI, thus providing zero uniquely colored k-mers. *Serratia *AS9 was not detected because it shares almost all its k-mers with *Serratia *AS12. **(B) **For the 1,000-genome metagenome, 4 bacterial genomes were overestimated (0.4%) while 20 were underestimated (2.0%). These errors were due to highly similar bacterial genomes, hence they did not provide uniquely colored k-mers. This problem can be alleviated either by using a curated set of reference genomes or by using a taxonomy. The remaining 976 bacterial genomes had a measured proportion near the expected value.

For the 100-genome dataset, only two bacterial genome proportions were not estimated correctly. The first was due to a duplicate in GenBank and the second to two almost identical genomes (Figure 2A). When two identical genomes are provided as a basis to color the de Bruijn graph, no k-mer is uniquely colored for any of these two genomes, and identifying k-mers cannot be found through demultiplexing. This can be solved by using a taxonomy, which allows reference genomes to be similar or identical.

In the 1,000-genome dataset, four bacterial genome proportions were overestimated and 20 were underestimated (Figure 2B). In both the 100-genome and 1,000-genome datasets, the proportion of bacterial genomes with incorrect estimates was 2.0%. In both of these, the incorrect estimates were caused by either duplicated genomes, identical genomes or highly similar genomes. The use of a taxonomy alleviates this problem.

The results with the 100-genome and 1,000-genome datasets show that our method can recover bacterial genome proportions when the genome sequences are known. In real microbiome systems, there is a sizable proportion of unknown bacterial species. For this reason, it is important to devise a system that can also accommodate unknown species by using a taxonomy, which allows the classification to occur at higher levels - such as phylum or genus instead of species.

### Metagenome de novo assembly of real datasets

Here, we present results for 124 fecal samples from a previous study [22]. From the 124 samples, 85 were from Denmark (all annotated as being healthy) and 39 were from Spain (14 were healthy, 21 had ulcerative colitis and 4 had Crohn's disease). Each metagenome was assembled independently (Table S3 in Additional file 1) and the resulting distributed de Bruijn graphs were colored to obtain taxonomic and gene ontology profiles (see Materials and methods and Table S4 in Additional file 1).

These samples contained paired 75-nucleotide and/or 44-nucleotide reads obtained with Illumina Genome Analyzer sequencers. In about 5 hours, 122 samples were assembled (and profiled) using 32 processor cores and the two remaining samples, namely MH0012 and MH0014, were assembled (and profiled) with 48 and 40 processor cores, respectively (Table S3 in Additional file 1). These runtime figures include *de novo *assembly, graph coloring, signal demultiplexing and taxonomic and gene ontology profiling, which are all tightly coupled in the process. In the next section, taxonomic profiles are presented for these 124 gut microbiome samples.

### Taxonomic profiling

In metagenomic projects, the bacterial genomes that occur in the sample may be unknown at the species level. However, it is possible to profile these samples using a taxonomy. The key concept is to classify colored k-mers in a taxonomy tree: a k-mer is moved to a higher taxon as long as many taxons have the k-mer so it can be classified as the nearest common ancestor of the taxons. For example if a k-mer is not classified at the species level, it can be classified at the genus level and so on. Furthermore, taxonomy profiling does not suffer from similarity issues as seen for proportions present in samples because k-mers can be classified in higher taxons when necessary.

**Figure 3:**
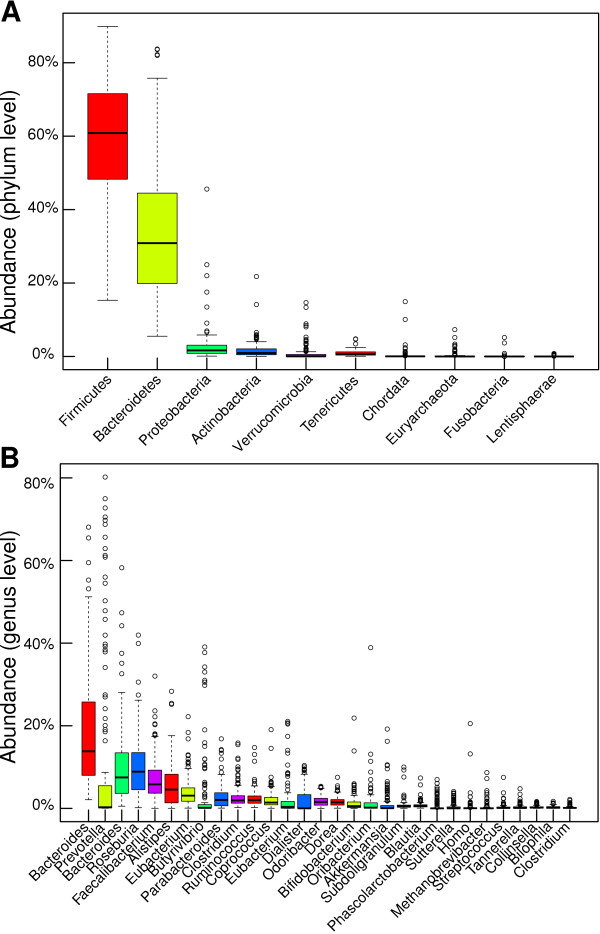
**Fast and efficient taxonomic profiling with distributed colored de Bruijn graphs**. From a previous study, 124 metagenomic samples containing short paired reads were assembled *de novo *and profiled for taxons. The graph coloring occurred once the de Bruijn graph was assembled *de novo*. **(A) **The taxonomic profiles are shown for the phylum level. The two most abundant phyla were Firmicutes and Bacteroidetes. This is in agreement with the literature [22]. The abundance of human sequences was also measured. The phylum Chordata had two outlier samples. This indicates that two of the samples had more human sequences than the average, which may bias results. **(B) **At the genus level, the most abundant taxon was *Bacteroides*. This taxon occurred more than once because it was present at different locations within the Greengenes taxonomic tree. Also abundant is the genus *Prevotella*. Furthermore, the later had numerous samples with higher counts, which may help in non-parametric clustering. Two samples had higher abundance of human sequences, as indicated by the abundance of the genus *Homo*.

Accordingly, k-mers shared by several bacterial species cannot be assigned to one of them accurately. For this reason, the Greengenes taxonomy [28] (version 2011_11) was utilized to classify each colored k-mer in a single taxon with its taxonomic rank being one of the following: kingdom, phylum, class, order, family, genus or species. For each sample, abundances were computed at each taxonomic rank. At the moment, the most recent and accurate taxonomy for profiling taxons in a metagenome is Greengenes [28]. We profiled taxons in the 124 gut microbiome samples using this taxonomy. We also incorporated the human genome into this taxonomy to profile the human abundance in the process. At the phylum level, the two most abundant taxons were Firmicutes and Bacteroidetes (Figure 3A). The profile of the phylum Chordata indicated that two samples contained significantly more human sequences than the average (Figure 3A). The most abundant genera in the 124 samples were *Bacteroides *and *Prevotella *(Figure 3B). The taxon *Bacteroides *is reported more than once because several taxons had this name with a different ancestry in the Greengenes taxonomy. The genera *Prevotella *and *Butyrivibrio *had numerous samples with higher counts, indicating that the data are bi-modal (Figure 3B). The genus *Homo *had two samples with significantly more abundance (Figure 3B).

### Grouping abundance profiles

**Figure 4:**
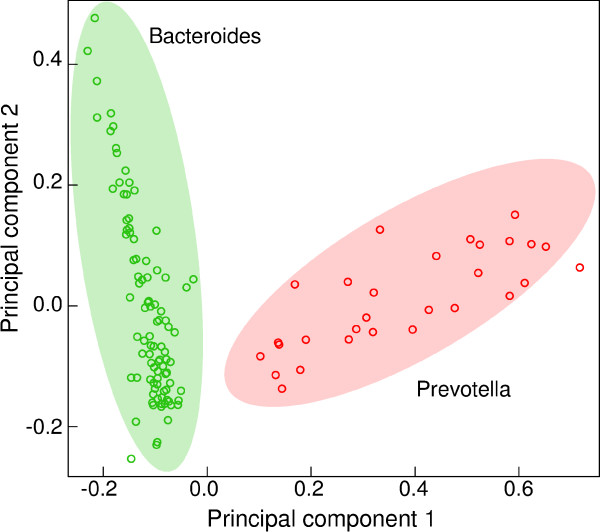
**Principal component analysis shows two clusters**. Principal component analysis (see Materials and methods) with abundances at the genus level yielded two distinct clusters. Abundances were obtained with colored de Bruijn graphs. One was enriched in the genus *Bacteroides *while the other was enriched in the genus *Prevotella*. Principal component 1 was linearly correlated with the genus *Prevotella *while principal component 2 was linearly correlated with the genus *Bacteroides*. This analysis suggests that there is a continuum between the two abundant genera *Bacteroides *and *Prevotella*. This interpretation differs from the original publication in which three human gut enterotypes were reported [23]. More recently, it has been proposed that there are only two enterotypes and individuals are distributed in a continuum between the two [42].

It has been proposed that the composition of the human gut microbiome of an individual can be classified as one of three enterotypes [23]. We profiled genera for each of the 124 gut microbiome samples to reproduce these three enterotypes. The 124 samples (85 from Denmark and 39 from Spain) were analyzed using the two most important principal components (Figure 4; see Materials and methods). Two clear clusters are visible, one enriched for the genus *Bacteroides *and one for the genus *Prevotella*. A continuum between two enterotypes has also been reported recently [42].

### Profiling of ontology terms

**Figure 5:**
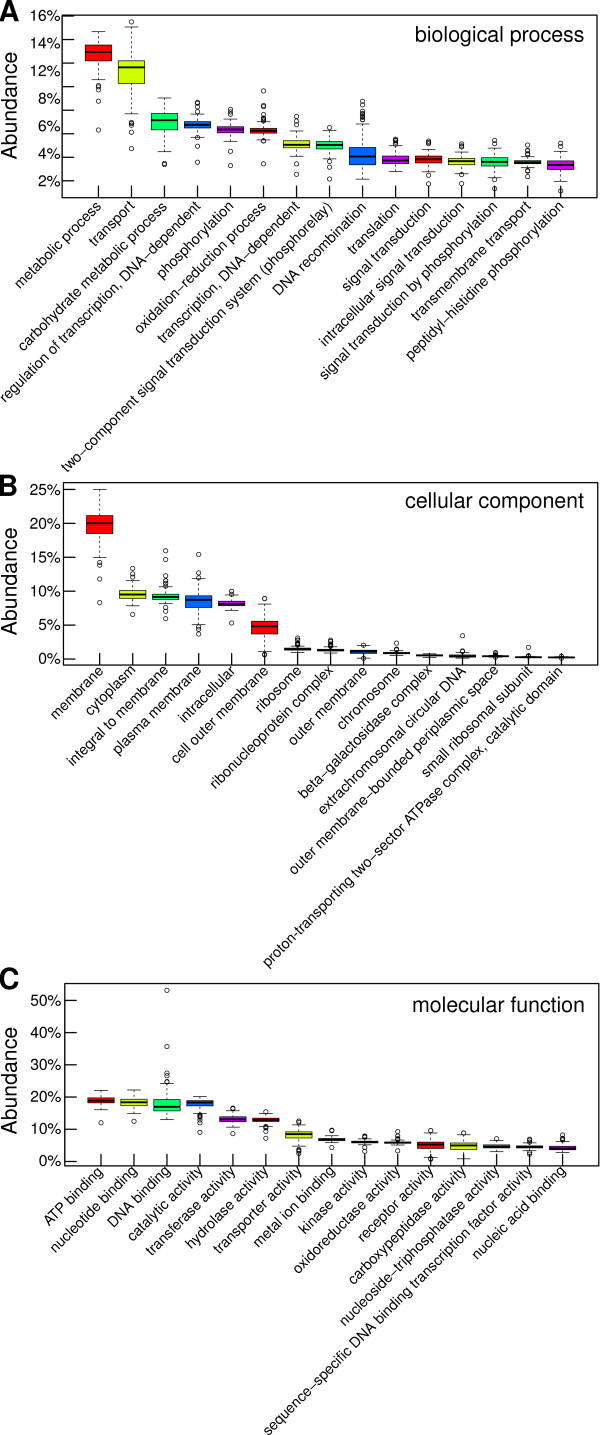
**Ontology profiling with colored de Bruijn graphs**. Gene ontology profiles were obtained by coloring of the graph resulting from *de novo *assembly. Gene ontology has three domains: biological process, cellular component and molecular function. For each domain, only the 15 most abundant terms are displayed. **(A) **Ontology terms in the biological process domain were profiled. Some of these have several outlier samples, namely oxidation-reduction process and DNA recombination. **(B) **Ontology profiling for cellular component terms is shown. The most abundant is the membrane term. **(C) **The profile for molecular function terms is shown. Binding functions are the most abundant with ATP binding, nucleotide binding and DNA binding in the top three. Next is catalytic activity, which is a general term. More specific catalytic activities are listed.

Gene ontology is a hierarchical classification of normalized terms in three independent domains: biological process, cellular component and molecular function. Some biological datasets are annotated with gene ontology. Here, we used gene ontology to profile the 124 metagenome samples based on a distributed colored de Bruijn graph (see Materials and methods). First, abundances for biological process terms were obtained (Figure 5A). The two most abundant terms were metabolic process and transport. The terms oxidation-reduction process and DNA recombination had numerous sample outliers, which indicates that these samples had different biological complexity for these terms (Figure 5A). Next, we sought to profile cellular component terms in the samples. The most abundant term was membrane, followed by cytoplasm, integral to membrane and plasma membrane. This redundancy is due to the hierarchical structure of gene ontology (Figure 5B). Finally, we measured the abundance for molecular function terms. The most abundant was ATP binding, which had no outliers. The term DNA binding was also abundant. However, the latter had outlier samples (Figure 5C).

### Comparison of assemblies

Three samples from the MetaHIT Consortium [22] - MH0006 (ERS006497), MH0012 (ERS006494) and MH0047 (ERS006592) - and three samples from the Human Microbiome Project Consortium [24] - SRS011098, SRS017227 and SRS018661 - were assembled with MetaVelvet [39] and Ray Meta to draw a comparison. Assembly metrics are displayed in Table 1. The average length is higher for MetaVelvet for samples ERS006494 and ERS006592. For the other samples, the average length is higher for Ray Meta. The N50 length is higher for Ray Meta for all samples. For all samples but ERS006497, the total length is higher for Ray Meta. Although we assembled the 124 samples from [22] and 313 samples (out of 764) from the Human Microbiome Project [24] with Ray Meta on supercomputers composed of nodes with little memory (24 GB), we only assembled a few samples with MetaVelvet because a single MetaVelvet assembly requires exclusive access to a single computer with a large amount of available memory (at least 128 GB). Ray Meta produced longer contigs and more bases for these six samples. The shared content of assemblies produced by MetaVelvet and Ray Meta is shown in Table 1. A majority of sequences assembled by MetaVelvet and Ray Meta are shared. As metagenomic experiments will undoubtedly become more complex, Ray Meta will gain a distinct advantage owing to its distributed implementation.

**Table 1:** Comparison of assemblies produced by MetaVelvet and Ray Meta

	MetaVelvet	Ray Meta	Shared
ERS006494			
Reads	372,147,956		
Scaffolds^a^	50,136	56,363	
Total length (nt)	150,904,880	156,075,852	130,979,321
Average length (nt)	3,009	2,769	
N50 length (nt)	6,141	12,117	
Longest length (nt)	146,549	570,359	
ERS006497			
Reads	322,444,920		
Scaffolds^a^	61,093	52,194	
Total length (nt)	113,403,805	111,187,163	94,649,612
Average length (nt)	1,856	2,130	
N50 length (nt)	2,778	5,430	
Longest length (nt)	115,684	430,963	
Running time (h:min)	4:34	10:06	
ERS006592			
Reads	53,869,960		
Scaffolds^a^	4,358	9,387	
Total length (nt)	19,501,348	24,687,275	18,061,386
Average length (nt)	4,474	2,629	
N50 length (nt)	8,819	10,277	
Longest length (nt)	87,983	137,473	
Running time (h:min)	0:41	4:28	
SRS011098			
Reads	202,487,723		
Scaffolds^a^	30,458	36,130	
Total length (nt)	60,574,679	83,736,387	51,938,031
Average length (nt)	1,988	2,317	
N50 length (nt)	3,117	4,961	
Longest length (nt)	192,898	222,213	
Running time (h:min)	8:34	6:38	
SRS017227			
Reads	139,002,751		
Scaffolds^a^	106,957	89,953	
Total length (nt)	171,200,737	186,958,660	126,068,148
Average length (nt)	1,600	2,078	
N50 length (nt)	2,168	3,771	
Longest length (nt)	102,749	224,709	
Running time (h:min)	9:00	7:10	
SRS018661			
Reads	288,475,194		
Scaffolds^a^	30,709	18,541	
Total length (nt)	35,281,226	36,891,130	21,659,465
Average length (nt)	1,148	1,989	
N50 length (nt)	1,223	3,794	
Longest length (nt)	111,404	377,149	
Running time (h:min)	1:24	4:42	

^a^Only scaffolds with a length higher or equal to 500 were considered. nt, nucleotide.

### Validation of taxonomic profiling

We compared Ray Communities to MetaPhlAn in order to validate our methodology. Taxonomic profiles for 313 samples (Additional file 2) from the Human Microbiome Project [24] were generated with Ray Communities and compared to those of MetaPhlAn [27]. The correlations are shown in Table 2 for various body sites. Correlations are high - for instance the correlations for buccal mucosa (46 samples) were 0.99, 0.98, 0.97, 0.98, 0.95 and 0.91 for the ranks phylum, class, order, family, genus and species, respectively. These results indicate that Ray Communities has an accuracy similar to that of MetaPhlAn [27], which was utilized by the Human Microbiome Project Consortium [24]. The correlation at the genus rank for the site anterior nares was poor (0.59) because MetaPhlAn classified a high number of reads in the genus *Propionibacterium *thus yielding a very high abundance while the number of k-mer observations classified this way by Ray Communities was more moderate. For the body site called stool, the correlation at the family rank was weak (0.62) because MetaPhlAn utilizes the NCBI taxonomy whereas Ray Communities utilizes the Greengenes taxonomy, which has been shown to be more accurate [28]. Overall, these results indicate that Ray Communities yields accurate taxonomic abundances using a colored de Bruijn graph.

**Table 2:** Correlation of taxonomic abundances produced by MetaPhlAn and Ray Communities

Body site	Samples	Phylum	Class	Order	Family	Genus	Species
Anterior nares	45	0.91	0.92	0.94	0.94	0.59	0.59
Attached keratinized gingival	3	0.99	0.94	0.92	0.94	0.84	0.71
Buccal mucosa	46	0.99	0.98	0.97	0.98	0.95	0.91
Left retroauricular crease	3	0.99	0.99	0.99	0.99	0.72	0.83
Mid vagina	1	0.99	0.99	0.99	0.99	0.99	0.90
Palatine tonsils	4	0.90	0.80	0.79	0.83	0.84	0.97
Posterior fornix	23	0.99	0.99	0.99	0.99	0.97	0.94
Right retroauricular crease	6	0.94	0.92	0.93	0.94	0.83	0.91
Saliva	3	0.97	0.87	0.88	0.96	0.89	0.95
Stool	61	0.80	0.81	0.81	0.62	0.92	0.84
Subgingival plaque	5	0.86	0.75	0.76	0.74	0.81	0.93
Supragingival plaque	53	0.94	0.93	0.92	0.88	0.89	0.93
Throat	6	0.95	0.86	0.87	0.92	0.92	0.80
Tongue dorsum	53	0.93	0.80	0.79	0.84	0.85	0.88
Vaginal introitus	1	1.00	1.00	0.99	0.99	0.99	0.97
Total	313						

Pearson's correlation was utilized to compare taxonomic abundance for 313 samples from various body sites [24].

## Discussion

### Message passing

Ray Meta is a method for scalable distributed *de novo *metagenome assembly whereas MetaVelvet runs only on a single computer. Therefore, fetching data with MetaVelvet is fast because only memory accesses occur. On the other hand, Ray Meta runs on many computers. Although this is a benefit at first sight, using many computers requires messages to be sent back and forth in order to fetch data. We used 8 nodes totaling 64 processor cores (8 processor cores per node) for Human Microbiome Project samples and the observed point-to-point latency (within our application, not the hardware latency) was around 37 microseconds - this is much more than the 100 nanoseconds required for main memory accesses. However, by minimizing messages, RayMeta runs in an acceptable time and has a scalability unmatched by MetaVelvet while providing superior assemblies (Table 1).

### From Ray to Ray Meta

For single genomes, peak coverage is required by Ray in the k-mer coverage distribution [31]. This is not the case for Ray Meta. Moreover, in Ray for single genomes, read markers are selected using the peak coverage and minimum coverage. This process is local to each read path in Ray Meta. This is in theory less precise because there are fewer coverage values, but in practice it works well as shown in this work. In Ray for single genomes, the unique k-mer coverage for a seed path (similar to a unitig) is simply the peak k-mer coverage for the whole graph whereas in Ray Meta the coverage values are sampled from the seed path only.

### Algorithms for metagenome assembly

Notwithstanding the non-scalability of all *de novo *metagenome assemblers except Ray Meta (MetaVelvet [39], Meta-IDBA [40] and Genovo [41]), there are major differences in the algorithms these software tools implement, which are unrelated to scalability.

Genovo is an assembler for 454 reads. It uses a generative probabilistic model and applies a series of hill-climbing steps iteratively until convergence [41]. For Genovo, the largest dataset processed had 311,000 reads. Herein, the largest dataset had 3,000,000,000 reads. MetaVelvet and Meta-IDBA both partition the de Bruijn subgraph using k-mer coverage peaks in the k-mer coverage distribution and/or connected components. This process does not work well in theory when there is no peak in the coverage distributions. MetaVelvet and Meta-IDBA both simplify the de Bruijn graph iteratively - this approach, termed equivalent transformations, was introduced by Pevzner and collaborators [43]. One of the many advantages of using equivalent transformations is that the assembled sequences grow in length and their number decreases as the algorithm makes its way toward the final equivalent transformation. Equivalent transformations are hard to port to a distributed paradigm because the approach requires a mutable graph.

Ray Meta does not modify the de Bruijn subgraph in order to generate the assembly. We showed that applying a heuristics-guided graph traversal yields excellent assemblies. Furthermore, working with k-mers and their relationships directly is more amenable to distributed computing because unlike k-mers, contigs are neither regular nor small and are hard to load balance on numerous processes.

### Taxonomic profiling with k-mers

For taxonomic profiling, we have shown that Ray Communities is accurate when compared to MetaPhlAn (Table 2). Our approach consists in building a de Bruijn graph from the raw sequencing reads, assembling it *de novo*, and then coloring it with thousands of bacterial genomes in order to obtain an accurate profile of the sequenced metagenome. By using whole genomes instead of a few selected marker genes, such as the 16S RNA gene, some biases are removed (like the copy number of a gene). Furthermore, amplifications in a whole-genome sequencing protocol are not targeted toward any particular marker genes, which may remove further biases. A limitation of the method presented here is that using k-mers alone to compare sequences is highly stringent. On the other hand, aligner-based approaches can accommodate for an identity as low as 70% between sequences as sequence reads are usually mapped to reference bacterial genomes. At the crux of our method is the use of uniquely colored k-mers for signal demultiplexing (see Materials and methods). Sequencing errors produce erroneous k-mers. One of the advantages of using a de Bruijn graph is that erroneous k-mers have a small probability of being considered in the assembly [31], hence sequencing errors do not contribute to taxonomic profiling for assembled sequences. However, alignment-based approaches will likely a higher sensitivity than k-mer based approaches because they are more tolerant to mismatches. Yet, the present work showed that metagenome profiling is efficiently done with k-mer counting, through the use of a colored de Bruijn graph [7], and that it is also sensitive (Figure 2) and produces results similar to those of MetaPhlAn (Table 2). With this approach, conserved DNA regions captured the biological abundance of bacteria in a sample. A k-mer length of 31 was used to give a high stringency in the coloring process. The low error rate of the sequencing technology enabled the capture of error-free k-mers for most of the genomic regions, meaning that it was unlikely that a given k-mer occurred in the sequence reads, in a known genome, but not in the actual sample.

### Validation of assemblies

Using MUMmer [44], we validated the quality of assemblies produced by Ray Meta. The quality test used was very stringent because any contig not aligning as one single maximum unique match with a breadth of coverage of at least 98% was marked as misassembled. In Table 1, the number of shared k-mers between assemblies produced by MetaVelvet and Ray Meta is shown. Although the overlap is significant, the k-mers unique to MetaVelvet or Ray Meta may be due to nucleotide mismatches. Moreover, improvements in sequencing technologies will provide longer reads with higher coverage depths. These advances will further improve *de novo *assemblies.

## Conclusions

Scalability is a requirement for analyzing large metagenome datasets. We described a new method to assemble (Ray Meta) and profile (Ray Communities) a metagenome in a distributed fashion to provide unmatched scalability. It computes a metagenome *de novo *assembly in parallel with a de Bruijn graph. The method also yields taxonomic profiles by coloring the graph with known references and by looking for uniquely colored k-mers to identify taxons at low taxonomic ranks or by using the lowest common ancestor otherwise. Ray Meta surpassed MetaVelvet [39] for *de novo *assemblies and Ray Communities compared favorably to MetaPhlAn [27] for taxonomic profiling.

While taxonomic and functional profiling remains a useful approach to obtain a big picture of a particular sample, only *de novo *metagenome assembly can truly enable discovery of otherwise unknown genes or other important DNA sequences hidden in the data.

## Materials and methods

Thorough documentation and associated scripts to reproduce our studies are available in Additional file 3 on the publisher website or on https://github.com/sebhtml/Paper-Replication-2012.

### Memory model

Ray Meta uses the message-passing interface. As such, a 1,024-core job has 1,024 processes running on many computers. In the experiments, each node had 8 processor cores and 24 GB, or 3 GB per core. With the message-passing paradigm, each core has its own virtual memory, which is protected from any other process. Because the data are distributed uniformly using a distributed hash table, memory usage for a single process is very low. For the 1,024-core job, the maximum memory usage of any process was on average 1.5 GB.

### Assemblies

Metagenome assemblies with profiling were computed with Ray v2.0.0 (Additional file 4) on Colosse, a Compute Canada resource. Ray is open source software - the license is the GNU General Public License, version 3 (GPLv3) - and is freely available from http://denovoassembler.sourceforge.net/ or http://github.com/sebhtml/ray. Ray can be deployed on public compute infrastructure or in the cloud (see [45] for a review).

The algorithms implemented in the software Ray were heavily modified for metagenome *de novo *assembly and these changes were called Ray Meta. Namely, the coverage distribution for k-mers in the de Bruijn graph is not utilized to infer the average coverage depth for unique genomic regions. Instead, this value is derived from local coverage distributions during the parallel assembly process. Therefore, unlike MetaVelvet [39], Ray Meta does not attempt to calculate or use any global k-mer coverage depth distribution.

### Simulated metagenomes with a power law

Two metagenomes (100 and 1,000 genomes, respectively) were simulated with abundances following a power law (Tables S1 and S2 in Additional file 1). Power laws are commonly found in biological systems [46]. Simulated sequencing errors were randomly distributed, the error rate was set at 0.25% and the average insert length was 400. The second simulated metagenome was assembled with 128 8-core computers (1,024 processor cores) interconnected with a Mellanox ConnectX QDR Infiniband fabric (Mellanox, Inc.). For the 1,000-genome dataset, messages were routed with a de Bruijn graph of degree 32 and diameter 2 to reduce the latency.

### Validation of assemblies

Assembled contigs were aligned onto reference genomes using the MUMmer bioinformatics software suite [44]. More precisely, deltas were generated with nucmer. Using show-coords, any contig not aligning as one single maximum with at least 98% breadth of coverage was marked as misassembled. Contigs aligning in two parts at the beginning and end of a reference were not counted as misassembled owing to the circular nature of bacterial genomes. Finally, small insertions/deletions and mismatches were obtained with show-SNPs.

### Colored and distributed de Bruijn graphs

The vertices of a de Bruijn graph are distributed across processes called ranks. Here, graph coloring means labeling the vertices of a graph. A different color is added to the graph for each reference sequence. Each k-mer in any reference sequence is colored with the reference sequence color if it occurs in the distributed de Bruijn graph. Therefore, any k-mer in the graph has zero, one or more colors. First, a k-mer with no colors indicates that the k-mer does not exist in the databases provided. Second, a k-mer with one color means that this k-mer is specific to one and only one reference genome in the databases provided while at least two colors indicates that the k-mer is not specific to one single reference sequence. These reference sequences are assigned to leaves in a taxonomic tree. Reference sequences can be grouped in independent name spaces. Genome assembly is independent of graph coloring.

### Demultiplexing signals from similar bacterial strains

Biological abundances were estimated using the product of the number of k-mers matched in the distributed de Bruijn graph by the mode coverage of k-mers that were uniquely colored. This number is called the number of k-mer observations. The total number of k-mer observations is the sum of coverage depth values of all colored k-mers. A proportion is calculated by dividing the number of k-mer observations by the total.

### Taxonomic profiling

All bacterial genomes available in GenBank [47] were utilized for coloring the distributed de Bruijn graphs (Table S4 in Additional file 1). Each k-mer was assigned to a taxon in the taxonomic tree. When a k-mer has more than one taxon color, the coverage depth was assigned to the nearest common ancestor.

### Gene ontology profiling

The de Bruijn graph was colored with coding sequences from the EMBL nucleotide sequence database [48] (EMBL_CDS), which are mapped to gene ontology by transitivity using the uniprot mapping to gene ontology [49]. For each ontology term, coverage depths of colored k-mers were added to obtain the total number of k-mer observations.

### Principal component analysis

Principal component analysis was used to group taxonomic profiles to produce enterotypes. Data were prepared in a matrix using the genera as rows and the samples as columns. Singular values and left and right singular vectors of this matrix were obtained using singular value decomposition implemented in R. The right singular vectors were sorted by singular values. The sorted right singular vectors were used as the new base for the re-representation of the genus proportions. The two first dimensions were plotted.

### Software implementation

Ray Meta is distributed software that runs on connected computers by transmitting messages over a network using the message-passing interface (MPI) and is implemented in C++. The MPI standard is implemented in libraries such as Open-MPI [50] and MPICH2 [51]. On each processor core, tasks are divided into smaller ones and given to a pool of 32,768 workers (thread pool), which are similar to chares in CHARM++ [52]. Each of these sends messages to a virtual communicator. The latter implements a message aggregation strategy in which messages are automatically multiplexed and demultiplexed. The k-mers are stored in a distributed sparse hash table which utilizes open addressing (double hashing) for collisions. Incremental resizing is utilized in this hash table when the occupancy exceeds 90% to grow tables locally. Smart pointers are utilized in this table to perform real-time memory compaction. The software is implemented on top of RayPlatform, a development framework used to ease the creation of massively distributed high-performance computing applications.

### Comparison with MetaVelvet

Software versions used were: MetaVelvet 1.2.01, Velvet 1.2.07 and Ray 2.0.0 (with Ray Meta). MetaVelvet was run on one processor core. Ray Meta was run on 64 processor cores for Human Microbiome Project samples (SRS011098, SRS017227 and SRS018661) and on 48, 32 and 32 processor cores for MetaHIT samples (ERS006494, ERS006497 and ERS006592), respectively. There were eight processor cores per node. The running time for MetaVelvet is the sum of running times for velveth, velvetg and meta-velvetg. For MetaVelvet, sequence files were filtered to remove any sequence with more than 10*N *symbols. The resulting files were shuffled to create files with interleaved sequences. The insert size was manually provided to MetaVelvet and the k-mer length was set to 51 as suggested in its documentation. Peak coverages were determined automatically by MetaVelvet. Ray Meta was run with a k-mer length of 31. No other parameters were required for Ray Meta and sequence files were provided without modification to Ray Meta. The overlaps of assemblies produced by MetaVelvet and by Ray Meta were evaluated with Ray using the graph coloring features. No mismatches were allowed in k-mers. Overlaps were computed for scaffolds with at least 500 nucleotides.

### Comparison with MetaPhlAn

Taxonomic profiles calculated with MetaPhlAn [27] for samples from the Human Microbiome Project were obtained [24]. Taxonomic profiles were produced by Ray Communities for 313 samples (Additional file 2). Pearson's correlation was calculated for each body site by combining taxon proportions for both methods for each taxonomic rank.

## Abbreviations

MPI: message-passing interface; nt: nucleotide.

## Competing interests

The authors declare that they have no competing interests.

## Authors' contributions

SB drafted the manuscript, implemented methods, gathered public data and performed simulations and analyses. SB, JC and FR analyzed results. SB, FL and JC designed *de novo *assembly algorithms. SB and FR designed graph coloring strategies. EG and SB devised parallel distributed software designs. All authors read and approved the final manuscript.

## Supplementary Material

Additional file 1 **Tables S1, S2, S3 & S4**. Table S1: Composition of the simulated 100-genome metagenome. Table S2: Composition of the simulated 1,000-genome metagenome. Table S3: Overlay data on metagenome assembly of 124 gut microbiome samples. Table S4: List of genomes used for coloring de Bruijn graphs. Click here for file

Additional file 2 **List of 313 samples from the Human Microbiome Project**. Click here for file

Additional file 3 **Documentation and scripts to reproduce all experiments**. Click here for file

Additional file 4 **Software source code for Ray Meta and Ray Communities**. Click here for file

## References

[B1] Wold B Myers RM Sequence census methods for functional genomics. Nature Methods 2008 13 19 21 10.1038/nmeth1157 18165803

[B2] Brenner S Sequences and consequences. Philosophical Transactions of the Royal Society B: Biological Sciences 2010 13 207 212 10.1098/rstb.2009.0221 PMC2842711 20008397

[B3] McPherson JD Next-generation gap. Nature Methods 2009 13 S2 S5 10.1038/nmeth.f.268 19844227

[B4] Mardis E The $1,000 genome, the $100,000 analysis?. Genome Medicine 2010 13 84 10.1186/gm205 21114804 PMC3016626

[B5] Compeau PEC Pevzner PA Tesler G How to apply de Bruijn graphs to genome assembly. Nature Biotechnology 2011 13 987 991 10.1038/nbt.2023 22068540 PMC5531759

[B6] Flicek P Birney E Sense from sequence reads: methods for alignment and assembly. Nature Methods 2009 13 S6 S12 10.1038/nmeth.1376 19844229

[B7] Iqbal Z Caccamo M Turner I Flicek P McVean G *De novo *assembly and genotyping of variants using colored de Bruijn graphs. Nature Genetics 2012 13 226 232 10.1038/ng.1028 22231483 PMC3272472

[B8] Miller JR Koren S Sutton G Assembly algorithms for next-generation sequencing data. Genomics 2010 13 315 327 10.1016/j.ygeno.2010.03.001 20211242 PMC2874646

[B9] Salzberg SL Beware of mis-assembled genomes. Bioinformatics 2005 13 4320 4321 10.1093/bioinformatics/bti769 16332717

[B10] Treangen TJ Salzberg SL Repetitive DNA and next-generation sequencing: computational challenges and solutions. Nature Reviews Genetics 2011 13 36 46 22124482 10.1038/nrg3117 PMC3324860

[B11] Lorenz P Eck J Metagenomics and industrial applications. Nature Reviews Microbiology 2005 13 510 516 10.1038/nrmicro1161 15931168

[B12] Scholz MB Lo CC Chain PSG Next generation sequencing and bioinformatic bottlenecks: the current state of metagenomic data analysis. Current Opinion in Biotechnology 2012 13 9 15 10.1016/j.copbio.2011.11.013 22154470

[B13] Schoenfeld T Patterson M Richardson PM Wommack KE Young M Mead D Assembly of viral metagenomes from Yellowstone Hot Springs. Applied and Environmental Microbiology 2008 13 4164 4174 10.1128/AEM.02598-07 18441115 PMC2446518

[B14] Varin T Lovejoy C Jungblut AD Vincent WF Corbeil J Metagenomic analysis of stress genes in microbial mat communities from Antarctica and the high Arctic. Applied and Environmental Microbiology 2012 13 549 559 10.1128/AEM.06354-11 22081564 PMC3255749

[B15] Varin T Lovejoy C Jungblut AD Vincent WF Corbeil J Metagenomic profiling of Arctic microbial mat communities as nutrient scavenging and recycling systems. Limnology and Oceanography 2010 13 1901 1911 10.4319/lo.2010.55.5.1901

[B16] Narasingarao P Podell S Ugalde JA Brochier-Armanet C Emerson JB Brocks JJ Heidelberg KB Banfield JF Allen EE *De novo *metagenomic assembly reveals abundant novel major lineage of Archaea in hypersaline microbial communities. The ISME Journal 2011 13 81 93 21716304 10.1038/ismej.2011.78 PMC3246234

[B17] Tringe SG von Mering C Kobayashi A Salamov AA Chen K Chang HW Podar M Short JM Mathur EJ Detter JC Bork P Hugenholtz P Rubin EM Comparative metagenomics of microbial communities. Science 2005 13 554 557 10.1126/science.1107851 15845853

[B18] Tyson GW Chapman J Hugenholtz P Allen EE Ram RJ Richardson PM Solovyev VV Rubin EM Rokhsar DS Banfield JF Community structure and metabolism through reconstruction of microbial genomes from the environment. Nature 2004 13 37 43 10.1038/nature02340 14961025

[B19] Naviaux RK Good B McPherson JD Steffen DL Markusic D Ransom B Corbeil J Sand DNA - a genetic library of life at the water's edge. Marine Ecology Progress Series 2005 13 9 22

[B20] Cho I Blaser MJ The human microbiome: at the interface of health and disease. Nature Reviews Genetics 2012 13 260 270 22411464 10.1038/nrg3182 PMC3418802

[B21] Gill SR Pop M Deboy RT Eckburg PB Turnbaugh PJ Samuel BS Gordon JI Relman DA Fraser-Liggett CM Nelson KE Metagenomic analysis of the human distal gut microbiome. Science 2006 13 1355 1359 10.1126/science.1124234 16741115 PMC3027896

[B22] Qin J Li R Raes J Arumugam M Burgdorf KS Manichanh C Nielsen T Pons N Levenez F Yamada T Mende DR Li J Xu J Li S Li D Cao J Wang B Liang H Zheng H Xie Y Tap J Lepage P Bertalan M Batto JM Hansen T Le Paslier D Linneberg A Nielsen HB Pelletier E Renault P A human gut microbial gene catalogue established by metagenomic sequencing. Nature 2010 13 59 65 10.1038/nature08821 20203603 PMC3779803

[B23] Arumugam M Raes J Pelletier E Le Paslier D Yamada T Mende DR Fernandes GR Tap J Bruls T Batto JMM Bertalan M Borruel N Casellas F Fernandez L Gautier L Hansen T Hattori M Hayashi T Kleerebezem M Kurokawa K Leclerc M Levenez F Manichanh C Nielsen HB Nielsen T Pons N Poulain J Qin J Sicheritz-Ponten T Tims S Enterotypes of the human gut microbiome. Nature 2011 13 174 180 10.1038/nature09944 21508958 PMC3728647

[B24] Consortium THMP Structure, function and diversity of the healthy human microbiome. Nature 2012 13 207 214 10.1038/nature11234 22699609 PMC3564958

[B25] Schloss PD Handelsman J Introducing DOTUR, a computer program for defining operational taxonomic units and estimating species richness. Applied and Environmental Microbiology 2005 13 1501 1506 10.1128/AEM.71.3.1501-1506.2005 15746353 PMC1065144

[B26] Liu B Gibbons T Ghodsi M Pop M MetaPhyler: taxonomic profiling for metagenomic sequences. 2010 IEEE International Conference on Bioinformatics and Biomedicine (BIBM) 2010 IEEE 95 100

[B27] Segata N Waldron L Ballarini A Narasimhan V Jousson O Huttenhower C Metagenomic microbial community profiling using unique clade-specific marker genes. Nature Methods 2012 13 811 814 10.1038/nmeth.2066 22688413 PMC3443552

[B28] McDonald D Price MN Goodrich J Nawrocki EP DeSantis TZ Probst A Andersen GL Knight R Hugenholtz P An improved Greengenes taxonomy with explicit ranks for ecological and evolutionary analyses of bacteria and archaea. The ISME Journal 2011 13 610 618 22134646 10.1038/ismej.2011.139 PMC3280142

[B29] Ashburner M Ball CA Blake JA Botstein D Butler H Cherry JM Davis AP Dolinski K Dwight SS Eppig JT Harris MA Hill DP Issel-Tarver L Kasarskis A Lewis S Matese JC Richardson JE Ringwald M Rubin GM Sherlock G Gene ontology: tool for the unification of biology. The Gene Ontology Consortium. Nature Genetics 2000 13 25 29 10.1038/75556 10802651 PMC3037419

[B30] Simpson JT Wong K Jackman SD Schein JE Jones SJM Birol I ABySS: a parallel assembler for short read sequence data. Genome Research 2009 13 1117 1123 10.1101/gr.089532.108 19251739 PMC2694472

[B31] Boisvert S Laviolette F Corbeil J Ray: simultaneous assembly of reads from a mix of high-throughput sequencing technologies. Journal of Computational Biology 2010 13 1519 1533 10.1089/cmb.2009.0238 20958248 PMC3119603

[B32] Schatz MC Langmead B Salzberg SL Cloud computing and the DNA data race. Nature Biotechnology 2010 13 691 693 10.1038/nbt0710-691 20622843 PMC2904649

[B33] Huson DH Mitra S Ruscheweyh HJ Weber N Schuster SC Integrative analysis of environmental sequences using MEGAN4. Genome Research 2011 13 1552 1560 10.1101/gr.120618.111 21690186 PMC3166839

[B34] Meyer F Paarmann D D'Souza M Olson R Glass EM Kubal M Paczian T Rodriguez A Stevens R Wilke A Wilkening J Edwards RA The etagenomics RAST server - a public resource for the automatic phylogenetic and functional analysis of metagenomes. BMC Bioinformatics 2008 13 386 388 10.1186/1471-2105-9-386 18803844 PMC2563014

[B35] Dixon P VEGAN, a package of R functions for community ecology. Journal of Vegetation Science 2003 13 927 930 10.1111/j.1654-1103.2003.tb02228.x

[B36] Caporaso JG Kuczynski J Stombaugh J Bittinger K Bushman FD Costello EK Fierer N Pena AG Goodrich JK Gordon JI Huttley GA Kelley ST Knights D Koenig JE Ley RE Lozupone CA McDonald D Muegge BD Pirrung M Reeder J Sevinsky JR Turnbaugh PJ Walters WA Widmann J Yatsunenko T Zaneveld J Knight R QIIME allows analysis of high-throughput community sequencing data. Nature Methods 2010 13 335 336 10.1038/nmeth.f.303 20383131 PMC3156573

[B37] Krause L Diaz NN Goesmann A Kelley S Nattkemper TW Rohwer F Edwards RA Stoye J Phylogenetic classification of short environmental DNA fragments. Nucleic Acids Research 2008 13 2230 2239 10.1093/nar/gkn038 18285365 PMC2367736

[B38] Brady A Salzberg SL Phymm and PhymmBL: metagenomic phylogenetic classification with interpolated Markov models. Nature Methods 2009 13 673 676 10.1038/nmeth.1358 19648916 PMC2762791

[B39] Namiki T Hachiya T Tanaka H Sakakibara Y MetaVelvet: an extension of Velvet assembler to *de novo *metagenome assembly from short sequence reads. Nucleic Acids Research 2012 13 e155 10.1093/nar/gks678 22821567 PMC3488206

[B40] Peng Y Leung HCM Yiu SM Chin FYL Meta-IDBA: a *de novo *assembler for metagenomic data. Bioinformatics 2011 13 i94 i101 10.1093/bioinformatics/btr216 21685107 PMC3117360

[B41] Laserson J Jojic V Koller D Genovo: de novo assembly for metagenomes. Journal of Computational Biology 2011 13 429 443 10.1089/cmb.2010.0244 21385045

[B42] Wu GD Chen J Hoffmann C Bittinger K Chen YYY Keilbaugh SA Bewtra M Knights D Walters WA Knight R Sinha R Gilroy E Gupta K Baldassano R Nessel L Li H Bushman FD Lewis JD Linking long-term dietary patterns with gut microbial enterotypes. Science (New York, NY) 2011 13 105 108 10.1126/science.1208344 PMC3368382 21885731

[B43] Pevzner PA Tang H Waterman MS An Eulerian path approach to DNA fragment assembly. Proceedings of the National Academy of Sciences 2001 13 9748 9753 10.1073/pnas.171285098 PMC55524 11504945

[B44] Kurtz S Phillippy A Delcher AL Smoot M Shumway M Antonescu C Salzberg SL Versatile and open software for comparing large genomes. Genome Biol 2004 13 R12 10.1186/gb-2004-5-2-r12 14759262 PMC395750

[B45] Schadt EE Linderman MD Sorenson J Lee L Nolan GP Computational solutions to large-scale data management and analysis. Nature Reviews Genetics 2010 13 647 657 20717155 10.1038/nrg2857 PMC3124937

[B46] Barabasi AL Oltvai ZN Network biology: understanding the cell's functional organization. Nature Reviews Genetics 2004 13 101 113 10.1038/nrg1272 14735121

[B47] Benson DA Boguski MS Lipman DJ Ostell J GenBank. Nucleic Acids Research 1997 13 1 6 10.1093/nar/25.1.1 9016491 PMC146400

[B48] Kulikova T Aldebert P Althorpe N Baker W Bates K Browne P van den Broek A Cochrane G Duggan K Eberhardt R Faruque N Garcia-Pastor M Harte N Kanz C Leinonen R Lin Q Lombard V Lopez R Mancuso R McHale M Nardone F Silventoinen V Stoehr P Stoesser G Ann M Tzouvara K Vaughan R Wu D Zhu W Apweiler R The EMBL nucleotide sequence database. Nucleic Acids Research 2004 13 D27 30 10.1093/nar/gkh120 14681351 PMC308854

[B49] Camon E Magrane M Barrell D Lee V Dimmer E Maslen J Binns D Harte N Lopez R Apweiler R The gene ontology annotation (GOA) database: sharing knowledge in Uniprot with gene ontology. Nucleic Acids Research 2004 13 D262 266 10.1093/nar/gkh021 14681408 PMC308756

[B50] Gabriel E Fagg G Bosilca G Angskun T Dongarra J Squyres J Sahay V Kambadur P Barrett B Lumsdaine A Castain R Daniel D Graham R Woodall T Gabriel E Fagg GE Bosilca G Angskun T Dongarra JJ Squyres JM Sahay V Kambadur P Barrett B Lumsdaine A Castain RH Daniel DJ Graham RL Woodall TS Kranzlmüller D, Kacsuk P, Dongarra J. Berlin, Heidelberg Open MPI: goals, concept, and design of a next generation MPI implementation recent advances in parallel virtual machine and message massing interface. Recent Advances in Parallel Virtual Machine and Message Passing Interface, Volume 3241 of Lecture Notes in Computer Science 13 Springer Berlin/Heidelberg 353 377

[B51] Gropp W Kranzlmüller D, Volkert J, Kacsuk P, Dongarra J. Berlin, Heidelberg MPICH2: A new start for MPI implementations. Recent Advances in Parallel Virtual Machine and Message Passing Interface, Volume 2474 of Lecture Notes in Computer Science 2002 Springer Berlin/Heidelberg 37 42

[B52] Kale LV Krishnan S CHARM++: a portable concurrent object oriented system based on C++. Proceedings of the 8th Annual Conference on Object-Oriented Programming Systems, Languages, and Applications, OOPSLA '93, New York, NY, USA 1993 ACM 91 108

