# Supplementary material for: Ray Meta: scalable de novo metagenome assembly and profiling
Source: Genome Biol. 2012 Dec 22;13(12):R122. doi: 10.1186/gb-2012-13-12-r122 (PMC4056372; doi:10.1186/gb-2012-13-12-r122)
Supplement: Additional file 1 — Tables S1, S2, S3 & S4. Table S1: Composition of the simulated 100-genome metagenome. Table S2: Composition of the simulated 1,000-genome metagenome. Table S3: Overlay data on metagenome assembly of 124 gut microbiome samples. Table S4: List of genomes used for coloring de Bruijn graphs. [file gb-2012-13-12-r122-S1.PDF]

Supplementary Table 1: Composition of the simulated 100-genome metagenome. This metagenome contains 400×10^6 101-nucleotide reads. This corresponds to 1 lane of a Illumina HiSeq 2000 flow cell. The reads are paired with a average fragment length of 400 and a standard deviation of 40. The substitution sequencing error rate is 0.25% and uniform. 1% of the reads are human sequences. The remaining numbers of reads are distributed with a power law (exponent: -0.5). Accession numbers are listed in Supplementary Table 4

Genome	Number of reads
Homo sapiens (hg19)	4000000
Oligotropha carboxidovorans OM5	21302230
Wolbachia endosymbiont of Drosophila melanogaster	15062950
Klebsiella variicola At 22	12298848
Chlorobium luteolum DSM 273	10651114
Borrelia afzelii PKo	9526646
Francisella tularensis mediasiatica FSC147	8696598
Aliivibrio salmonicida LFI1238	8051486
Salmonella enterica serovar Typhi CT18	7531476
Laribacter hongkongensis HLHK9	7100744
Sulfurimonas autotrophica DSM 16294	6736356
Odoribacter splanchnicus DSM 20712	6422864
Sulfolobus islandicus Y N 15 51	6149424
Roseobacter denitrificans OCh 114	5908176
Hyphomonas neptunium ATCC 15444	5693260
Clostridium botulinum Ba4 657	5500212
Nitrosopumilus maritimus SCM1	5325558
Yersinia pestis CO92	5166550
Haloarcula hispanica ATCC 33960	5020984
Thioalkalivibrio K90mix	4887066
Thermoanaerobacter italicus Ab9	4763324
Yersinia enterocolitica palearctica 105 5R r	4648528
Halorhabdus utahensis DSM 12940	4541650
Amycolicicoccus subflavus DQS3 9A1	4441822
Salmonella enterica serovar Paratyphi C RKS4594	4348300
Streptomyces violaceusniger Tu 4113	4260446
Pseudomonas fluorescens Pf 5	4177710
Amycolatopsis mediterranei U32	4099616
Chlorobium phaeobacteroides DSM 266	4025742
Leifsonia xyli CTCB07	3955724
Sinorhizobium medicae WSM419	3889238
Escherichia coli ATCC 8739	3825994
Burkholderia phymatum STM815	3765738
Haloferax volcanii DS2	3708242
Variovorax paradoxus EPS	3653302
Helicobacter hepaticus ATCC 51449	3600734
Delftia acidovorans SPH 1	3550372

Coxiella burnetii RSA 493	3502064
Pseudomonas mendocina ymp	3455678
Rhodopseudomonas palustris HaA2	3411086
Candidatus Moranella endobia PCIT	3368178
Archaeoglobus profundus DSM 5631	3326850
Megasphaera elsdenii DSM 20460	3287006
Candidatus Vesicomyosocius okutanii HA	3248560
Yersinia pestis Pestoides F	3211432
Chlorobium chlorochromatii CaD3	3175548
Burkholderia glumae BGR1	3140842
Xylella fastidiosa M12	3107250
Synechococcus elongatus PCC 6301	3074712
Acinetobacter ADP1	3043176
Halanaerobium hydrogeniformans	3012590
Leptospira interrogans serovar Lai 56601	2982908
Serratia AS9	2954088
Riemerella anatipestifer DSM 15868	2926086
Lacinutrix 5H 3 7 4	2898866
Paenibacillus JDR 2	2872392
Geobacillus Y412MC61	2846630
Rhodobacter sphaeroides ATCC 17025	2821550
Dehalococcoides BAV1	2797120
Acinetobacter DR1	2773314
Halomicrobium mukohataei DSM 12286	2750106
Prevotella ruminicola 23	2727470
Desulfovibrio vulgaris Hildenborough	2705386
Idiomarina loihiensis L2TR	2683828
Desulfatibacillum alkenivorans AK 01	2662778
Teredinibacter turnerae T7901	2642216
Haemophilus influenzae PittGG	2622124
Candidatus Amoebophilus asiaticus 5a2	2602482
Psychrobacter cryohalolentis K5	2583274
Clostridium SY8519	2564488
Parvibaculum lavamentivorans DS 1	2546104
Synechococcus CC9605	2528110
Prochlorococcus marinus MIT 9211	2510492
Rhodoferax ferrireducens T118	2493238
Salmonella enterica serovar Heidelberg SL476	2476334
Clostridium difficile R20291	2459770
Helicobacter pylori P12	2443534
Escherichia coli S88	2427614
Thermococcus sibiricus MM 739	2412002
Bacillus amyloliquefaciens FZB42	2396688
Aquifex aeolicus VF5	2381662
Streptococcus gallolyticus UCN34	2366914
Prochlorococcus marinus MIT 9215	2352438

Pyrococcus horikoshii OT3	2338224
Methanocaldococcus vulcanius M7	2324264
Escherichia coli K 12 substr MG1655	2310552
Methanococcus maripaludis X1	2297078
Dickeya zeae Ech1591	2283838
Pseudomonas syringae B728a	2270826
Rhodopseudomonas palustris BisA53	2258032
Cellulophaga lytica DSM 7489	2245452
Neisseria gonorrhoeae NCCP11945	2233080
Rothia dentocariosa ATCC 17931	2220910
Acidimicrobium ferrooxidans DSM 10331	2208938
Shewanella ANA 3	2197158
Neisseria gonorrhoeae FA 1090	2185562
Brucella suis ATCC 23445	2174150
Mobiluncus curtisii ATCC 43063	2162914
Methylomicrobium alcaliphilum	2151850
Staphylothermus hellenicus DSM 12710	2140954
Olsenella uli DSM 7084	2130222

Supplementary Table 2: Composition of the simulated 1000-genome metagenome. This metagenome contains 3×10^9 100-nucleotide reads. This corresponds to 1 Illumina HiSeq 2000 flow cell. The reads are paired with a average fragment length of 400 and a standard deviation of 40. The substitution sequencing error rate is 0.25% and uniform. 1% of the reads are human sequences. The remaining numbers of reads are distributed with a power law (exponent: -0.3). Accession numbers are listed in Supplementary Table 4

Genome	Number of reads
Homo sapiens (hg19)	30000000
Pseudogulbenkiania NH8B	48057468
Methanosaeta concilii GP6	33981762
Paenibacillus polymyxa SC2	27745992
Myxococcus fulvus HW 1	24028734
Desulfotomaculum carboxydivorans CO 1 SRB	21491954
Xylella fastidiosa M12	19619380
Ruminococcus albus 7	18164016
Bacillus anthracis A0248	16990880
Mesorhizobium opportunistum WSM2075	16019156
Corynebacterium resistens DSM 45100	15197106
Chlamydia trachomatis B TZ1A828 OT	14489872
Ilyobacter polytropus DSM 2926	13872996
Synechococcus PCC 7002	13328744
Halomicrobium mukohataei DSM 12286	12843898
Chloroflexus aggregans DSM 9485	12408384
Brachybacterium faecium DSM 4810	12014366
Bacillus clausii KSM K16	11655648
Thermocrinis albus DSM 14484	11327254
Glaciecola nitratireducens FR1064	11025140
Corynebacterium glutamicum R	10745976
Acinetobacter baumannii ACICU	10487000
Thermoplasma acidophilum DSM 1728	10245886
Dichelobacter nodosus VCS1703A	10020674
Pseudomonas aeruginosa UCBPP PA14	9809690
Xanthomonas albilineans GPE PC73	9611494
Starkeya novella DSM 506	9424844
Pediococcus pentosaceus ATCC 25745	9248664
Dyadobacter fermentans DSM 18053	9082008
Candidatus Liberibacter asiaticus psy62	8924048
Onion yellows phytoplasma OY M	8774054
Gluconobacter oxydans 621H	8631376
Cellulomonas flavigena DSM 20109	8495440
Lactobacillus gasseri ATCC 33323	8365732
Eggerthella lenta DSM 2243	8241788
Rubrobacter xylanophilus DSM 9941	8123194
Nitrosococcus oceani ATCC 19707	8009578
Haloterrigena turkmenica DSM 5511	7900600

Treponema denticola ATCC 35405	7795950
Riemerella anatipestifer DSM 15868	7695354
Cellvibrio japonicus Ueda107	7598552
Buchnera aphidicola Bp Baizongia pistaciae	7505316
Geobacter bemidjiensis Bem	7415428
Halorubrum lacusprofundi ATCC 49239	7328696
Listeria monocytogenes serotype 4b F2365	7244936
Methanocaldococcus vulcanius M7	7163984
Amycolatopsis mediterranei U32	7085688
Methanosarcina barkeri Fusaro	7009902
Campylobacter concisus 13826	6936498
Agrobacterium vitis S4	6865352
Atopobium parvulum DSM 20469	6796352
Deinococcus radiodurans R1	6729392
Lawsonia intracellularis PHE MN1 00	6664372
Escherichia coli BW2952	6601200
Enterobacter 638	6539794
Acidobacterium capsulatum ATCC 51196	6480068
Borrelia burgdorferi B31	6421950
Rhodococcus opacus B4	6365368
Haemophilus somnus 129PT	6310254
Candidatus Desulforudis audaxviator MP104C	6256550
Desulfovibrio vulgaris Hildenborough	6204192
Azotobacter vinelandii DJ	6153128
Synechocystis PCC 6803	6103304
halophilic archaeon DL31	6054672
Treponema pallidum Nichols	6007184
Streptococcus pneumoniae G54	5960796
Candidatus Zinderia insecticola CARI	5915466
Desulfovibrio aespoeensis Aspo 2	5871154
Lactobacillus acidophilus 30SC	5827824
Nitrosopumilus maritimus SCM1	5785440
Salmonella bongori NCTC 12419	5743966
Pseudoxanthomonas suwonensis 11 1	5703372
Aerococcus urinae ACS 120 V Col10a	5663626
Corynebacterium variabile DSM 44702	5624702
Listeria welshimeri serovar 6b SLCC5334	5586568
Xanthomonas oryzae MAFF 311018	5549198
Methylobacterium extorquens DM4	5512570
Rhodococcus erythropolis PR4	5476656
Thiobacillus denitrificans ATCC 25259	5441436
Cellvibrio gilvus ATCC 13127	5406888
Bartonella henselae Houston 1	5372988
Bradyrhizobium japonicum USDA 110	5339718
Wolbachia endosymbiont of Drosophila melanogaster	5307060
Chlamydophila pneumoniae TW 183	5274992

Synechococcus WH 8102	5243500
Campylobacter fetus 82 40	5212564
Spirosoma linguale DSM 74	5182170
Lactobacillus kefiranofaciens ZW3	5152302
Halalkalicoccus jeotgali B3	5122944
Streptococcus thermophilus LMG 18311	5094082
Campylobacter jejuni 81116	5065702
Escherichia coli O26 H11 11368	5037792
Helicobacter pylori SJM180	5010338
Nitrobacter hamburgensis X14	4983328
Clostridiales genomosp BVAB3 UPII9 5	4956750
Geobacter FRC 32	4930592
Alteromonas macleodii Deep ecotype	4904844
Thermotoga RQ2	4879496
Thermoproteus neutrophilus V24Sta	4854538
Haemophilus somnus 2336	4829958
Herbaspirillum seropedicae SmR1	4805746
Thermodesulfatator indicus DSM 15286	4781896
Escherichia coli K 12 substr MG1655	4758398
Nocardiopsis dassonvillei DSM 43111	4735244
Chlamydia muridarum Nigg	4712422
Clostridium botulinum A Hall	4689928
Pseudomonas stutzeri A1501	4667754
Helicobacter mustelae 12198	4645890
Opitutus terrae PB90 1	4624332
Syntrophomonas wolfei Goettingen	4603070
Geobacillus WCH70	4582100
Candidatus Tremblaya princeps PCIT	4561412
Methylotenera 301	4541004
Listeria monocytogenes HCC23	4520866
Bacillus cellulosilyticus DSM 2522	4500994
Hyperthermus butylicus DSM 5456	4481382
Yersinia enterocolitica palearctica 105 5R r	4462024
Methanococcoides burtonii DSM 6242	4442914
Rhodospirillum centenum SW	4424048
Leptospira borgpetersenii serovar Hardjo bovis L550	4405420
Cronobacter turicensis z3032	4387026
Alteromonas SN2	4368860
Anaerococcus prevotii DSM 20548	4350918
Isosphaera pallida ATCC 43644	4333196
Thermomicrobium roseum DSM 5159	4315688
Sanguibacter keddieii DSM 10542	4298390
Acidimicrobium ferrooxidans DSM 10331	4281300
Gardnerella vaginalis ATCC 14019	4264410
Corynebacterium kroppenstedtii DSM 44385	4247720
Microbacterium testaceum StLB037	4231224

Escherichia coli HS	4214918
Pseudomonas fluorescens Pf 5	4198800
Streptococcus pyogenes SSI 1	4182866
Salmonella enterica serovar Typhi Ty2	4167110
Campylobacter jejuni RM1221	4151532
Polymorphum gilvum SL003B 26A1	4136128
Verrucosispora maris AB 18 032	4120894
Streptococcus pasteurianus ATCC 43144	4105826
Geobacillus Y412MC61	4090924
Vibrio cholerae O1 2010EL 1786	4076182
Erwinia amylovora ATCC 49946	4061598
Tropheryma whipplei TW08 27	4047168
Rickettsia felis URRWXCal2	4032894
Staphylococcus carnosus TM300	4018768
Ehrlichia ruminantium Welgevonden	4004788
Porphyromonas gingivalis TDC60	3990956
Wolinella succinogenes DSM 1740	3977264
Syntrophobotulus glycolicus DSM 8271	3963714
Pseudoalteromonas atlantica T6c	3950300
Xylella fastidiosa 9a5c	3937022
Aeromonas salmonicida A449	3923876
Lactobacillus reuteri JCM 1112	3910862
Picrophilus torridus DSM 9790	3897976
Methanohalophilus mahii DSM 5219	3885216
Mycoplasma penetrans HF 2	3872582
Thermodesulfobium narugense DSM 14796	3860068
Nostoc azollae 0708	3847676
Methanothermus fervidus DSM 2088	3835404
Streptococcus suis P1 7	3823246
Blattabacterium Blattella germanica Bge	3811206
Leuconostoc citreum KM20	3799276
Herminiimonas arsenicoxydans	3787460
Thermoanaerobacter wiegelii Rt8 B1	3775752
Methanocaldococcus FS406 22	3764152
Candidatus Sulcia muelleri CARI	3752658
Pyrococcus horikoshii OT3	3741268
Geobacillus C56 T3	3729982
Streptococcus pyogenes MGAS2096	3718798
Ralstonia solanacearum CFBP2957	3707714
Desulfurobacterium thermolithotrophum DSM 11699	3696728
Ehrlichia chaffeensis Arkansas	3685840
Neisseria gonorrhoeae FA 1090	3675046
Mycoplasma genitalium G37	3664348
Helicobacter pylori J99	3653742
Parvularcula bermudensis HTCC2503	3643228
Salmonella enterica serovar Choleraesuis SC B67	3632804

Clostridium botulinum BKT015925	3622468
Methanoculleus marisnigri JR1	3612220
Lactobacillus plantarum WCFS1	3602060
Sulfurihydrogenibium azorense Az Fu1	3591984
Escherichia coli CFT073	3581992
Chlorobium tepidum TLS	3572084
Streptococcus pneumoniae AP200	3562256
Veillonella parvula DSM 2008	3552510
Methanothermobacter marburgensis Marburg	3542844
Beutenbergia cavernae DSM 12333	3533256
Haliscomenobacter hydrossis DSM 1100	3523744
Lactobacillus buchneri NRRL B 30929	3514310
Methanocorpusculum labreanum Z	3504952
Burkholderia mallei ATCC 23344	3495666
Caldicellulosiruptor lactoaceticus 6A	3486456
Mycobacterium vanbaalenii PYR 1	3477316
Staphylococcus aureus MW2	3468250
Rhodopseudomonas palustris BisA53	3459252
Geobacter M18	3450326
Francisella tularensis SCHU S4	3441466
Methylobacillus flagellatus KT	3432676
Pseudomonas fluorescens SBW25	3423952
Streptococcus mutans NN2025	3415296
Legionella pneumophila Paris	3406704
Rickettsia typhi Wilmington	3398176
Vibrio cholerae M66 2	3389712
Chromohalobacter salexigens DSM 3043	3381312
Streptococcus pneumoniae TIGR4	3372974
Mycoplasma haemofelis Langford 1	3364696
Rhodospirillum rubrum ATCC 11170	3356480
Marinobacter aquaeolei VT8	3348322
Eggerthella YY7918	3340224
Moorella thermoacetica ATCC 39073	3332186
Haemophilus parainfluenzae T3T1	3324204
Gluconacetobacter diazotrophicus PAl 5	3316280
Thermodesulfobacterium OPB45	3308412
Acidiphilium cryptum JF 5	3300600
Desulfurococcus kamchatkensis 1221n	3292844
Thermosipho melanesiensis BI429	3285140
Streptococcus oralis Uo5	3277492
Thiomonas intermedia K12	3269896
Pseudomonas syringae phaseolicola 1448A	3262354
Aeromonas hydrophila ATCC 7966	3254862
Geobacter M21	3247422
Micromonospora aurantiaca ATCC 27029	3240034
gamma proteobacterium HdN1	3232696

Bordetella parapertussis 12822	3225406
Chloroherpeton thalassium ATCC 35110	3218166
Thermotoga maritima MSB8	3210974
Salmonella enterica serovar Paratyphi B SPB7	3203832
Rhodopseudomonas palustris HaA2	3196736
Chlorobaculum parvum NCIB 8327	3189686
Salinibacter ruber DSM 13855	3182684
Chlamydia trachomatis A HAR 13	3175726
Oceanithermus profundus DSM 14977	3168816
Xanthomonas campestris vesicatoria 85 10	3161950
Dehalococcoides ethenogenes 195	3155128
Chlamydia trachomatis B Jali20 OT	3148350
Thermaerobacter marianensis DSM 12885	3141614
Methanococcus maripaludis C6	3134924
Pelotomaculum thermopropionicum SI	3128274
Thermotoga naphthophila RKU 10	3121668
Pyrobaculum calidifontis JCM 11548	3115102
Ehrlichia ruminantium Welgevonden	3108580
Staphylococcus epidermidis RP62A	3102096
Actinobacillus succinogenes 130Z	3095654
Ferrimonas balearica DSM 9799	3089252
Candidatus Sulcia muelleri DMIN	3082888
Thermovirga lienii DSM 17291	3076564
Mycobacterium bovis BCG Pasteur 1173P2	3070280
Ehrlichia canis Jake	3064032
Arcobacter butzleri RM4018	3057824
Spirochaeta smaragdinae DSM 11293	3051652
Shewanella violacea DSS12	3045518
Thermococcus barophilus MP	3039422
Candidatus Pelagibacter IMCC9063	3033360
Desulfotomaculum ruminis DSM 2154	3027336
Borrelia recurrentis A1	3021348
Bartonella quintana Toulouse	3015394
Streptococcus gordonii Challis substr CH1	3009476
Rhodococcus equi 103S	3003592
Campylobacter curvus 525 92	2997742
Sulfolobus islandicus M 14 25	2991928
Clostridium perfringens SM101	2986146
Arthrobacter aurescens TC1	2980398
Stenotrophomonas maltophilia R551 3	2974682
Candidatus Solibacter usitatus Ellin6076	2969000
Verminephrobacter eiseniae EF01 2	2963350
Hippea maritima DSM 10411	2957732
Sulfolobus islandicus Y N 15 51	2952146
Megasphaera elsdenii DSM 20460	2946592
Methanoregula boonei 6A8	2941070

Clostridium botulinum A2 Kyoto	2935578
Methanococcus maripaludis C5	2930116
Treponema succinifaciens DSM 2489	2924684
Anaplasma marginale Maries	2919284
Xanthomonas axonopodis citri 306	2913912
Streptococcus parasanguinis ATCC 15912	2908570
Oscillibacter valericigenes	2903258
Francisella TX077308	2897974
Blattabacterium Periplaneta americana BPLAN	2892720
Bifidobacterium dentium Bd1	2887494
Listeria innocua Clip11262	2882296
Bacillus amyloliquefaciens DSM 7	2877126
Bacillus licheniformis ATCC 14580	2871984
Cronobacter sakazakii ATCC BAA 894	2866868
Bacillus coagulans 36D1	2861780
Mycoplasma mycoides SC PG1	2856720
Pseudomonas aeruginosa PAO1	2851686
Dehalococcoides VS	2846678
Brucella ovis ATCC 25840	2841698
Methanobrevibacter smithii ATCC 35061	2836742
Lactobacillus acidophilus NCFM	2831814
Isoptericola variabilis 225	2826910
Salmonella enterica serovar Paratyphi C RKS4594	2822032
Bacillus anthracis CDC 684	2817178
Acaryochloris marina MBIC11017	2812350
Vibrio cholerae O1 biovar El Tor N16961	2807548
Bacillus cereus ATCC 14579	2802768
Pedobacter heparinus DSM 2366	2798014
Pelagibacterium halotolerans B2	2793284
Sideroxydans lithotrophicus ES 1	2788578
Psychrobacter PRwf 1	2783894
Thermus thermophilus HB8	2779236
Alicyclobacillus acidocaldarius DSM 446	2774600
Candidatus Blochmannia vafer BVAF	2769986
Burkholderia mallei NCTC 10229	2765396
Streptomyces coelicolor A3 2	2760830
Acetobacter pasteurianus IFO 3283 01	2756284
Metallosphaera sedula DSM 5348	2751762
Natronomonas pharaonis DSM 2160	2747262
Desulfococcus oleovorans Hxd3	2742784
Ketogulonicigenium vulgare Y25	2738328
Cyanothece PCC 8801	2733894
Staphylococcus aureus JH9	2729480
Spirochaeta thermophila DSM 6192	2725090
Mycoplasma agalactiae PG2	2720718
Vibrio fischeri MJ11	2716368

Thermotoga lettingae TMO	2712040
Marinithermus hydrothermalis DSM 14884	2707732
Dickeya zeae Ech1591	2703444
Clavibacter michiganensis NCPPB 382	2699176
Bacillus atrophaeus 1942	2694928
Yersinia pseudotuberculosis IP 32953	2690702
Comamonas testosteroni CNB 2	2686494
Mycobacterium tuberculosis H37Ra	2682306
Escherichia coli O157 H7 EDL933	2678138
Pseudomonas putida KT2440	2673990
Rothia dentocariosa ATCC 17931	2669860
Staphylococcus epidermidis ATCC 12228	2665748
Thermobifida fusca YX	2661656
Campylobacter jejuni doylei 269 97	2657584
Robiginitalea biformata HTCC2501	2653530
Photorhabdus luminescens laumondii TTO1	2649494
Rahnella Y9602	2645476
Mannheimia succiniciproducens MBEL55E	2641478
Thermotoga neapolitana DSM 4359	2637496
Porphyromonas gingivalis ATCC 33277	2633534
Streptomyces cattleya NRRL 8057	2629588
Rhodopseudomonas palustris BisB5	2625660
Rhodopseudomonas palustris BisB18	2621750
Rhodococcus jostii RHA1	2617858
Pelobacter propionicus DSM 2379	2613982
Kytococcus sedentarius DSM 20547	2610124
Flexistipes sinusarabici DSM 4947	2606282
Wigglesworthia glossinidia endosymbiont of Glossina brevipalpis	2602458
Oligotropha carboxidovorans OM5	2598650
Helicobacter pylori B8	2594860
cyanobacterium UCYN A	2591086
Thermoanaerobacter X513	2587328
Yersinia pestis KIM 10	2583586
Weeksella virosa DSM 16922	2579860
Buchnera aphidicola Cc Cinara cedri	2576150
Burkholderia pseudomallei 668	2572458
Salmonella enterica serovar Typhi CT18	2568780
Caulobacter K31	2565118
Desulfovibrio magneticus RS 1	2561472
Thermotoga petrophila RKU 1	2557840
Vibrio parahaemolyticus RIMD 2210633	2554226
Pseudomonas stutzeri ATCC 17588 LMG 11199	2550626
Thermococcus gammatolerans EJ3	2547040
Micavibrio aeruginosavorus ARL 13	2543470
Rickettsia prowazekii Madrid E	2539916
Sulfurihydrogenibium YO3AOP1	2536376

Desulfobacca acetoxidans DSM 11109	2532850
Teredinibacter turnerae T7901	2529340
Pseudomonas brassicacearum NFM421	2525844
Escherichia coli S88	2522362
Pectobacterium wasabiae WPP163	2518896
Haloarcula hispanica ATCC 33960	2515442
Nitrosomonas eutropha C91	2512004
Staphylothermus marinus F1	2508580
Thermanaerovibrio acidaminovorans DSM 6589	2505168
Methylomonas methanica MC09	2501772
Thermosediminibacter oceani DSM 16646	2498388
Bacillus selenitireducens MLS10	2495020
Desulfobulbus propionicus DSM 2032	2491664
Desulfotomaculum acetoxidans DSM 771	2488322
Prochlorococcus marinus MIT 9301	2484992
Shigella boydii Sb227	2481676
Borrelia garinii PBi	2478374
Mesorhizobium ciceri biovar biserrulae WSM1271	2475086
Lysinibacillus sphaericus C3 41	2471810
Enterobacter aerogenes KCTC 2190	2468546
Burkholderia phytofirmans PsJN	2465296
Chitinophaga pinensis DSM 2588	2462058
Serratia AS12	2458834
Prochlorococcus marinus MIT 9312	2455622
Salinispora tropica CNB 440	2452422
Ammonifex degensii KC4	2449236
Prochlorococcus marinus NATL2A	2446060
Rhizobium leguminosarum bv trifolii WSM2304	2442898
Paenibacillus Y412MC10	2439748
Enterobacter asburiae LF7a	2436610
Bacteroides salanitronis DSM 18170	2433484
Rhizobium NGR234	2430370
Listeria monocytogenes EGD e	2427268
Burkholderia cenocepacia MC0 3	2424178
Caldivirga maquilingensis IC 167	2421100
Croceibacter atlanticus HTCC2559	2418034
Agrobacterium tumefaciens C58	2414978
Clostridium difficile 630	2411936
Lactobacillus helveticus DPC 4571	2408904
Listeria monocytogenes 08 5923	2405882
Rhizobium leguminosarum bv trifolii WSM1325	2402874
Thermovibrio ammonificans HB 1	2399876
Geobacillus thermodenitrificans NG80 2	2396888
Blattabacterium Mastotermes darwiniensis MADAR	2393912
Shewanella denitrificans OS217	2390948
Mycobacterium avium 104	2387994

Brevundimonas subvibrioides ATCC 15264	2385052
Streptococcus salivarius CCHSS3	2382120
Corynebacterium glutamicum ATCC 13032	2379200
Methanosalsum zhilinae DSM 4017	2376288
Burkholderia pseudomallei 1710b	2373390
Pyrobaculum arsenaticum DSM 13514	2370500
Bacillus anthracis Ames	2367622
Klebsiella pneumoniae 342	2364754
Sulfuricurvum kujiense DSM 16994	2361896
Neisseria meningitidis 053442	2359048
Pyrococcus NA2	2356212
Candidatus Hamiltonella defensa 5AT Acyrthosiphon pisum	2353384
Runella slithyformis DSM 19594	2350568
Mycoplasma hyopneumoniae 232	2347760
Rhodoferax ferrireducens T118	2344964
Bordetella pertussis Tohama I	2342178
Thermoanaerobacter tengcongensis MB4	2339400
Chlamydia trachomatis L2b UCH 1 proctitis	2336634
Heliobacterium modesticaldum Ice1	2333876
Ureaplasma parvum serovar 3 ATCC 700970	2331130
Sphingobium japonicum UT26S	2328392
Ralstonia eutropha JMP134	2325664
Treponema paraluiscuniculi Cuniculi A	2322946
Streptococcus pneumoniae 70585	2320236
Nitratifractor salsuginis DSM 16511	2317536
Clostridium novyi NT	2314846
Xenorhabdus nematophila ATCC 19061	2312166
Escherichia coli O157 H7 Sakai	2309494
Thermobaculum terrenum ATCC BAA 798	2306832
Frankia CcI3	2304180
Methanosaeta thermophila PT	2301536
Parvibaculum lavamentivorans DS 1	2298900
Methanobacterium SWAN 1	2296274
Buchnera aphidicola 5A Acyrthosiphon pisum	2293658
Caldicellulosiruptor owensensis OL	2291050
Frankia EAN1pec	2288450
Streptococcus sanguinis SK36	2285860
Syntrophothermus lipocalidus DSM 12680	2283280
Bacillus pseudofirmus OF4	2280706
Pirellula staleyi DSM 6068	2278142
Staphylococcus aureus Mu3	2275588
Borrelia turicatae 91E135	2273040
Frankia symbiont of Datisca glomerata	2270502
Methanothermococcus okinawensis IH1	2267972
Wolbachia endosymbiont of Culex quinquefasciatus Pel	2265450
Actinobacillus pleuropneumoniae serovar 7 AP76	2262938

Dehalococcoides CBDB1	2260434
Haemophilus influenzae F3047	2257936
Treponema azotonutricium ZAS 9	2255448
Roseobacter litoralis Och 149	2252968
Shigella sonnei Ss046	2250498
Francisella novicida U112	2248034
Salinibacter ruber M8	2245578
Bacillus amyloliquefaciens FZB42	2243130
Brucella melitensis ATCC 23457	2240690
Thermosphaera aggregans DSM 11486	2238260
Bacillus cereus E33L	2235836
Streptococcus suis ST3	2233420
Legionella pneumophila Lens	2231012
Synechococcus RCC307	2228612
Klebsiella variicola At 22	2226220
Lactobacillus rhamnosus GG	2223834
Salmonella enterica serovar Typhimurium LT2	2221458
Streptobacillus moniliformis DSM 12112	2219088
Escherichia coli E24377A	2216726
Rhizobium etli CIAT 652	2214372
Stackebrandtia nassauensis DSM 44728	2212024
Streptococcus pyogenes MGAS315	2209684
Erysipelothrix rhusiopathiae Fujisawa	2207352
Mycobacterium bovis AF2122 97	2205028
Aster yellows witches broom phytoplasma AYWB	2202710
Mycoplasma pulmonis UAB CTIP	2200400
Roseiflexus RS 1	2198098
Streptococcus dysgalactiae equisimilis GGS 124	2195802
Lactobacillus casei ATCC 334	2193514
Pusillimonas T7 7	2191232
Clavibacter michiganensis sepedonicus	2188958
Staphylococcus haemolyticus JCSC1435	2186690
Bacillus cereus 03BB102	2184430
Escherichia fergusonii ATCC 35469	2182178
Erwinia pyrifoliae Ep1 96	2179932
Halopiger xanaduensis SH 6	2177692
Rickettsia japonica YH	2175460
Psychrobacter arcticus 273 4	2173234
Desulfurivibrio alkaliphilus AHT2	2171016
Novosphingobium PP1Y	2168804
Ralstonia pickettii 12D	2166598
Streptococcus pneumoniae CGSP14	2164400
Shewanella loihica PV 4	2162208
Helicobacter pylori G27	2160022
Synechococcus CC9902	2157844
Oceanobacillus iheyensis HTE831	2155672

Idiomarina loihiensis L2TR	2153506
Candidatus Chloracidobacterium thermophilum B	2151348
Lacinutrix 5H 3 7 4	2149196
Streptococcus equi zooepidemicus	2147050
Bifidobacterium adolescentis ATCC 15703	2144910
Aeropyrum pernix K1	2142776
Legionella longbeachae NSW150	2140650
Xanthomonas campestris 8004	2138530
Streptococcus pneumoniae ATCC 700669	2136416
Helicobacter pylori HPAG1	2134308
Mycoplasma leachii PG50	2132206
Petrotoga mobilis SJ95	2130110
Mycobacterium smegmatis MC2 155	2128020
Legionella pneumophila Corby	2125938
Paludibacter propionicigenes WB4	2123860
Metallosphaera cuprina Ar 4	2121790
Xylella fastidiosa M23	2119724
Magnetospirillum magneticum AMB 1	2117666
Haliangium ochraceum DSM 14365	2115612
Bacillus licheniformis ATCC 14580	2113566
Acidilobus saccharovorans 345 15	2111524
Vibrio anguillarum 775	2109488
Coprothermobacter proteolyticus DSM 5265	2107460
Prochlorococcus marinus CCMP1375	2105436
Staphylococcus aureus JH1	2103418
Ferroglobus placidus DSM 10642	2101406
Acidovorax JS42	2099400
Dehalococcoides BAV1	2097400
Pseudomonas fulva 12 X	2095406
Methanocaldococcus fervens AG86	2093416
Geobacter uraniireducens Rf4	2091432
Vibrio Ex25	2089456
Clostridium lentocellum DSM 5427	2087484
Streptococcus mitis B6	2085516
Lactobacillus reuteri DSM 20016	2083556
Streptococcus pseudopneumoniae IS7493	2081600
Salmonella enterica serovar Paratyphi A AKU 12601	2079650
Leptotrichia buccalis C 1013 b	2077706
Halobacterium NRC 1	2075766
Corynebacterium efficiens YS 314	2073832
Alkaliphilus oremlandii OhILAs	2071904
Lactobacillus plantarum ST III	2069982
Propionibacterium freudenreichii shermanii CIRM BIA1	2068064
Borrelia burgdorferi ZS7	2066152
Borrelia duttonii Ly	2064246
Escherichia coli O111 H 11128	2062344

Carboxydothermus hydrogenoformans Z 2901	2060446
Nitrosomonas Is79A3	2058556
Mycobacterium JLS	2056670
Frankia alni ACN14a	2054788
Thermoplasma volcanium GSS1	2052914
Methylobacterium extorquens PA1	2051042
Coriobacterium glomerans PW2	2049178
Prochlorococcus marinus NATL1A	2047316
Halorhabdus utahensis DSM 12940	2045462
Prevotella denticola F0289	2043612
Hahella chejuensis KCTC 2396	2041766
Candidatus Ruthia magnifica Cm Calyptogena magnifica	2039926
Muricauda ruestringensis DSM 13258	2038090
Hirschia baltica ATCC 49814	2036260
Bacillus cereus Q1	2034434
Staphylococcus aureus NCTC 8325	2032614
Symbiobacterium thermophilum IAM 14863	2030798
Treponema brennaborense DSM 12168	2028988
Serratia AS9	2027182
Klebsiella pneumoniae MGH 78578	2025380
Nitrobacter winogradskyi Nb 255	2023584
Polynucleobacter necessarius STIR1	2021792
Acidobacterium MP5ACTX9	2020006
Synechococcus elongatus PCC 6301	2018224
Xanthomonas axonopodis citrumelo F1	2016446
Caulobacter segnis ATCC 21756	2014674
Desulfotomaculum kuznetsovii DSM 6115	2012906
Fluviicola taffensis DSM 16823	2011142
Lactobacillus salivarius UCC118	2009384
Rickettsia bellii OSU 85 389	2007630
Rickettsia rickettsii Sheila Smith	2005880
Rhodopseudomonas palustris CGA009	2004134
Desulfomicrobium baculatum DSM 4028	2002394
Haemophilus influenzae Rd KW20	2000658
Eubacterium eligens ATCC 27750	1998928
Geobacter sulfurreducens PCA	1997200
Methylocella silvestris BL2	1995478
Escherichia coli IAI1	1993760
Ignicoccus hospitalis KIN4 I	1992046
Delftia Cs1 4	1990336
Micromonospora L5	1988632
Methylibium petroleiphilum PM1	1986932
Terriglobus saanensis SP1PR4	1985236
Xanthomonas campestris B100	1983544
Lactobacillus rhamnosus Lc 705	1981856
Chlamydophila abortus S26 3	1980174

Pantoea At 9b	1978494
Geobacter lovleyi SZ	1976820
Rhodobacter capsulatus SB 1003	1975150
Bifidobacterium longum JCM 1217	1973484
Sodalis glossinidius morsitans	1971822
Bifidobacterium longum DJO10A	1970164
Leuconostoc gasicomitatum LMG 18811	1968510
Streptococcus pyogenes MGAS6180	1966862
Staphylococcus aureus N315	1965216
Leptospira interrogans serovar Copenhageni Fiocruz L1 130	1963574
Ramlibacter tataouinensis TTB310	1961938
Variovorax paradoxus EPS	1960306
Streptococcus pyogenes M1 GAS	1958676
Staphylococcus aureus Newman	1957052
Methylobacterium populi BJ001	1955430
Sulfurimonas autotrophica DSM 16294	1953814
Salmonella enterica serovar Schwarzengrund CVM19633	1952202
Caldicellulosiruptor saccharolyticus DSM 8903	1950592
Brucella melitensis biovar Abortus 2308	1948988
Denitrovibrio acetiphilus DSM 12809	1947386
Leptospira interrogans serovar Lai 56601	1945790
Sulfobacillus acidophilus TPY	1944198
Burkholderia gladioli BSR3	1942608
Oligotropha carboxidovorans OM5	1941022
Brucella suis ATCC 23445	1939442
Thermococcus onnurineus NA1	1937864
Vibrio cholerae MJ 1236	1936290
Pantoea vagans C9 1	1934720
Methanotorris igneus Kol 5	1933154
Yersinia pestis CO92	1931592
Anaeromyxobacter dehalogenans 2CP C	1930034
Burkholderia mallei NCTC 10247	1928480
Enterococcus faecalis V583	1926928
Xanthomonas campestris ATCC 33913	1925382
Pseudomonas mendocina ymp	1923838
Acidaminococcus intestini RyC MR95	1922298
Klebsiella pneumoniae NTUH K2044	1920762
Bacillus anthracis Ames Ancestor	1919230
Methylovorus MP688	1917702
Methanocaldococcus jannaschii DSM 2661	1916176
Rickettsia peacockii Rustic	1914656
Geodermatophilus obscurus DSM 43160	1913138
Methylobacterium nodulans ORS 2060	1911624
Aeromonas veronii B565	1910112
Clostridium difficile R20291	1908606
Candidatus Azobacteroides pseudotrichonymphae genomovar CFP2	1907102

Ralstonia solanacearum GMI1000	1905602
Nitratiruptor SB155 2	1904106
Exiguobacterium sibiricum 255 15	1902614
Buchnera aphidicola Cinara tujafilina	1901124
Thermus scotoductus SA 01	1899638
Lactobacillus sakei 23K	1898156
Streptococcus pyogenes MGAS10270	1896676
Sulfurimonas denitrificans DSM 1251	1895202
Thermoanaerobacter pseudethanolicus ATCC 33223	1893730
Anaeromyxobacter dehalogenans 2CP 1	1892260
Shewanella oneidensis MR 1	1890796
Ehrlichia ruminantium Gardel	1889334
Pseudomonas syringae tomato DC3000	1887876
Jonesia denitrificans DSM 20603	1886420
Staphylococcus aureus USA300 TCH1516	1884968
Vibrio fischeri ES114	1883520
Shewanella baltica OS195	1882076
Burkholderia CCGE1003	1880634
Mycobacterium avium paratuberculosis K 10	1879196
Desulfohalobium retbaense DSM 5692	1877760
Acholeplasma laidlawii PG 8A	1876328
Neorickettsia risticii Illinois	1874900
Moraxella catarrhalis RH4	1873476
Akkermansia muciniphila ATCC BAA 835	1872054
Streptococcus mutans UA159	1870634
Escherichia coli BL21 Gold DE3 pLysS AG	1869218
Roseobacter denitrificans OCh 114	1867806
Conexibacter woesei DSM 14684	1866398
Edwardsiella ictaluri 93 146	1864992
Sinorhizobium medicae WSM419	1863588
Deinococcus proteolyticus MRP	1862190
Methanococcus maripaludis C7	1860792
Cytophaga hutchinsonii ATCC 33406	1859400
Marivirga tractuosa DSM 4126	1858010
Xylanimonas cellulosilytica DSM 15894	1856622
Streptococcus pneumoniae TCH8431 19A	1855238
Burkholderia phymatum STM815	1853858
Leuconostoc kimchii IMSNU 11154	1852480
Bacillus cereus G9842	1851104
Roseiflexus castenholzii DSM 13941	1849732
Desulfurococcus mucosus DSM 2162	1848364
Brucella suis 1330	1846998
Pseudomonas syringae B728a	1845636
Clostridium cellulovorans 743B	1844276
Methanosphaera stadtmanae DSM 3091	1842920
Thermodesulfovibrio yellowstonii DSM 11347	1841566

Yersinia pestis Antiqua	1840216
Kocuria rhizophila DC2201	1838868
Bacteroides vulgatus ATCC 8482	1837524
Baumannia cicadellinicola Hc Homalodisca coagulata	1836182
Truepera radiovictrix DSM 17093	1834842
Burkholderia JV3	1833506
Pseudoalteromonas SM9913	1832174
Dickeya dadantii Ech586	1830844
Clostridium SY8519	1829516
Yersinia pseudotuberculosis YPIII	1828192
Candidatus Blochmannia floridanus	1826870
Maricaulis maris MCS10	1825552
Yersinia pseudotuberculosis PB1	1824236
Rickettsia rickettsii Iowa	1822924
Ureaplasma urealyticum serovar 10 ATCC 33699	1821614
Neisseria gonorrhoeae NCCP11945	1820306
Francisella tularensis FSC198	1819002
Zymomonas mobilis ZM4	1817700
Escherichia coli B REL606	1816402
Staphylococcus aureus MRSA252	1815106
Tepidanaerobacter Re1	1813812
Synechococcus CC9311	1812522
Edwardsiella tarda EIB202	1811234
Lactobacillus johnsonii FI9785	1809948
Campylobacter hominis ATCC BAA 381	1808666
Escherichia coli APEC O1	1807388
Phenylobacterium zucineum HLK1	1806110
Bifidobacterium animalis lactis Bl 04	1804836
Mycoplasma pneumoniae M129	1803564
Francisella philomiragia ATCC 25017	1802296
Shewanella baltica OS185	1801030
Ignisphaera aggregans DSM 17230	1799766
Staphylococcus aureus ED98	1798506
Mycoplasma bovis PG45	1797248
Haloquadratum walsbyi DSM 16790	1795992
Yersinia pestis Pestoides F	1794740
Elusimicrobium minutum Pei191	1793488
Staphylothermus hellenicus DSM 12710	1792242
Rhodobacter sphaeroides 2 4 1	1790996
Taylorella asinigenitalis MCE3	1789754
Halothiobacillus neapolitanus c2	1788514
Propionibacterium acnes KPA171202	1787276
Xylella fastidiosa Temecula1	1786042
Lactobacillus casei Zhang	1784810
Desulfitobacterium hafniense Y51	1783580
Cyanothece ATCC 51142	1782352

Buchnera aphidicola Tuc7 Acyrthosiphon pisum	1781128
Shewanella sediminis HAW EB3	1779906
Polynucleobacter necessarius asymbioticus QLW P1DMWA 1	1778686
Borrelia hermsii DAH	1777470
Bifidobacterium animalis lactis AD011	1776256
Bacillus subtilis BSn5	1775044
Campylobacter jejuni NCTC 11168	1773834
Burkholderia ambifaria AMMD	1772626
Lactococcus lactis cremoris MG1363	1771422
Ralstonia solanacearum PSI07	1770220
Arcobacter nitrofigilis DSM 7299	1769020
Caulobacter crescentus CB15	1767822
Bdellovibrio bacteriovorus HD100	1766628
Pedobacter saltans DSM 12145	1765436
Marinomonas MWYL1	1764246
Bradyrhizobium BTAi1	1763058
Burkholderia ambifaria MC40 6	1761872
Burkholderia multivorans ATCC 17616	1760690
Collimonas fungivorans Ter331	1759508
Nanoarchaeum equitans Kin4 M	1758330
Butyrivibrio proteoclasticus B316	1757156
Streptomyces SirexAA E	1755982
Shigella flexneri 2a 301	1754810
Burkholderia glumae BGR1	1753642
Chlamydia trachomatis L2c	1752476
Thermosynechococcus elongatus BP 1	1751312
Desulfotalea psychrophila LSv54	1750150
Burkholderia vietnamiensis G4	1748990
Gardnerella vaginalis 409 05	1747834
Candidatus Midichloria mitochondrii IricVA	1746678
Borrelia bissettii DN127	1745526
Mycobacterium africanum GM041182	1744376
Sulfolobus tokodaii 7	1743228
Sulfolobus islandicus M 16 27	1742082
Methanococcus maripaludis X1	1740938
Sorangium cellulosum So ce 56	1739798
Zobellia galactanivorans	1738658
Methylobacterium chloromethanicum CM4	1737522
Desulfovibrio alaskensis G20	1736386
Thermoproteus tenax Kra 1	1735254
Bacillus subtilis 168	1734124
Helicobacter pylori P12	1732996
Streptococcus suis 05ZYH33	1731870
Escherichia coli ED1a	1730748
Thermoanaerobacter brockii finnii Ako 1	1729626
Arthrobacter arilaitensis Re117	1728506

Methanopyrus kandleri AV19	1727390
Bifidobacterium bifidum S17	1726276
Escherichia coli UMN026	1725162
Bacillus thuringiensis BMB171	1724052
Burkholderia CCGE1002	1722944
Ruegeria pomeroyi DSS 3	1721838
Pyrobaculum aerophilum IM2	1720734
Asticcacaulis excentricus CB 48	1719632
Neisseria meningitidis alpha14	1718532
Thermofilum pendens Hrk 5	1717434
Jannaschia CCS1	1716338
Francisella tularensis holarctica FTNF002 00	1715244
Shigella dysenteriae Sd197	1714154
Streptococcus thermophilus LMD 9	1713064
Methanococcus vannielii SB	1711976
Shewanella baltica OS155	1710892
Propionibacterium acnes SK137	1709808
Bacillus subtilis spizizenii W23	1708726
Achromobacter xylosoxidans A8	1707648
Ochrobactrum anthropi ATCC 49188	1706570
Rhodomicrobium vannielii ATCC 17100	1705496
Cenarchaeum symbiosum A	1704422
Halomonas elongata DSM 2581	1703352
Burkholderia pseudomallei K96243	1702282
Bartonella tribocorum CIP 105476	1701216
Haemophilus parasuis SH0165	1700152
Rickettsia akari Hartford	1699088
Streptomyces violaceusniger Tu 4113	1698028
Candidatus Blochmannia pennsylvanicus BPEN	1696968
Halogeometricum borinquense DSM 11551	1695912
Pectobacterium carotovorum PC1	1694856
Parabacteroides distasonis ATCC 8503	1693804
Acidithiobacillus ferrooxidans ATCC 53993	1692752
Desulfotomaculum reducens MI 1	1691702
Caldicellulosiruptor kristjanssonii 177R1B	1690656
Photorhabdus asymbiotica ATCC 43949	1689610
Flavobacteriales bacterium HTCC2170	1688568
Shewanella halifaxensis HAW EB4	1687526
Planctomyces brasiliensis DSM 5305	1686486
Mesoplasma florum L1	1685448
Clostridium phytofermentans ISDg	1684414
Streptosporangium roseum DSM 43021	1683380
Agrobacterium H13 3	1682348
Shewanella MR 4	1681318
Acinetobacter baumannii AB307 0294	1680290
Shewanella W3 18 1	1679264

Spirochaeta coccoides DSM 17374	1678240
Pyrococcus abyssi GE5	1677218
Escherichia coli UTI89	1676196
Francisella tularensis holarctica OSU18	1675178
Burkholderia cenocepacia AU 1054	1674162
Halobacterium salinarum R1	1673146
Kosmotoga olearia TBF 19 5 1	1672134
Streptococcus agalactiae A909	1671122
Nakamurella multipartita DSM 44233	1670112
Escherichia coli 55989	1669104
Clostridium botulinum F Langeland	1668100
Corynebacterium glutamicum ATCC 13032	1667096
Acidaminococcus fermentans DSM 20731	1666092
Aliivibrio salmonicida LFI1238	1665092
Cyanothece PCC 7822	1664094
Chlamydophila caviae GPIC	1663098
Cellulophaga lytica DSM 7489	1662102
Mycoplasma suis KI3806	1661110
Thermoproteus uzoniensis 768 20	1660118
Sphaerobacter thermophilus DSM 20745	1659128
Methylobacterium extorquens AM1	1658140
Saccharophagus degradans 2 40	1657154
Orientia tsutsugamushi Boryong	1656170
Escherichia coli O127 H6 E2348 69	1655188
Bifidobacterium longum infantis 157F	1654206
Kitasatospora setae KM 6054	1653228
Staphylococcus pseudintermedius HKU10 03	1652250
Campylobacter lari RM2100	1651274
Magnetococcus MC 1	1650300
Desulfovibrio salexigens DSM 2638	1649328
Treponema primitia ZAS 2	1648358
Bacteroides fragilis NCTC 9343	1647388
Deinococcus deserti VCD115	1646422
Aggregatibacter aphrophilus NJ8700	1645456
Colwellia psychrerythraea 34H	1644492
Thioalkalivibrio sulfidophilus HL EbGr7	1643530
Acidothermus cellulolyticus 11B	1642570
Rhodopirellula baltica SH 1	1641612
Methylotenera mobilis JLW8	1640654
Pseudomonas fluorescens Pf0 1	1639700
Arthrobacter chlorophenolicus A6	1638746
Thermotoga thermarum DSM 5069	1637794
Finegoldia magna ATCC 29328	1636844
Bacillus cereus biovar anthracis CI	1635896
Rickettsia canadensis McKiel	1634948
Chlamydophila pecorum E58	1634002

Mycobacterium tuberculosis H37Rv	1633060
Proteus mirabilis HI4320	1632118
Cupriavidus metallidurans CH34	1631176
Clostridium botulinum B1 Okra	1630238
Mycoplasma bovis Hubei 1	1629300
Enterobacter cloacae SCF1	1628366
Wolbachia wRi	1627432
Myxococcus xanthus DK 1622	1626498
Streptococcus pyogenes NZ131	1625568
Shewanella ANA 3	1624638
Clostridium beijerinckii NCIMB 8052	1623712
Candidatus Amoebophilus asiaticus 5a2	1622786
Burkholderia cenocepacia J2315	1621860
Weissella koreensis KACC 15510	1620938
Ralstonia pickettii 12J	1620016
Staphylococcus saprophyticus ATCC 15305	1619098
Thermococcus sibiricus MM 739	1618180
Streptococcus pyogenes MGAS9429	1617262
Planctomyces limnophilus DSM 3776	1616348
Cyclobacterium marinum DSM 745	1615434
Methanobacterium AL 21	1614522
Odoribacter splanchnicus DSM 20712	1613612
Methanococcus voltae A3	1612704
Chlamydia trachomatis 434 Bu	1611796
Mycoplasma hyopneumoniae 7448	1610890
Novosphingobium aromaticivorans DSM 12444	1609986
Rhodothermus marinus DSM 4252	1609084
Burkholderia mallei SAVP1	1608182
Mycoplasma hominis ATCC 23114	1607282
Xanthobacter autotrophicus Py2	1606384
Methylococcus capsulatus Bath	1605488
Mycobacterium Spyr1	1604592
Marinomonas posidonica IVIA Po 181	1603698
Chlamydophila felis Fe C 56	1602806
Candidatus Protochlamydia amoebophila UWE25	1601916
Pseudoxanthomonas spadix BD a59	1601026
Saccharomonospora viridis DSM 43017	1600138
Streptococcus pneumoniae R6	1599252
Chlamydophila psittaci 6BC	1598368
Gluconacetobacter xylinus NBRC 3288	1597484
Salmonella enterica serovar Paratyphi A ATCC 9150	1596602
Roseburia hominis A2 183	1595722
Burkholderia rhizoxinica HKI 454	1594844
Bacillus cereus ATCC 10987	1593966
Yersinia pseudotuberculosis IP 31758	1593090
Mycobacterium leprae Br4923	1592214

Lactobacillus casei BL23	1591342
Lactobacillus delbrueckii bulgaricus ND02	1590470
Brucella abortus S19	1589600
Synechococcus JA 3 3Ab	1588730
Shewanella putrefaciens CN 32	1587864
Janthinobacterium Marseille	1586998
Methanococcus aeolicus Nankai 3	1586132
Glaciecola 4H 3 7 YE 5	1585270
Rickettsia conorii Malish 7	1584408
Pseudoalteromonas haloplanktis TAC125	1583548
Escherichia coli ATCC 8739	1582688
Leadbetterella byssophila DSM 17132	1581830
Candidatus Carsonella ruddii	1580974
Chromobacterium violaceum ATCC 12472	1580120
Mycobacterium MCS	1579266
Simkania negevensis Z	1578414
Vulcanisaeta moutnovskia 768 28	1577564
Salinispora arenicola CNS 205	1576714
Dictyoglomus thermophilum H 6 12	1575866
Vibrio splendidus LGP32	1575020
Leptospira biflexa serovar Patoc Patoc 1 Ames	1574174
Cyanothece PCC 7425	1573330
Lactobacillus amylovorus GRL 1112	1572488
Taylorella equigenitalis MCE9	1571648
Lactobacillus ruminis ATCC 27782	1570808
Clostridium difficile CD196	1569970
Alicycliphilus denitrificans BC	1569132
Acidianus hospitalis W1	1568296
Fervidobacterium nodosum Rt17 B1	1567462
Mycobacterium leprae TN	1566628
Tropheryma whipplei Twist	1565796
Paracoccus denitrificans PD1222	1564966
Desulfovibrio vulgaris Miyazaki F	1564138
Thermosipho africanus TCF52B	1563310
Desulfurispirillum indicum S5	1562484
Mycoplasma suis Illinois	1561658
Yersinia pestis Nepal516	1560834
Cellulomonas fimi ATCC 484	1560012
Xanthomonas oryzae KACC10331	1559190
Vibrio cholerae O395	1558370
Rickettsia africae ESF 5	1557552
Bacillus weihenstephanensis KBAB4	1556734
Variovorax paradoxus S110	1555918
Xanthomonas oryzae PXO99A	1555104
Bacteroides fragilis YCH46	1554290
Methanoplanus petrolearius DSM 11571	1553478

Francisella tularensis mediasiatica FSC147	1552666
Bacillus pumilus SAFR 032	1551856
Escherichia coli O103 H2 12009	1551048
Buchnera aphidicola Sg Schizaphis graminum	1550240
Synechococcus JA 2 3B a 2 13	1549434
Shigella flexneri 2a 2457T	1548630
Exiguobacterium AT1b	1547826
Thermomonospora curvata DSM 43183	1547024
Lactococcus lactis cremoris SK11	1546224
Corynebacterium aurimucosum ATCC 700975	1545424
Mycoplasma hyorhinis HUB 1	1544626
Meiothermus ruber DSM 1279	1543828
Campylobacter jejuni 81 176	1543032
Caldicellulosiruptor hydrothermalis 108	1542238
Streptococcus pneumoniae Taiwan19F 14	1541444
Escherichia coli 536	1540652
Deinococcus maricopensis DSM 21211	1539860
Streptococcus suis BM407	1539070
Bacillus thuringiensis serovar konkukian 97 27	1538282
Meiothermus silvanus DSM 9946	1537494
Streptococcus gallolyticus UCN34	1536708
Salmonella enterica serovar Newport SL254	1535924
Gemmatimonas aurantiaca T 27	1535140
Thiomicrospira crunogena XCL 2	1534356
Eubacterium rectale ATCC 33656	1533576
Thermincola potens JR	1532796
Vulcanisaeta distributa DSM 14429	1532016
Pseudomonas putida GB 1	1531238
Methylacidiphilum infernorum V4	1530462
Streptococcus equi zooepidemicus MGCS10565	1529686
Coxiella burnetii CbuG Q212	1528912
Anaplasma phagocytophilum HZ	1528138
Chlorobium luteolum DSM 273	1527366
Shewanella MR 7	1526596
Methylomicrobium alcaliphilum	1525826
Desulfobacterium autotrophicum HRM2	1525058
Candidatus Accumulibacter phosphatis clade IIA UW 1	1524290
Shewanella piezotolerans WP3	1523524
Arthrobacter phenanthrenivorans Sphe3	1522760
Acidithiobacillus caldus SM 1	1521996
Azospirillum B510	1521232
Pelobacter carbinolicus DSM 2380	1520470
Bacillus halodurans C 125	1519710

Supplementary Table 3: Overlay data on metagenome assembly of 124 gut microbiome samples. For the 124 samples, read length was 44 and/or 75 nucleotides and the 3 insert sizes were 135, 200, 400 nucleotides [1]. Of the 124, 122 samples were assembled and profiled with 32 processor cores (4 machines with 8 cores each). MH0014 was processed with 40 processor cores while MH0012 was processed with 48 processor cores. The average computing time was 4:55. The memory usage per core was between 500 mega bytes and 2 giga-bytes. IBD: inflammatory bowel disease.

Sample	IBD	Number of sequences	Number of contigs (>= 100 nt)	Total length (nt)	Longuest contig	Computing time (h:m)
MH0001	N	45018302	42191	11611390	46485	3:06
MH0002	N	46575183	419154	106996731	159840	5:26
MH0003	N	50388076	465208	138978682	91018	5:55
MH0004	N	40299958	64770	16647746	52003	3:56
MH0005	N	39412204	34900	12013030	55829	3:09
MH0006	N	161222460	962511	256948721	201855	10:06
MH0007	N	36961474	67910	10481227	10785	3:17
MH0008	N	38158736	103056	23347007	12471	2:58
MH0009	N	58458876	494967	130258663	98955	6:59
MH0010	N	39172670	83327	24422433	37338	3:06
MH0011	N	58513980	587385	150689295	173046	5:31
MH0012	N	186073978	935974	302188253	189292	8:13
MH0013	N	40051756	86723	16553992	93150	2:56
MH0014	N	56313876	502034	149796789	152916	5:09
MH0015	N	33016500	68071	9805671	14237	2:53
MH0016	N	54400556	476090	137428961	138300	5:53
MH0017	N	36156056	71786	14076110	16802	2:48
MH0018	N	36641824	41408	13463875	48221	2:52
MH0019	N	38859574	116745	21449250	26646	2:56
MH0020	N	45832716	307480	106168610	118057	4:34
MH0021	N	26262536	356138	81558840	91507	3:55
MH0022	N	37116608	51178	12419331	18261	3:12
MH0023	N	37785104	59630	14932565	42874	4:18
MH0024	N	54920805	296450	76043981	112472	4:33
MH0025	N	55611424	510306	129194470	75609	6:23
MH0026	N	37486710	250505	77495095	119128	4:04
MH0027	N	32379416	37674	5754439	8063	2:47
MH0028	N	55157588	417252	117581809	90407	4:57
MH0030	N	51646716	271761	75688147	98479	4:29
MH0031	N	55568300	374438	88255563	121543	5:09
MH0032	N	50126050	272791	58557518	62760	4:11
MH0033	N	44998898	440516	118775864	84397	5:25
MH0034	N	37402104	200160	43879052	48844	4:40
MH0035	N	49322484	449032	125591528	112441	6:06
MH0036	N	47708645	395710	108943341	67476	5:20
MH0037	N	42037386	329841	91003047	185137	4:30

MH0038	N	45491688	346958	99103988	126521	4:47
MH0039	N	42719254	480975	115740776	111761	4:47
MH0040	N	44251866	519549	134581198	60369	5:54
MH0041	N	44395542	370395	108830338	172308	4:41
MH0042	N	43441776	489225	105136225	75499	5:25
MH0043	N	49387756	508268	130057053	71790	5:55
MH0044	N	44865220	395222	115528200	266726	5:07
MH0045	N	49439210	461465	119620847	155303	5:03
MH0046	N	35802102	378975	94974924	101419	4:25
MH0047	N	26934980	215030	57895950	137473	4:28
MH0048	N	26978336	311909	65644555	84104	3:44
MH0049	N	30656592	198018	59476773	138240	3:56
MH0050	N	47794456	513211	125199765	209364	5:28
MH0051	N	25971564	313411	71104852	35831	4:06
MH0052	N	28577486	357433	81152613	34241	3:59
MH0053	N	43286304	391940	107008498	65849	5:15
MH0054	N	42267078	417833	118830901	69352	5:10
MH0055	N	47116588	456959	122201575	113972	5:26
MH0056	N	49304102	535912	109807210	56331	5:49
MH0057	N	43053324	270584	79354046	140930	4:27
MH0058	N	49833480	530008	134848584	130408	7:01
MH0059	N	41036844	591956	140331726	137703	5:53
MH0060	N	41114502	451169	105335228	78398	5:08
MH0061	N	44586960	245046	86382079	123577	4:28
MH0062	N	37106156	421940	106860875	123112	4:36
MH0063	N	45964540	401907	100119143	138218	5:01
MH0064	N	44736812	412964	98946675	83252	4:47
MH0065	N	44585626	539382	122865285	172548	6:36
MH0066	N	36117084	429794	102410038	44728	4:33
MH0067	N	44485830	301281	105721471	139383	4:31
MH0068	N	43871052	317502	95659301	120767	4:28
MH0069	N	69732814	526256	139988599	170690	6:08
MH0070	N	47929526	529726	135784415	268207	6:38
MH0071	N	46249674	428683	110732392	110890	5:02
MH0072	N	49297218	356475	96050816	95609	5:01
MH0073	N	47239818	355164	104212364	118406	4:49
MH0074	N	46032014	338221	104564599	153749	4:48
MH0075	N	47065984	488754	122144561	160277	5:24
MH0076	N	30657846	406742	96077349	68346	4:34
MH0077	N	49671284	531236	133134913	153497	7:02
MH0078	N	26055172	146778	38007790	74837	3:29
MH0079	N	27422848	302463	73619051	174281	4:25
MH0080	N	46597396	510697	132839614	151294	5:29
MH0081	N	47852459	509017	133067703	250772	5:59
MH0082	N	48353598	525957	134749581	205145	6:12
MH0083	N	47905438	471034	130094125	85667	6:06

MH0084	N	53570582	222216	57689929	103684	4:47
MH0085	N	49436638	354772	103674500	122024	4:48
MH0086	N	55436742	615206	163381790	85993	5:54
O2.UC-11	Y	38513652	244442	62847655	174510	5:32
O2.UC-12	Y	36913096	332777	81539283	104474	4:17
O2.UC-13	Y	43623490	371729	104525967	59043	4:48
O2.UC-14	Y	26944888	184961	44707281	76040	3:40
O2.UC-16	Y	42270010	188686	46599989	46107	4:42
O2.UC-17	Y	40306326	203286	50510566	106832	5:16
O2.UC-18	Y	41915008	267554	81548198	126060	4:23
O2.UC-19	Y	38460916	434435	108615044	92883	4:48
O2.UC-1	Y	42575162	361648	87515158	67491	4:27
O2.UC-20	Y	38378976	384972	99727759	86605	4:25
O2.UC-21	Y	34840550	250920	59451062	55343	4:32
O2.UC-22	Y	44248020	473338	117430127	86092	4:53
O2.UC-23	Y	38656482	310943	77040510	76378	4:35
O2.UC-24	Y	41673838	369340	88532980	71510	4:55
O2.UC-4	Y	43272944	337639	93193172	84016	4:24
V1.CD-11	N	51391696	284504	99344197	102833	4:57
V1.CD-12	Y	40610694	186790	71180559	99861	5:10
V1.CD-13	N	42562886	478445	119472769	102396	5:16
V1.CD-14	N	44025748	547055	130629184	81599	5:38
V1.CD-15	Y	40652058	187201	69088686	97686	4:36
V1.CD-1	Y	47138630	203634	67698189	119498	4:28
V1.CD-2	N	46725664	210277	54510396	72527	4:08
V1.CD-3	N	45368268	333224	84945860	179325	4:43
V1.CD-4	N	42941570	440556	116509969	79647	5:09
V1.CD-6	Y	42599132	341147	88487599	88171	4:32
V1.CD-8	N	49914030	551474	132401995	72859	6:46
V1.CD-9	N	47142346	380253	111101210	91191	4:50
V1.UC-10	Y	42187572	549763	130377362	136740	5:48
V1.UC-13	Y	38944124	375970	108376694	84398	4:26
V1.UC-14	Y	49645840	408344	116886829	123686	5:13
V1.UC-15	Y	46711104	418000	114122667	151163	5:06
V1.UC-17	Y	35523980	358390	88025111	29089	4:15
V1.UC-18	N	48385658	369172	106999653	116163	5:10
V1.UC-19	N	49238118	473194	128496132	330864	5:34
V1.UC-21	Y	46362496	382460	105530619	128907	4:58
V1.UC-6	N	43160944	620540	149233782	126763	6:20
V1.UC-7	N	36944164	253405	67524998	109311	5:08
V1.UC-8	N	46693474	546670	145054113	108830	5:46
V1.UC-9	N	46910726	511461	121407469	172249	5:34

References

[1] Qin, J., Li, R., Raes, J., Arumugam, M., Burgdorf, K.S. *et al.* A human gut microbial gene catalogue established by metagenomic sequencing. *Nature* **464**, 59–65 (2010).

Supplementary Table 4: List of genomes used for coloring de Bruijn graphs. There are 1492 finished bacterial genomes and 1744 draft bacterial genomes listed. For draft genomes, only the accession of the first sequence is listed.

Name	Accession
Homo sapiens	version GRCh37.p5
Acaryochloris marina MBIC11017	NC_009925.1; NC_009926.1; NC_009927.1; NC_009928.1; NC_009929.1; NC_009930.1; NC_009931.1; NC_009932.1; NC_009933.1; NC_009934.1
Acetobacter pasteurianus IFO 3283 01	NC_013209.1; NC_013210.1; NC_013211.1; NC_013212.1; NC_013213.1; NC_013214.1; NC_013215.1
Acetohalobium arabaticum DSM 5501	NC_014378.1
Acholeplasma laidlawii PG 8A	NC_010163.1
Achromobacter xylosoxidans A8	NC_014640.1; NC_014641.1; NC_014642.1
Acidaminococcus fermentans DSM 20731	NC_013740.1
Acidaminococcus intestini RyC MR95	NC_016077.1
Acidianus hospitalis W1	NC_015518.1
Acidilobus saccharovorans 345 15	NC_014374.1
Acidimicrobium ferrooxidans DSM 10331	NC_013124.1
Acidiphilium cryptum JF 5	NC_009467.1; NC_009468.1; NC_009469.1; NC_009470.1; NC_009471.1; NC_009472.1; NC_009473.1; NC_009474.1; NC_009484.1
Acidiphilium multivorum AIU301	NC_015178.1; NC_015179.1; NC_015180.1; NC_015181.1; NC_015182.1; NC_015186.1; NC_015187.1; NC_015188.1; NC_015189.1
Acidithiobacillus caldus SM 1	NC_015850.1; NC_015851.1; NC_015852.1; NC_015853.1; NC_015854.1
Acidithiobacillus ferrivorans SS3	NC_015942.1
Acidithiobacillus ferrooxidans ATCC 23270	NC_011761.1
Acidithiobacillus ferrooxidans ATCC 53993	NC_011206.1
Acidobacterium capsulatum ATCC 51196	NC_012483.1
Acidobacterium MP5ACTX9	NC_015057.1; NC_015058.1; NC_015059.1; NC_015060.1; NC_015064.1; NC_015065.1
Acidothermus cellulolyticus 11B	NC_008578.1
Acidovorax avenae ATCC 19860	NC_015138.1
Acidovorax citrulli AAC00 1	NC_008752.1
Acidovorax ebreus TPSY	NC_011992.1
Acidovorax JS42	NC_008765.1; NC_008766.1; NC_008782.1
Aciduliprofundum boonei T469	NC_013926.1
Acinetobacter ADP1	NC_005966.1
Acinetobacter baumannii AB0057	NC_011585.1; NC_011586.1
Acinetobacter baumannii AB307 0294	NC_011595.1
Acinetobacter baumannii ACICU	NC_010605.1; NC_010606.1; NC_010611.1
Acinetobacter baumannii ATCC 17978	NC_009083.1; NC_009084.1; NC_009085.1
Acinetobacter baumannii AYE	NC_010401.1; NC_010402.1; NC_010403.1; NC_010404.1; NC_010410.1
Acinetobacter baumannii SDF	NC_010395.1; NC_010396.1; NC_010398.1; NC_010400.1
Acinetobacter DR1	NC_014259.1
Actinobacillus pleuropneumoniae serovar 3 JL03	NC_010278.1
Actinobacillus pleuropneumoniae serovar 5b L20	NC_009053.1
Actinobacillus pleuropneumoniae serovar 7 AP76	NC_010939.1; NC_010940.1; NC_010941.1; NC_010942.1
Actinobacillus succinogenes 130Z	NC_009655.1
Actinosynnema mirum DSM 43827	NC_013093.1
Aerococcus urinae ACS 120 V Col10a	NC_015278.1
Aeromonas hydrophila ATCC 7966	NC_008570.1
Aeromonas salmonicida A449	NC_004923.1; NC_004924.1; NC_004925.1; NC_009348.1; NC_009349.1; NC_009350.1
Aeromonas veronii B565	NC_015424.1
Aeropyrum pernix K1	NC_000854.2
Aggregatibacter actinomycetemcomitans D11S 1	NC_013416.1; NC_013438.1; NC_013597.1; NC_014629.1
Aggregatibacter aphrophilus NJ8700	NC_012913.1
Agrobacterium H13 3	NC_015183.1; NC_015184.1; NC_015508.1
Agrobacterium radiobacter K84	NC_011983.1; NC_011985.1; NC_011987.1; NC_011990.1; NC_011994.1
Agrobacterium tumefaciens C58	NC_003062.2; NC_003063.2; NC_003064.2; NC_003065.3
Agrobacterium vitis S4	NC_011981.1; NC_011982.1; NC_011984.1; NC_011986.1; NC_011988.1; NC_011989.1; NC_011991.1

Akkermansia muciniphila ATCC BAA 835	NC_010655.1
Alcanivorax borkumensis SK2	NC_008260.1
Alicycliphilus denitrificans BC	NC_014908.1; NC_014910.1; NC_014911.1
Alicycliphilus denitrificans K601	NC_015422.1; NC_015423.1
Alicyclobacillus acidocaldarius DSM 446	NC_013205.1; NC_013206.1; NC_013207.1; NC_013208.1
Aliivibrio salmonicida LFI1238	NC_011311.1; NC_011312.1; NC_011313.1; NC_011314.1; NC_011315.1; NC_011316.1
Alkalilimnicola ehrlichii MLHE 1	NC_008340.1
Alkaliphilus metalliredigens QYMF	NC_009633.1
Alkaliphilus oremlandii OhILAs	NC_009922.1
Allochromatium vinosum DSM 180	NC_013851.1; NC_013852.1; NC_013862.1
Alteromonas macleodii Deep ecotype	NC_011138.2
Alteromonas SN2	NC_015554.1
Aminobacterium colombiense DSM 12261	NC_014011.1
Ammonifex degensii KC4	NC_013385.1; NC_013386.1
Amycolatopsis mediterranei U32	NC_014318.1
Amycolicicoccus subflavus DQS3 9A1	NC_015560.1; NC_015561.1; NC_015564.1
Anabaena variabilis ATCC 29413	NC_007410.1; NC_007411.1; NC_007412.1; NC_007413.1; NC_014000.1
Anaerococcus prevotii DSM 20548	NC_013164.1; NC_013171.1
Anaerolinea thermophila UNI 1	NC_014960.1
Anaeromyxobacter dehalogenans 2CP 1	NC_011891.1
Anaeromyxobacter dehalogenans 2CP C	NC_007760.1
Anaeromyxobacter Fw109 5	NC_009675.1
Anaeromyxobacter K	NC_011145.1
Anaplasma centrale Israel	NC_013532.1
Anaplasma marginale Florida	NC_012026.1
Anaplasma marginale Maries	NC_004842.2
Anaplasma phagocytophilum HZ	NC_007797.1
Anoxybacillus flavithermus WK1	NC_011567.1
Aquifex aeolicus VF5	NC_000918.1; NC_001880.1
Arcanobacterium haemolyticum DSM 20595	NC_014218.1
Archaeoglobus fulgidus DSM 4304	NC_000917.1
Archaeoglobus profundus DSM 5631	NC_013741.1; NC_013742.1
Archaeoglobus veneficus SNP6	NC_015320.1
Arcobacter butzleri RM4018	NC_009850.1
Arcobacter nitrofigilis DSM 7299	NC_014166.1
Aromatoleum aromaticum EbN1	NC_006513.1; NC_006823.1; NC_006824.1
Arthrobacter arilaitensis Re117	NC_014548.1; NC_014549.1; NC_014550.1
Arthrobacter aurescens TC1	NC_008711.1; NC_008712.1; NC_008713.1
Arthrobacter chlorophenolicus A6	NC_011879.1; NC_011881.1; NC_011886.1
Arthrobacter FB24	NC_008537.1; NC_008538.1; NC_008539.1; NC_008541.1
Arthrobacter phenanthrenivorans Sphe3	NC_015145.1; NC_015146.1; NC_015147.1
Aster yellows witches broom phytoplasma AYWB	NC_007716.1; NC_007717.1; NC_007718.1; NC_007719.1; NC_007720.1
Asticcacaulis excentricus CB 48	NC_014816.1; NC_014817.1; NC_014818.1; NC_014819.1
Atopobium parvulum DSM 20469	NC_013203.1
Azoarcus BH72	NC_008702.1
Azorhizobium caulinodans ORS 571	NC_009937.1
Azospirillum B510	NC_013854.1; NC_013855.1; NC_013856.1; NC_013857.1; NC_013858.1; NC_013859.1; NC_013860.1
Azotobacter vinelandii DJ	NC_012560.1
Bacillus amyloliquefaciens DSM 7	NC_014551.1
Bacillus amyloliquefaciens FZB42	NC_009725.1
Bacillus anthracis A0248	NC_012655.1; NC_012656.1; NC_012659.1
Bacillus anthracis Ames Ancestor	NC_007322.2; NC_007323.3; NC_007530.2
Bacillus anthracis Ames	NC_003997.3
Bacillus anthracis CDC 684	NC_012577.1; NC_012579.1; NC_012581.1
Bacillus anthracis Sterne	NC_005945.1
Bacillus atrophaeus 1942	NC_014639.1
Bacillus cellulosilyticus DSM 2522	NC_014829.1

Bacillus cereus 03BB102	NC_012472.1; NC_012473.1
Bacillus cereus AH187	NC_011654.1; NC_011655.1; NC_011656.1; NC_011657.1; NC_011658.1
Bacillus cereus AH820	NC_011771.1; NC_011773.1; NC_011776.1; NC_011777.1
Bacillus cereus ATCC 10987	NC_003909.8; NC_005707.1
Bacillus cereus ATCC 14579	NC_004721.2; NC_004722.1
Bacillus cereus B4264	NC_011725.1
Bacillus cereus biovar anthracis CI	NC_014331.1; NC_014332.1; NC_014333.1; NC_014335.1
Bacillus cereus E33L	NC_006274.1; NC_007103.1; NC_007104.1; NC_007105.1; NC_007106.1; NC_007107.1
Bacillus cereus G9842	NC_011772.1; NC_011774.1; NC_011775.1
Bacillus cereus Q1	NC_011969.1; NC_011971.1; NC_011973.1
Bacillus clausii KSM K16	NC_006582.1
Bacillus coagulans 2 6	NC_015634.1
Bacillus coagulans 36D1	NC_016023.1
Bacillus cytotoxicus NVH 391 98	NC_009673.1; NC_009674.1
Bacillus halodurans C 125	NC_002570.2
Bacillus licheniformis ATCC 14580	NC_006270.3
Bacillus licheniformis ATCC 14580	NC_006322.1
Bacillus megaterium DSM319	NC_014103.1
Bacillus megaterium QM B1551	NC_004604.2; NC_010008.2; NC_010009.2; NC_010010.2;
NC_014019.1; NC_014023.1; NC_014025.1; NC_014031.1	
Bacillus pseudofirmus OF4	NC_013791.2; NC_013792.1; NC_013793.1
Bacillus pumilus SAFR 032	NC_009848.1
Bacillus selenitireducens MLS10	NC_014219.1
Bacillus subtilis 168	NC_000964.3
Bacillus subtilis BSn5	NC_014976.1
Bacillus subtilis spizizenii TU B 10	NC_016047.1
Bacillus subtilis spizizenii W23	NC_014479.1
Bacillus thuringiensis Al Hakam	NC_008598.1; NC_008600.1
Bacillus thuringiensis BMB171	NC_014171.1; NC_014172.1
Bacillus thuringiensis serovar konkukian 97 27	NC_005957.1; NC_006578.1
Bacillus tusciae DSM 2912	NC_014098.1
Bacillus weihenstephanensis KBAB4	NC_010180.1; NC_010181.1; NC_010182.1; NC_010183.1; NC_010184.1
Bacteroides fragilis NCTC 9343	NC_003228.3; NC_006873.1
Bacteroides fragilis YCH46	NC_006297.1; NC_006347.1
Bacteroides helcogenes P 36 108	NC_014933.1
Bacteroides salanitronis DSM 18170	NC_015164.1; NC_015165.1; NC_015166.1; NC_015168.1
Bacteroides thetaiotaomicron VPI 5482	NC_004663.1; NC_004703.1
Bacteroides vulgatus ATCC 8482	NC_009614.1
Bartonella bacilliformis KC583	NC_008783.1
Bartonella clarridgeiae 73	NC_014932.1
Bartonella grahamii as4aup	NC_012846.1; NC_012847.1
Bartonella henselae Houston 1	NC_005956.1
Bartonella quintana Toulouse	NC_005955.1
Bartonella tribocorum CIP 105476	NC_010160.1; NC_010161.1
Baumannia cicadellinicola Hc Homalodisca coagulata	NC_007984.1
Bdellovibrio bacteriovorus HD100	NC_005363.1
Beijerinckia indica ATCC 9039	NC_010578.1; NC_010580.1; NC_010581.1
Beutenbergia cavernae DSM 12333	NC_012669.1
Bifidobacterium adolescentis ATCC 15703	NC_008618.1
Bifidobacterium animalis lactis AD011	NC_011835.1
Bifidobacterium animalis lactis Bl 04	NC_012814.1
Bifidobacterium animalis lactis DSM 10140	NC_012815.1
Bifidobacterium bifidum PRL2010	NC_014638.1
Bifidobacterium bifidum S17	NC_014616.1
Bifidobacterium dentium Bd1	NC_013714.1
Bifidobacterium longum BBMN68	NC_014656.1
Bifidobacterium longum DJO10A	NC_004252.1; NC_004253.1; NC_010816.1

Bifidobacterium longum infantis 157F	NC_015052.1; NC_015053.1; NC_015066.1
Bifidobacterium longum infantis ATCC 15697	NC_011593.1
Bifidobacterium longum JCM 1217	NC_015067.1
Bifidobacterium longum JDM301	NC_014169.1
Bifidobacterium longum NCC2705	NC_004307.2; NC_004943.1
Blattabacterium Blattella germanica Bge	NC_013454.1; NC_015679.1
Blattabacterium Mastotermes darwiniensis MADAR	NC_016146.1; NC_016150.1
Blattabacterium Periplaneta americana BPLAN	NC_013418.2; NC_013419.1
Bordetella avium 197N	NC_010645.1
Bordetella bronchiseptica RB50	NC_002927.3
Bordetella parapertussis 12822	NC_002928.3
Bordetella pertussis Tohama I	NC_002929.2
Bordetella petrii DSM 12804	NC_010170.1
Borrelia afzelii PKo	NC_008273.1; NC_008274.1; NC_008277.1; NC_008564.1; NC_008565.1; NC_008566.1;
	NC_008567.1; NC_008568.1; NC_008569.1; NT_167350.1; NT_167351.1; NT_167352.1
Borrelia bissettii DN127	NC_015903.1; NC_015904.1; NC_015905.1; NC_015906.1; NC_015907.1;
	NC_015908.1; NC_015909.1; NC_015910.1; NC_015911.1; NC_015915.1; NC_015916.1; NC_015917.1;
	NC_015918.1; NC_015919.1; NC_015920.1; NC_015921.1; NC_015922.1
Borrelia burgdorferi B31	NC_000948.1; NC_000949.1; NC_000950.1; NC_000951.1; NC_000952.1;
	NC_000953.1; NC_000954.1; NC_000955.1; NC_000956.1;
	NC_000957.1; NC_001318.1; NC_001849.1; NC_001850.1;
	NC_001851.1; NC_001852.1; NC_001853.1; NC_001854.1;
NC_001855.1; NC_001856.1; NC_001857.1; NC_001903.1; NC_001904.1	
Borrelia burgdorferi ZS7	NC_011720.1; NC_011722.1; NC_011724.1; NC_011728.1; NC_011731.1; NC_011735.1;
	NC_011736.1; NC_011778.1; NC_011779.1; NC_011780.1; NC_011781.1;
	NC_011782.1; NC_011783.1; NC_011784.1; NC_011785.1
Borrelia duttonii Ly	NC_011224.1; NC_011226.1; NC_011229.1; NC_011245.1; NC_011247.1; NC_011248.1;
	NC_011249.1; NC_011250.1; NC_011251.1; NC_011254.1; NC_011256.1; NC_011257.1;
NC_011259.1; NC_011261.1; NC_011262.1; NC_011264.1; NC_011265.1	
Borrelia garinii PBi	NC_006128.1; NC_006129.1; NC_006156.1
Borrelia hermsii DAH	NC_010673.1
Borrelia recurrentis A1	NC_011244.1; NC_011246.1; NC_011252.1; NC_011253.1;
NC_011255.1; NC_011258.1; NC_011260.1; NC_011263.1	
Borrelia turicatae 91E135	NC_008710.1
Brachybacterium faecium DSM 4810	NC_013172.1
Brachyspira hyodysenteriae WA1	NC_012225.1; NC_012226.1
Brachyspira murdochii DSM 12563	NC_014150.1
Brachyspira pilosicoli 95 1000	NC_014330.1
Bradyrhizobium BTAi1	NC_009475.1; NC_009485.1
Bradyrhizobium japonicum USDA 110	NC_004463.1
Bradyrhizobium ORS 278	NC_009445.1
Brevibacillus brevis NBRC 100599	NC_012491.1
Brevundimonas subvibrioides ATCC 15264	NC_014375.1
Brucella abortus bv 1 9 941	NC_006932.1; NC_006933.1
Brucella abortus S19	NC_010740.1; NC_010742.1
Brucella canis ATCC 23365	NC_010103.1; NC_010104.1
Brucella melitensis ATCC 23457	NC_012441.1; NC_012442.1
Brucella melitensis biovar Abortus 2308	NC_007618.1; NC_007624.1
Brucella melitensis bv 1 16M	NC_003317.1; NC_003318.1
Brucella microti CCM 4915	NC_013118.1; NC_013119.1
Brucella ovis ATCC 25840	NC_009504.1; NC_009505.1
Brucella pinnipedialis B2 94	NC_015857.1; NC_015858.1
Brucella suis 1330	NC_004310.3; NC_004311.2
Brucella suis ATCC 23445	NC_010167.1; NC_010169.1
Buchnera aphidicola 5A Acyrthosiphon pisum	NC_011833.1
Buchnera aphidicola APS Acyrthosiphon pisum	NC_002252.1; NC_002253.1; NC_002528.1
Buchnera aphidicola Bp Baizongia pistaciae	NC_004545.1; NC_004555.1

Buchnera aphidicola Cc Cinara cedri	NC_008513.1; NC_011878.1
Buchnera aphidicola Cinara tujafilina	NC_015662.1
Buchnera aphidicola Sg Schizaphis graminum	NC_004061.1
Buchnera aphidicola Tuc7 Acyrthosiphon pisum	NC_011834.1
Burkholderia 383	NC_007509.1; NC_007510.1; NC_007511.1
Burkholderia ambifaria AMMD	NC_008385.1; NC_008390.1; NC_008391.1; NC_008392.1
Burkholderia ambifaria MC40 6	NC_010551.1; NC_010552.1; NC_010553.1; NC_010557.1
Burkholderia CCGE1001	NC_015136.1; NC_015137.1
Burkholderia CCGE1002	NC_014117.1; NC_014118.1; NC_014119.1; NC_014120.1
Burkholderia CCGE1003	NC_014539.1; NC_014540.1
Burkholderia cenocepacia AU 1054	NC_008060.1; NC_008061.1; NC_008062.1
Burkholderia cenocepacia HI2424	NC_008542.1; NC_008543.1; NC_008544.1; NC_008545.1
Burkholderia cenocepacia J2315	NC_011000.1; NC_011001.1; NC_011002.1; NC_011003.1
Burkholderia cenocepacia MC0 3	NC_010508.1; NC_010512.1; NC_010515.1
Burkholderia gladioli BSR3	NC_015376.1; NC_015377.1; NC_015378.1; NC_015381.1; NC_015382.1; NC_015383.1
Burkholderia glumae BGR1	NC_012718.1; NC_012720.2; NC_012721.2; NC_012723.1; NC_012724.2; NC_012725.2
Burkholderia JV3	NC_015947.1
Burkholderia mallei ATCC 23344	NC_006348.1; NC_006349.2
Burkholderia mallei NCTC 10229	NC_008835.1; NC_008836.1
Burkholderia mallei NCTC 10247	NC_009079.1; NC_009080.1
Burkholderia mallei SAVP1	NC_008784.1; NC_008785.1
Burkholderia multivorans ATCC 17616	NC_010070.1; NC_010084.1; NC_010086.1; NC_010087.1
Burkholderia multivorans ATCC 17616	NC_010801.1; NC_010802.1; NC_010804.1; NC_010805.1
Burkholderia phymatum STM815	NC_010622.1; NC_010623.1; NC_010625.1; NC_010627.1
Burkholderia phytofirmans PsJN	NC_010676.1; NC_010679.1; NC_010681.1
Burkholderia pseudomallei 1106a	NC_009076.1; NC_009078.1
Burkholderia pseudomallei 1710b	NC_007434.1; NC_007435.1
Burkholderia pseudomallei 668	NC_009074.1; NC_009075.1
Burkholderia pseudomallei K96243	NC_006350.1; NC_006351.1
Burkholderia pseudomallei MSHR346	NC_012695.1
Burkholderia rhizoxinica HKI 454	NC_014718.1; NC_014722.1; NC_014723.1
Burkholderia thailandensis E264	NC_007650.1; NC_007651.1
Burkholderia vietnamiensis G4	NC_009226.1; NC_009227.1; NC_009228.1; NC_009229.1;
NC_009230.1; NC_009254.1; NC_009255.1; NC_009256.1	
Burkholderia xenovorans LB400	NC_007951.1; NC_007952.1; NC_007953.1
Butyrivibrio proteoclasticus B316	NC_014387.1; NC_014388.1; NC_014389.1; NC_014390.1
Caldicellulosiruptor bescii DSM 6725	NC_012034.1; NC_012036.1; NC_012037.1
Caldicellulosiruptor hydrothermalis 108	NC_014652.1
Caldicellulosiruptor kristjanssonii 177R1B	NC_014719.1; NC_014721.1
Caldicellulosiruptor kronotskyensis 2002	NC_014720.1
Caldicellulosiruptor lactoaceticus 6A	NC_015949.1
Caldicellulosiruptor obsidiansis OB47	NC_014392.1
Caldicellulosiruptor owensensis OL	NC_014657.1
Caldicellulosiruptor saccharolyticus DSM 8903	NC_009437.1
Calditerrivibrio nitroreducens DSM 19672	NC_014749.1; NC_014758.1
Caldivirga maquilingensis IC 167	NC_009954.1
Campylobacter concisus 13826	NC_009795.1; NC_009796.1; NC_009802.1
Campylobacter curvus 525 92	NC_009715.1
Campylobacter fetus 82 40	NC_008599.1
Campylobacter hominis ATCC BAA 381	NC_009713.1; NC_009714.1
Campylobacter jejuni 81116	NC_009839.1
Campylobacter jejuni 81 176	NC_008770.1; NC_008787.1; NC_008790.1
Campylobacter jejuni doylei 269 97	NC_009707.1
Campylobacter jejuni ICDCCJ07001	NC_014801.1; NC_014802.1
Campylobacter jejuni NCTC 11168	NC_002163.1
Campylobacter jejuni RM1221	NC_003912.7
Campylobacter lari RM2100	NC_012039.1; NC_012040.1

Candidatus Accumulibacter phosphatis clade IIA UW 1	NC_013190.1; NC_013191.1; NC_013193.1; NC_013194.1
Candidatus Amoebophilus asiaticus 5a2	NC_010830.1
Candidatus Arthromitus SFB mouse Japan	NC_015913.1
Candidatus Arthromitus SFB rat Yit	NC_016012.1
Candidatus Azobacteroides pseudotrichonymphae genomovar CFP2	NC_011561.1; NC_011562.1; NC_011563.1; NC_011564.1; NC_011565.1
Candidatus Blochmannia floridanus	NC_005061.1
Candidatus Blochmannia pennsylvanicus BPEN	NC_007292.1
Candidatus Blochmannia vafer BVAF	NC_014909.1
Candidatus Carsonella ruddii	NC_008512.1
Candidatus Chloracidobacterium thermophilum B	NC_016024.1; NC_016025.1
Candidatus Cloacamonas acidaminovorans	NS_000195.1
Candidatus Desulforudis audaxviator MP104C	NC_010424.1
Candidatus Hamiltonella defensa 5AT Acyrthosiphon pisum	NC_012751.1; NC_012752.1
Candidatus Hodgkinia cicadicola Dsem	NC_012960.1
Candidatus Korarchaeum cryptofilum OPF8	NC_010482.1
Candidatus Koribacter versatilis Ellin345	NC_008009.1
Candidatus Liberibacter asiaticus psy62	NC_012985.3
Candidatus Liberibacter solanacearum CLso ZC1	NC_014774.1
Candidatus Midichloria mitochondrii IricVA	NC_015722.1
Candidatus Moranella endobia PCIT	NC_015735.1
Candidatus Nitrospira defluvii	NC_014355.1
Candidatus Pelagibacter IMCC9063	NC_015380.1
Candidatus Pelagibacter ubique HTCC1062	NC_007205.1
Candidatus Phytoplasma australiense	NC_010544.1
Candidatus Phytoplasma mali	NC_011047.1
Candidatus Protochlamydia amoebophila UWE25	NC_005861.1
Candidatus Puniceispirillum marinum IMCC1322	NC_014010.1
Candidatus Riesia pediculicola USDA	NC_013962.1; NC_014109.1
Candidatus Ruthia magnifica Cm Calyptogena magnifica	NC_008610.1
Candidatus Solibacter usitatus Ellin6076	NC_008536.1
Candidatus Sulcia muelleri CARI	NC_014499.1
Candidatus Sulcia muelleri DMIN	NC_014004.1
Candidatus Sulcia muelleri GWSS	NC_010118.1
Candidatus Sulcia muelleri SMDSEM	NC_013123.1
Candidatus Tremblaya princeps PCIT	NC_015736.1
Candidatus Vesicomyosocius okutanii HA	NC_009465.1
Candidatus Zinderia insecticola CARI	NC_014497.1
Capnocytophaga canimorsus Cc5	NC_015846.1
Capnocytophaga ochracea DSM 7271	NC_013162.1
Carboxydothermus hydrogenoformans Z 2901	NC_007503.1
Carnobacterium 17 4	NC_015390.1; NC_015391.1
Catenulispora acidiphila DSM 44928	NC_013131.1
Caulobacter crescentus CB15	NC_002696.2
Caulobacter crescentus NA1000	NC_011916.1
Caulobacter K31	NC_010333.1; NC_010335.1; NC_010338.1
Caulobacter segnis ATCC 21756	NC_014100.1
Cellulomonas fimi ATCC 484	NC_015514.1
Cellulomonas flavigena DSM 20109	NC_014151.1
Cellulophaga algicola DSM 14237	NC_014934.1
Cellulophaga lytica DSM 7489	NC_015167.1
Cellvibrio gilvus ATCC 13127	NC_015671.1
Cellvibrio japonicus Ueda107	NC_010995.1
Cenarchaeum symbiosum A	NC_014820.1
Chelativorans BNC1	NC_008242.1; NC_008243.1; NC_008244.1; NC_008254.1
Chitinophaga pinensis DSM 2588	NC_013132.1
Chlamydia muridarum Nigg	NC_002182.1; NC_002620.2
Chlamydia trachomatis 434 Bu	NC_010287.1

Chlamydia trachomatis A HAR 13	NC_007429.1; NC_007430.1
Chlamydia trachomatis B Jali20 OT	NC_012686.1
Chlamydia trachomatis B TZ1A828 OT	NC_012687.1
Chlamydia trachomatis D UW 3 CX	NC_000117.1
Chlamydia trachomatis L2b UCH 1 proctitis	NC_010280.2
Chlamydia trachomatis L2c	NC_015744.1
Chlamydophila abortus S26 3	NC_004552.2
Chlamydophila caviae GPIC	NC_003361.3; NC_004720.1
Chlamydophila felis Fe C 56	NC_007899.1; NC_007900.1
Chlamydophila pecorum E58	NC_015408.1
Chlamydophila pneumoniae AR39	NC_002179.2
Chlamydophila pneumoniae CWL029	NC_000922.1
Chlamydophila pneumoniae J138	NC_002491.1
Chlamydophila pneumoniae TW 183	NC_005043.1
Chlamydophila psittaci 6BC	NC_015217.1; NC_015470.1
Chlorobaculum parvum NCIB 8327	NC_011027.1
Chlorobium chlorochromatii CaD3	NC_007514.1
Chlorobium limicola DSM 245	NC_010803.1
Chlorobium luteolum DSM 273	NC_007512.1
Chlorobium phaeobacteroides BS1	NC_010831.1
Chlorobium phaeobacteroides DSM 266	NC_008639.1
Chlorobium phaeovibrioides DSM 265	NC_009337.1
Chlorobium tepidum TLS	NC_002932.3
Chloroflexus aggregans DSM 9485	NC_011831.1
Chloroflexus aurantiacus J 10 fl	NC_010175.1
Chloroflexus Y 400 fl	NC_012032.1
Chloroherpeton thalassium ATCC 35110	NC_011026.1
Chromobacterium violaceum ATCC 12472	NC_005085.1
Chromohalobacter salexigens DSM 3043	NC_007963.1
Citrobacter koseri ATCC BAA 895	NC_009792.1; NC_009793.1; NC_009794.1
Citrobacter rodentium ICC168	NC_013716.1; NC_013717.1; NC_013718.1; NC_013719.1
Clavibacter michiganensis NCPPB 382	NC_009478.1; NC_009479.1; NC_009480.1
Clavibacter michiganensis sepedonicus	NC_010399.1; NC_010407.1; NC_010408.1
Clostridiales genomosp BVAB3 UPII9 5	NC_013895.2
Clostridium acetobutylicum ATCC 824	NC_001988.2; NC_003030.1
Clostridium acetobutylicum DSM 1731	NC_015686.1; NC_015687.1; NC_015688.1
Clostridium beijerinckii NCIMB 8052	NC_009617.1
Clostridium botulinum A2 Kyoto	NC_012563.1
Clostridium botulinum A3 Loch Maree	NC_010418.1; NC_010520.1
Clostridium botulinum A ATCC 19397	NC_009697.1
Clostridium botulinum A ATCC 3502	NC_009495.1; NC_009496.1
Clostridium botulinum A Hall	NC_009698.1
Clostridium botulinum B1 Okra	NC_010379.1; NC_010516.1
Clostridium botulinum Ba4 657	NC_012654.1; NC_012657.1; NC_012658.1
Clostridium botulinum B Eklund 17B	NC_010674.1; NC_010680.1
Clostridium botulinum BKT015925	NC_015417.1; NC_015418.1; NC_015419.1; NC_015425.1; NC_015426.1; NC_015427.1
Clostridium botulinum E3 Alaska E43	NC_010723.1
Clostridium botulinum F Langeland	NC_009699.1; NC_009700.1
Clostridium cellulolyticum H10	NC_011898.1
Clostridium cellulovorans 743B	NC_014393.1
Clostridium difficile 630	NC_008226.1; NC_009089.1
Clostridium difficile CD196	NC_013315.1
Clostridium difficile R20291	NC_013316.1
Clostridium kluyveri DSM 555	NC_009466.1; NC_009706.1
Clostridium kluyveri NBRC 12016	NC_011836.1; NC_011837.1
Clostridium lentocellum DSM 5427	NC_015275.1
Clostridium ljungdahlii DSM 13528	NC_014328.1

Clostridium novyi NT	NC_008593.1
Clostridium perfringens 13	NC_003042.1; NC_003366.1
Clostridium perfringens ATCC 13124	NC_008261.1
Clostridium perfringens SM101	NC_008262.1; NC_008263.1; NC_008264.1; NC_008265.1
Clostridium phytofermentans ISDg	NC_010001.1
Clostridium saccharolyticum WM1	NC_014376.1
Clostridium sticklandii DSM 519	NC_014614.1
Clostridium SY8519	NC_015737.1
Clostridium tetani E88	NC_004557.1; NC_004565.1
Clostridium thermocellum ATCC 27405	NC_009012.1
Collimonas fungivorans Ter331	NC_015856.1
Colwellia psychrerythraea 34H	NC_003910.7
Comamonas testosteroni CNB 2	NC_010935.1; NC_013446.1
Conexibacter woesei DSM 14684	NC_013739.1
Coprothermobacter proteolyticus DSM 5265	NC_011295.1
Coraliomargarita akajimensis DSM 45221	NC_014008.1
Coriobacterium glomerans PW2	NC_015389.1
Corynebacterium aurimucosum ATCC 700975	NC_010813.1; NC_012590.1
Corynebacterium diphtheriae NCTC 13129	NC_002935.2
Corynebacterium efficiens YS 314	NC_004319.1; NC_004320.1; NC_004369.1
Corynebacterium glutamicum ATCC 13032	NC_003450.3
Corynebacterium glutamicum ATCC 13032	NC_006958.1
Corynebacterium glutamicum R	NC_009342.1; NC_009343.1
Corynebacterium jeikeium K411	NC_003080.1; NC_007164.1
Corynebacterium kroppenstedtii DSM 44385	NC_012704.1
Corynebacterium pseudotuberculosis FRC41	NC_014329.1
Corynebacterium resistens DSM 45100	NC_015673.1
Corynebacterium ulcerans BR AD22	NC_015683.1
Corynebacterium urealyticum DSM 7109	NC_010545.1
Corynebacterium variabile DSM 44702	NC_015859.1
Coxiella burnetii CbuG Q212	NC_011527.1
Coxiella burnetii CbuK Q154	NC_011526.1; NC_011528.1
Coxiella burnetii Dugway 5J108 111	NC_009726.1; NC_009727.1
Coxiella burnetii RSA 331	NC_010115.1; NC_010117.1
Coxiella burnetii RSA 493	NC_002971.3; NC_004704.1
Croceibacter atlanticus HTCC2559	NC_014230.1
Cronobacter sakazakii ATCC BAA 894	NC_009778.1; NC_009779.1; NC_009780.1
Cronobacter turicensis z3032	NC_013282.2; NC_013283.1; NC_013284.1; NC_013285.1
Cryptobacterium curtum DSM 15641	NC_013170.1
Cupriavidus metallidurans CH34	NC_007971.2; NC_007972.2; NC_007973.1; NC_007974.2
Cupriavidus necator N 1	NC_015723.1; NC_015724.1; NC_015726.1; NC_015727.1
Cupriavidus taiwanensis LMG 19424	NC_010528.1; NC_010529.1; NC_010530.1
cyanobacterium UCYN A	NC_013771.1
Cyanothece ATCC 51142	NC_010539.1; NC_010541.1; NC_010542.1; NC_010543.1; NC_010546.1; NC_010547.1
Cyanothece PCC 7424	NC_011729.1; NC_011730.1; NC_011732.1; NC_011733.1; NC_011734.1; NC_011737.1; NC_011738.1
Cyanothece PCC 7425	NC_011880.1; NC_011882.1; NC_011884.1; NC_011885.1
Cyanothece PCC 7822	NC_014501.1; NC_014502.1; NC_014503.1; NC_014504.1; NC_014533.1; NC_014534.1; NC_014535.1
Cyanothece PCC 8801	NC_011721.1; NC_011723.1; NC_011726.1; NC_011727.1
Cyanothece PCC 8802	NC_013160.1; NC_013161.1; NC_013163.1; NC_013167.1; NC_013168.1
Cyclobacterium marinum DSM 745	NC_015914.1
Cytophaga hutchinsonii ATCC 33406	NC_008255.1
Dechloromonas aromatica RCB	NC_007298.1
Deferribacter desulfuricans SSM1	NC_013939.1; NC_013940.1
Dehalococcoides BAV1	NC_009455.1
Dehalococcoides CBDB1	NC_007356.1
Dehalococcoides ethenogenes 195	NC_002936.3
Dehalococcoides GT	NC_013890.1

Dehalococcoides VS	NC_013552.1
Dehalogenimonas lykanthroporepellens BL DC 9	NC_014314.1
Deinococcus deserti VCD115	NC_012526.1; NC_012527.1; NC_012528.1; NC_012529.1
Deinococcus geothermalis DSM 11300	NC_008010.2; NC_008025.1; NC_009939.1
Deinococcus maricopensis DSM 21211	NC_014958.1
Deinococcus proteolyticus MRP	NC_015161.1; NC_015162.1; NC_015163.1; NC_015169.1; NC_015170.1
Deinococcus radiodurans R1	NC_000958.1; NC_000959.1; NC_001263.1; NC_001264.1
Delftia acidovorans SPH 1	NC_010002.1
Delftia Cs1 4	NC_015563.1
Denitrovibrio acetiphilus DSM 12809	NC_013943.1
Desulfarculus baarsii DSM 2075	NC_014365.1
Desulfatibacillum alkenivorans AK 01	NC_011768.1
Desulfitobacterium hafniense DCB 2	NC_011830.1
Desulfitobacterium hafniense Y51	NC_007907.1
Desulfobacca acetoxidans DSM 11109	NC_015388.1
Desulfobacterium autotrophicum HRM2	NC_012108.1; NC_012109.1
Desulfobulbus propionicus DSM 2032	NC_014972.1
Desulfococcus oleovorans Hxd3	NC_009943.1
Desulfohalobium retbaense DSM 5692	NC_013223.1; NC_013224.1
Desulfomicrobium baculatum DSM 4028	NC_013173.1
Desulfotalea psychrophila LSv54	NC_006138.1; NC_006139.1; NC_006140.1
Desulfotomaculum acetoxidans DSM 771	NC_013216.1
Desulfotomaculum carboxydivorans CO 1 SRB	NC_015565.1
Desulfotomaculum kuznetsovii DSM 6115	NC_015573.1
Desulfotomaculum reducens MI 1	NC_009253.1
Desulfotomaculum ruminis DSM 2154	NC_015589.1
Desulfovibrio aespoeensis Aspo 2	NC_014844.1
Desulfovibrio alaskensis G20	NC_007519.1
Desulfovibrio desulfuricans ATCC 27774	NC_011883.1
Desulfovibrio magneticus RS 1	NC_012795.1; NC_012796.1; NC_012797.1
Desulfovibrio salexigens DSM 2638	NC_012881.1
Desulfovibrio vulgaris DP4	NC_008741.1; NC_008751.1
Desulfovibrio vulgaris Hildenborough	NC_002937.3; NC_005863.1
Desulfovibrio vulgaris Miyazaki F	NC_011769.1
Desulfurispirillum indicum S5	NC_014836.1
Desulfurivibrio alkaliphilus AHT2	NC_014216.1
Desulfurobacterium thermolithotrophum DSM 11699	NC_015185.1
Desulfurococcus kamchatkensis 1221n	NC_011766.1
Desulfurococcus mucosus DSM 2162	NC_014961.1
Dichelobacter nodosus VCS1703A	NC_009446.1
Dickeya dadantii 3937	NC_014500.1
Dickeya dadantii Ech586	NC_013592.1
Dickeya dadantii Ech703	NC_012880.1
Dickeya zeae Ech1591	NC_012912.1
Dictyoglomus thermophilum H 6 12	NC_011297.1
Dictyoglomus turgidum DSM 6724	NC_011661.1
Dinoroseobacter shibae DFL 12	NC_009952.1; NC_009955.1; NC_009956.1; NC_009957.1; NC_009958.1; NC_009959.1
Dyadobacter fermentans DSM 18053	NC_013037.1
Edwardsiella ictaluri 93 146	NC_012779.1
Edwardsiella tarda EIB202	NC_013508.1; NC_013509.1
Eggerthella lenta DSM 2243	NC_013204.1
Eggerthella YY7918	NC_015738.1
Ehrlichia canis Jake	NC_007354.1
Ehrlichia chaffeensis Arkansas	NC_007799.1
Ehrlichia ruminantium Gardel	NC_006831.1
Ehrlichia ruminantium Welgevonden	NC_005295.2
Ehrlichia ruminantium Welgevonden	NC_006832.1

Elusimicrobium minutum Pei191	NC_010644.1
Enterobacter 638	NC_009425.1; NC_009436.1
Enterobacter aerogenes KCTC 2190	NC_015663.1
Enterobacter asburiae LF7a	NC_015963.1; NC_015968.1; NC_015969.1
Enterobacter cloacae ATCC 13047	NC_014107.1; NC_014108.1; NC_014121.1
Enterobacter cloacae SCF1	NC_014618.1
Enterococcus faecalis V583	NC_004668.1; NC_004669.1; NC_004670.1; NC_004671.1
Erwinia amylovora ATCC 49946	NC_013971.1; NC_013972.1; NC_013973.1
Erwinia amylovora CFBP1430	NC_013957.1; NC_013961.1
Erwinia billingiae Eb661	NC_014304.1; NC_014305.1; NC_014306.1
Erwinia pyrifoliae Ep1 96	NC_012214.1; NC_013263.1; NC_013264.1; NC_013265.1; NC_013954.1
Erwinia tasmaniensis Et1 99	NC_010693.1; NC_010694.1; NC_010695.1; NC_010696.1; NC_010697.1; NC_010699.1
Erysipelothrix rhusiopathiae Fujisawa	NC_015601.1
Erythrobacter litoralis HTCC2594	NC_007722.1
Escherichia coli 536	NC_008253.1
Escherichia coli 55989	NC_011748.1
Escherichia coli APEC O1	NC_008563.1; NC_009837.1; NC_009838.1
Escherichia coli ATCC 8739	NC_010468.1
Escherichia coli BL21 Gold DE3 pLysS AG	NC_012947.1
Escherichia coli B REL606	NC_012967.1
Escherichia coli BW2952	NC_012759.1
Escherichia coli CFT073	NC_004431.1
Escherichia coli E24377A	NC_009786.1; NC_009787.1; NC_009788.1; NC_009789.1; NC_009790.1; NC_009791.1; NC_009801.1
Escherichia coli ED1a	NC_011745.1
Escherichia coli HS	NC_009800.1
Escherichia coli IAI1	NC_011741.1
Escherichia coli IAI39	NC_011750.1
Escherichia coli K 12 substr DH10B	NC_010473.1
Escherichia coli K 12 substr MG1655	NC_000913.2
Escherichia coli O103 H2 12009	NC_013353.1; NC_013354.1
Escherichia coli O111 H 11128	NC_013364.1; NC_013365.1; NC_013366.1; NC_013367.1; NC_013368.1; NC_013370.1
Escherichia coli O127 H6 E2348 69	NC_011601.1; NC_011602.1; NC_011603.1
Escherichia coli O157 H7 EC4115	NC_011350.1; NC_011351.1; NC_011353.1
Escherichia coli O157 H7 EDL933	NC_002655.2; NC_007414.1
Escherichia coli O157 H7 Sakai	NC_002127.1; NC_002128.1; NC_002695.1
Escherichia coli O157 H7 TW14359	NC_013008.1; NC_013010.1
Escherichia coli O26 H11 11368	NC_013361.1; NC_013362.1; NC_013363.1; NC_013369.1; NC_014543.1
Escherichia coli O55 H7 CB9615	NC_013941.1; NC_013942.1
Escherichia coli S88	NC_011742.1; NC_011747.1
Escherichia coli SE11	NC_011407.1; NC_011408.1; NC_011411.1; NC_011413.1; NC_011415.1; NC_011416.1; NC_011419.1
Escherichia coli SMS 3 5	NC_010485.1; NC_010486.1; NC_010487.1; NC_010488.1; NC_010498.1
Escherichia coli UMN026	NC_011739.1; NC_011749.1; NC_011751.1
Escherichia coli UTI89	NC_007941.1; NC_007946.1
Escherichia fergusonii ATCC 35469	NC_011740.1; NC_011743.1
Ethanoligenens harbinense YUAN 3	NC_014828.1
Eubacterium eligens ATCC 27750	NC_012778.1; NC_012780.1; NC_012782.1
Eubacterium limosum KIST612	NC_014624.1
Eubacterium rectale ATCC 33656	NC_012781.1
Exiguobacterium AT1b	NC_012673.1
Exiguobacterium sibiricum 255 15	NC_010549.1; NC_010550.1; NC_010556.1
Ferrimonas balearica DSM 9799	NC_014541.1
Ferroglobus placidus DSM 10642	NC_013849.1
Fervidobacterium nodosum Rt17 B1	NC_009718.1
Fibrobacter succinogenes S85	NC_013410.1
Finegoldia magna ATCC 29328	NC_010371.1; NC_010376.1
Flavobacteriaceae bacterium 3519 10	NC_013062.1
Flavobacteriales bacterium HTCC2170	NC_014472.1

Flavobacterium branchiophilum	NC_016001.1
Flavobacterium johnsoniae UW101	NC_009441.1
Flavobacterium psychrophilum JIP02 86	NC_009613.1
Flexistipes sinusarabici DSM 4947	NC_015672.1
Fluviicola taffensis DSM 16823	NC_015321.1
Francisella novicida U112	NC_008601.1
Francisella philomiragia ATCC 25017	NC_010331.1; NC_010336.1
Francisella tularensis FSC198	NC_008245.1
Francisella tularensis holarctica FTNF002 00	NC_009749.1
Francisella tularensis holarctica LVS	NC_007880.1
Francisella tularensis holarctica OSU18	NC_008369.1
Francisella tularensis mediasiatica FSC147	NC_010677.1
Francisella tularensis SCHU S4	NC_006570.2
Francisella tularensis WY96 3418	NC_009257.1
Francisella TX077308	NC_015696.1
Frankia alni ACN14a	NC_008278.1
Frankia CcI3	NC_007777.1
Frankia EAN1pec	NC_009921.1
Frankia EuI1c	NC_014666.1
Frankia symbiont of Datisca glomerata	NC_015656.1; NC_015657.1; NC_015664.1
Fusobacterium nucleatum ATCC 25586	NC_003454.1
Gallibacterium anatis UMN179	NC_015460.1; NC_015461.1
Gallionella capsiferriformans ES 2	NC_014394.1
gamma proteobacterium HdN1	NC_014366.1
Gardnerella vaginalis 409 05	NC_013721.1
Gardnerella vaginalis ATCC 14019	NC_014644.1
Gemmatimonas aurantiaca T 27	NC_012489.1
Geobacillus C56 T3	NC_014206.1
Geobacillus kaustophilus HTA426	NC_006509.1; NC_006510.1
Geobacillus thermodenitrificans NG80 2	NC_009328.1; NC_009329.1
Geobacillus thermoglucosidasius C56 YS93	NC_015660.1; NC_015661.1; NC_015665.1
Geobacillus WCH70	NC_012790.1; NC_012793.1; NC_012794.1
Geobacillus Y412MC52	NC_014915.1; NC_014916.1
Geobacillus Y412MC61	NC_013411.1; NC_013412.1
Geobacillus Y4 1MC1	NC_014650.1; NC_014651.1
Geobacter bemidjiensis Bem	NC_011146.1
Geobacter FRC 32	NC_011979.1
Geobacter lovleyi SZ	NC_010814.1; NC_010815.1
Geobacter M18	NC_014973.1
Geobacter M21	NC_012918.1
Geobacter metallireducens GS 15	NC_007515.1; NC_007517.1
Geobacter sulfurreducens PCA	NC_002939.4
Geobacter uraniireducens Rf4	NC_009483.1
Geodermatophilus obscurus DSM 43160	NC_013757.1
Glaciecola 4H 3 7 YE 5	NC_015497.1; NC_015498.1
Glaciecola nitratireducens FR1064	NC_016041.1
Gloeobacter violaceus PCC 7421	NC_005125.1
Gluconacetobacter diazotrophicus PAl 5	NC_011365.1; NC_011367.1
Gluconacetobacter diazotrophicus PAl 5	NC_010123.1; NC_010124.1; NC_010125.1
Gluconacetobacter xylinus NBRC 3288	NC_016021.1; NC_016022.1; NC_016027.1; NC_016028.1; NC_016029.1; NC_016030.1; NC_016037.1
Gluconobacter oxydans 621H	NC_006672.1; NC_006673.1; NC_006674.1; NC_006675.1; NC_006676.1; NC_006677.1
Gordonia bronchialis DSM 43247	NC_013441.1; NC_013442.1
Gramella forsetii KT0803	NC_008571.1
Granulibacter bethesdensis CGDNIH1	NC_008343.1
Haemophilus ducreyi 35000HP	NC_002940.2
Haemophilus influenzae 86 028NP	NC_007146.2
Haemophilus influenzae F3031	NC_014920.1

Haemophilus influenzae F3047	NC_014922.1
Haemophilus influenzae PittEE	NC_009566.1
Haemophilus influenzae PittGG	NC_009567.1
Haemophilus influenzae Rd KW20	NC_000907.1
Haemophilus parainfluenzae T3T1	NC_015964.1
Haemophilus parasuis SH0165	NC_011852.1
Haemophilus somnus 129PT	NC_006298.1; NC_008309.1
Haemophilus somnus 2336	NC_010519.1
Hahella chejuensis KCTC 2396	NC_007645.1
Halalkalicoccus jeotgali B3	NC_014297.1; NC_014298.1; NC_014299.1; NC_014300.1; NC_014301.1; NC_014302.1; NC_014303.1
Halanaerobium hydrogeniformans	NC_014654.1
Haliangium ochraceum DSM 14365	NC_013440.1
Haliscomenobacter hydrossis DSM 1100	NC_015510.1; NC_015511.1; NC_015512.1; NC_015513.1
Haloarcula hispanica ATCC 33960	NC_015943.1; NC_015944.1; NC_015948.1
Haloarcula marismortui ATCC 43049	NC_006389.1; NC_006390.1; NC_006391.1; NC_006392.1; NC_006393.1;
NC_006394.1; NC_006395.1; NC_006396.1; NC_006397.1	
Halobacterium NRC 1	NC_001869.1; NC_002607.1; NC_002608.1
Halobacterium salinarum R1	NC_010364.1; NC_010366.1; NC_010367.1; NC_010368.1; NC_010369.1
Haloferax volcanii DS2	NC_013964.1; NC_013965.1; NC_013966.1; NC_013967.1; NC_013968.1
Halogeometricum borinquense DSM 11551	NC_014729.1; NC_014731.1; NC_014732.1; NC_014735.1; NC_014736.1; NC_014737.1
Halomicrobium mukohataei DSM 12286	NC_013201.1; NC_013202.1
Halomonas elongata DSM 2581	NC_014532.1
halophilic archaeon DL31	NC_015954.1; NC_015955.1; NC_015959.1
Halopiger xanaduensis SH 6	NC_015658.1; NC_015659.1; NC_015666.1; NC_015667.1
Haloquadratum walsbyi DSM 16790	NC_008212.1; NC_008213.1
Halorhabdus utahensis DSM 12940	NC_013158.1
Halorhodospira halophila SL1	NC_008789.1
Halorubrum lacusprofundi ATCC 49239	NC_012028.1; NC_012029.1; NC_012030.1
Haloterrigena turkmenica DSM 5511	NC_013743.1; NC_013744.1; NC_013745.1; NC_013746.1; NC_013747.1; NC_013748.1; NC_013749.1
Halothermothrix orenii H 168	NC_011899.1
Halothiobacillus neapolitanus c2	NC_013422.1
Helicobacter acinonychis Sheeba	NC_008229.1; NC_008230.1
Helicobacter bizzozeronii CIII 1	NC_015670.1; NC_015674.1
Helicobacter felis ATCC 49179	NC_014810.2
Helicobacter hepaticus ATCC 51449	NC_004917.1
Helicobacter mustelae 12198	NC_013949.1
Helicobacter pylori 26695	NC_000915.1
Helicobacter pylori B38	NC_012973.1
Helicobacter pylori B8	NC_014256.1; NC_014257.1
Helicobacter pylori G27	NC_011333.1; NC_011334.1
Helicobacter pylori HPAG1	NC_008086.1; NC_008087.1
Helicobacter pylori J99	NC_000921.1
Helicobacter pylori P12	NC_011498.1; NC_011499.1
Helicobacter pylori PeCan4	NC_014555.1; NC_014556.1
Helicobacter pylori Shi470	NC_010698.2
Helicobacter pylori SJM180	NC_014560.1
Heliobacterium modesticaldum Ice1	NC_010337.2
Herbaspirillum seropedicae SmR1	NC_014323.1
Herminiimonas arsenicoxydans	NC_009138.1
Herpetosiphon aurantiacus DSM 785	NC_009972.1; NC_009973.1; NC_009974.1
Hippea maritima DSM 10411	NC_015318.1
Hirschia baltica ATCC 49814	NC_012982.1; NC_012983.1
Hydrogenobacter thermophilus TK 6	NC_013799.1
Hydrogenobaculum Y04AAS1	NC_011126.1
Hyperthermus butylicus DSM 5456	NC_008818.1
Hyphomicrobium denitrificans ATCC 51888	NC_014313.1
Hyphomicrobium MC1	NC_015717.1

Hyphomonas neptunium ATCC 15444	NC_008358.1
Idiomarina loihiensis L2TR	NC_006512.1
Ignicoccus hospitalis KIN4 I	NC_009776.1
Ignisphaera aggregans DSM 17230	NC_014471.1
Ilyobacter polytropus DSM 2926	NC_014632.1; NC_014633.1; NC_014634.1
Intrasporangium calvum DSM 43043	NC_014830.1
Isoptericola variabilis 225	NC_015588.1
Isosphaera pallida ATCC 43644	NC_014957.1; NC_014962.1
Jannaschia CCS1	NC_007801.1; NC_007802.1
Janthinobacterium Marseille	NC_009659.1
Jonesia denitrificans DSM 20603	NC_013174.1
Kangiella koreensis DSM 16069	NC_013166.1
Ketogulonicigenium vulgare Y25	NC_014621.1; NC_014625.1; NC_014626.1
Kineococcus radiotolerans SRS30216	NC_009660.1; NC_009664.2; NC_009806.1
Kitasatospora setae KM 6054	NC_016109.1
Klebsiella pneumoniae 342	NC_011281.1; NC_011282.1; NC_011283.1
Klebsiella pneumoniae MGH 78578	NC_009648.1; NC_009649.1; NC_009650.1; NC_009651.1; NC_009652.1; NC_009653.1
Klebsiella pneumoniae NTUH K2044	NC_006625.1; NC_012731.1
Klebsiella variicola At 22	NC_013850.1
Kocuria rhizophila DC2201	NC_010617.1
Kosmotoga olearia TBF 19 5 1	NC_012785.1
Kribbella flavida DSM 17836	NC_013729.1
Krokinobacter 4H 3 7 5	NC_015496.1
Kytococcus sedentarius DSM 20547	NC_013169.1
Lacinutrix 5H 3 7 4	NC_015638.1
Lactobacillus acidophilus 30SC	NC_015213.1; NC_015214.1; NC_015218.1
Lactobacillus acidophilus NCFM	NC_006814.3
Lactobacillus amylovorus GRL 1112	NC_014724.1; NC_015319.1; NC_015322.1
Lactobacillus brevis ATCC 367	NC_008497.1; NC_008498.1; NC_008499.1
Lactobacillus buchneri NRRL B 30929	NC_015420.1; NC_015421.1; NC_015428.1; NC_015429.1
Lactobacillus casei ATCC 334	NC_008502.1; NC_008526.1
Lactobacillus casei BL23	NC_010999.1
Lactobacillus casei Zhang	NC_011352.1; NC_014334.1
Lactobacillus crispatus ST1	NC_014106.1
Lactobacillus delbrueckii bulgaricus ATCC 11842	NC_008054.1
Lactobacillus delbrueckii bulgaricus ATCC BAA 365	NC_008529.1
Lactobacillus delbrueckii bulgaricus ND02	NC_014727.1; NC_014728.1
Lactobacillus fermentum IFO 3956	NC_010610.1
Lactobacillus gasseri ATCC 33323	NC_008530.1
Lactobacillus helveticus DPC 4571	NC_010080.1
Lactobacillus johnsonii FI9785	NC_012552.1; NC_013504.1; NC_013505.1
Lactobacillus johnsonii NCC 533	NC_005362.1
Lactobacillus kefiranofaciens ZW3	NC_015598.1; NC_015602.1; NC_015603.1
Lactobacillus plantarum JDM1	NC_012984.1
Lactobacillus plantarum ST III	NC_014554.1; NC_014558.2
Lactobacillus plantarum WCFS1	NC_004567.1; NC_006375.1; NC_006376.1; NC_006377.1
Lactobacillus reuteri DSM 20016	NC_009513.1
Lactobacillus reuteri JCM 1112	NC_010609.1
Lactobacillus reuteri SD2112	NC_015697.1; NC_015698.1; NC_015699.1; NC_015700.1; NC_015701.1
Lactobacillus rhamnosus GG	NC_013198.1
Lactobacillus rhamnosus Lc 705	NC_013199.1; NC_013200.1
Lactobacillus ruminis ATCC 27782	NC_015975.1
Lactobacillus sakei 23K	NC_007576.1
Lactobacillus salivarius UCC118	NC_006529.1; NC_006530.1; NC_007929.1; NC_007930.1
Lactobacillus sanfranciscensis TMW 1 1304	NC_015978.1; NC_015979.1; NC_015980.1
Lactococcus garvieae ATCC 49156	NC_015930.1
Lactococcus lactis cremoris MG1363	NC_009004.1

Lactococcus lactis cremoris SK11	NC_008503.1; NC_008504.1; NC_008505.1; NC_008506.1; NC_008507.1; NC_008527.1
Lactococcus lactis Il1403	NC_002662.1
Lactococcus lactis KF147	NC_013656.1; NC_013657.1
Laribacter hongkongensis HLHK9	NC_012559.1
Lawsonia intracellularis PHE MN1 00	NC_008011.1; NC_008012.1; NC_008013.1; NC_008014.1
Leadbetterella byssophila DSM 17132	NC_014655.1
Legionella longbeachae NSW150	NC_013861.1; NC_014544.1
Legionella pneumophila 2300 99 Alcoy	NC_014125.1
Legionella pneumophila Corby	NC_009494.2
Legionella pneumophila Lens	NC_006366.1; NC_006369.1
Legionella pneumophila Paris	NC_006365.1; NC_006368.1
Legionella pneumophila Philadelphia 1	NC_002942.5
Leifsonia xyli CTCB07	NC_006087.1
Leptospira biflexa serovar Patoc Patoc 1 Ames	NC_010842.1; NC_010845.1; NC_010846.1
Leptospira biflexa serovar Patoc Patoc 1 Paris	NC_010602.1; NC_010843.1; NC_010844.1
Leptospira borgpetersenii serovar Hardjo bovis JB197	NC_008510.1; NC_008511.1
Leptospira borgpetersenii serovar Hardjo bovis L550	NC_008508.1; NC_008509.1
Leptospira interrogans serovar Copenhageni Fiocruz L1 130	NC_005823.1; NC_005824.1
Leptospira interrogans serovar Lai 56601	NC_004342.2; NC_004343.2
Leptothrix cholodnii SP 6	NC_010524.1
Leptotrichia buccalis C 1013 b	NC_013192.1
Leuconostoc C2	NC_015734.1
Leuconostoc citreum KM20	NC_010466.1; NC_010467.1; NC_010469.1; NC_010470.1; NC_010471.1
Leuconostoc gasicomitatum LMG 18811	NC_014319.1
Leuconostoc kimchii IMSNU 11154	NC_014131.1; NC_014132.1; NC_014133.1; NC_014134.1; NC_014135.1; NC_014136.1
Leuconostoc mesenteroides ATCC 8293	NC_008496.1; NC_008531.1
Listeria innocua Clip11262	NC_003212.1; NC_003383.1
Listeria ivanovii PAM 55	NC_016011.1
Listeria monocytogenes 08 5923	NC_013768.1
Listeria monocytogenes Clip80459	NC_012488.1
Listeria monocytogenes EGD e	NC_003210.1
Listeria monocytogenes HCC23	NC_011660.1
Listeria monocytogenes serotype 4b F2365	NC_002973.6
Listeria monocytogenes	NC_013766.1; NC_013767.1
Listeria seeligeri serovar 1 2b SLCC3954	NC_013891.1
Listeria welshimeri serovar 6b SLCC5334	NC_008555.1
Lysinibacillus sphaericus C3 41	NC_010381.1; NC_010382.1
Macrococcus caseolyticus JCSC5402	NC_011995.1; NC_011996.1; NC_011997.1; NC_011998.1; NC_011999.1; NC_012000.1; NC_012001.1; NC_012002.1; NC_012003.1
Magnetococcus MC 1	NC_008576.1
Magnetospirillum magneticum AMB 1	NC_007626.1
Mahella australiensis 50 1 BON	NC_015520.1
Mannheimia succiniciproducens MBEL55E	NC_006300.1
Maricaulis maris MCS10	NC_008347.1
Marinithermus hydrothermalis DSM 14884	NC_015387.1
Marinobacter aquaeolei VT8	NC_008738.1; NC_008739.1; NC_008740.1
Marinomonas mediterranea MMB 1	NC_015276.1
Marinomonas MWYL1	NC_009654.1
Marinomonas posidonica IVIA Po 181	NC_015559.1
Marivirga tractuosa DSM 4126	NC_014750.1; NC_014759.1
Megasphaera elsdenii DSM 20460	NC_015873.1
Meiothermus ruber DSM 1279	NC_013946.1
Meiothermus silvanus DSM 9946	NC_014212.1; NC_014213.1; NC_014214.1
Melissococcus plutonius ATCC 35311	NC_015516.1; NC_015517.1
Mesoplasma florum L1	NC_006055.1
Mesorhizobium ciceri biovar biserrulae WSM1271	NC_014918.1; NC_014923.1
Mesorhizobium loti MAFF303099	NC_002678.2; NC_002679.1; NC_002682.1

Mesorhizobium opportunistum WSM2075	NC_015675.1
Metallosphaera cuprina Ar 4	NC_015435.1
Metallosphaera sedula DSM 5348	NC_009440.1
Methanobacterium AL 21	NC_015216.1
Methanobacterium SWAN 1	NC_015574.1
Methanobrevibacter ruminantium M1	NC_013790.1
Methanobrevibacter smithii ATCC 35061	NC_009515.1
Methanocaldococcus fervens AG86	NC_013156.1; NC_013157.1
Methanocaldococcus FS406 22	NC_013887.1; NC_013888.1
Methanocaldococcus infernus ME	NC_014122.1
Methanocaldococcus jannaschii DSM 2661	NC_000909.1; NC_001732.1; NC_001733.1
Methanocaldococcus vulcanius M7	NC_013407.1; NC_013408.1; NC_013409.1
Methanocella paludicola SANAE	NC_013665.1
Methanococcoides burtonii DSM 6242	NC_007955.1
Methanococcus aeolicus Nankai 3	NC_009635.1
Methanococcus maripaludis C5	NC_009135.1; NC_009136.1
Methanococcus maripaludis C6	NC_009975.1
Methanococcus maripaludis C7	NC_009637.1
Methanococcus maripaludis S2	NC_005791.1
Methanococcus maripaludis X1	NC_015847.1
Methanococcus maripaludis XI	NC_015847.1
Methanococcus vannielii SB	NC_009634.1
Methanococcus voltae A3	NC_014222.1
Methanocorpusculum labreanum Z	NC_008942.1
Methanoculleus marisnigri JR1	NC_009051.1
Methanohalobium evestigatum Z 7303	NC_014253.1; NC_014254.1
Methanohalophilus mahii DSM 5219	NC_014002.1
Methanoplanus petrolearius DSM 11571	NC_014507.1
Methanopyrus kandleri AV19	NC_003551.1
Methanoregula boonei 6A8	NC_009712.1
Methanosaeta concilii GP6	NC_015416.1; NC_015430.1
Methanosaeta thermophila PT	NC_008553.1
Methanosalsum zhilinae DSM 4017	NC_015676.1
Methanosarcina acetivorans C2A	NC_003552.1
Methanosarcina barkeri Fusaro	NC_007349.1; NC_007355.1
Methanosarcina mazei Go1	NC_003901.1
Methanosphaera stadtmanae DSM 3091	NC_007681.1
Methanosphaerula palustris E1 9c	NC_011832.1
Methanospirillum hungatei JF 1	NC_007796.1
Methanothermobacter marburgensis Marburg	NC_014408.1; NC_014409.1
Methanothermobacter thermautotrophicus Delta H	NC_000916.1
Methanothermococcus okinawensis IH1	NC_015632.1; NC_015636.1
Methanothermus fervidus DSM 2088	NC_014658.1
Methanotorris igneus Kol 5	NC_015562.1
Methylacidiphilum infernorum V4	NC_010794.1
Methylibium petroleiphilum PM1	NC_008825.1; NC_008826.1
Methylobacillus flagellatus KT	NC_007947.1
Methylobacterium 4 46	NC_010373.1; NC_010374.1; NC_010511.1
Methylobacterium chloromethanicum CM4	NC_011757.1; NC_011758.1; NC_011760.1
Methylobacterium extorquens AM1	NC_012807.1; NC_012808.1; NC_012809.1; NC_012810.1; NC_012811.1
Methylobacterium extorquens DM4	NC_012987.1; NC_012988.1; NC_012989.1
Methylobacterium extorquens PA1	NC_010172.1
Methylobacterium nodulans ORS 2060	NC_011887.1; NC_011888.1; NC_011889.1;
	NC_011890.1; NC_011892.1; NC_011893.1; NC_011894.1; NC_011895.1
Methylobacterium populi BJ001	NC_010721.1; NC_010725.1; NC_010727.1
Methylobacterium radiotolerans JCM 2831	NC_010502.1; NC_010504.1; NC_010505.1; NC_010507.1;
	NC_010509.1; NC_010510.1; NC_010514.1; NC_010517.1; NC_010518.1

Methylocella silvestris BL2	NC_011666.1
Methylococcus capsulatus Bath	NC_002977.6
Methylomicrobium alcaliphilum	NC_016112.1
Methylomonas methanica MC09	NC_015572.1
Methylotenera 301	NC_014207.1
Methylotenera mobilis JLW8	NC_012968.1
Methylovorus glucosetrophus SIP3 4	NC_012969.1; NC_012970.1; NC_012972.1
Methylovorus MP688	NC_014733.1
Micavibrio aeruginosavorus ARL 13	NC_016026.1
Microbacterium testaceum StLB037	NC_015125.1
Micrococcus luteus NCTC 2665	NC_012803.1
Microcystis aeruginosa NIES 843	NC_010296.1
Microlunatus phosphovorus NM 1	NC_015635.1
Micromonospora aurantiaca ATCC 27029	NC_014391.1
Micromonospora L5	NC_014815.1
Mobiluncus curtisii ATCC 43063	NC_014246.1
Moorella thermoacetica ATCC 39073	NC_007644.1
Moraxella catarrhalis RH4	NC_014147.1
Muricauda ruestringensis DSM 13258	NC_015945.1
Mycobacterium abscessus ATCC 19977	NC_010394.1; NC_010397.1
Mycobacterium africanum GM041182	NC_015758.1
Mycobacterium avium 104	NC_008595.1
Mycobacterium avium paratuberculosis K 10	NC_002944.2
Mycobacterium bovis AF2122 97	NC_002945.3
Mycobacterium bovis BCG Pasteur 1173P2	NC_008769.1
Mycobacterium bovis BCG Tokyo 172	NC_012207.1
Mycobacterium canettii	NC_015848.1
Mycobacterium gilvum PYR GCK	NC_009338.1; NC_009339.1; NC_009340.1; NC_009341.1
Mycobacterium JDM601	NC_015576.1
Mycobacterium JLS	NC_009077.1
Mycobacterium KMS	NC_008703.1; NC_008704.1; NC_008705.1
Mycobacterium leprae Br4923	NC_011896.1
Mycobacterium leprae TN	NC_002677.1
Mycobacterium marinum M	NC_010604.1; NC_010612.1
Mycobacterium MCS	NC_008146.1; NC_008147.1
Mycobacterium smegmatis MC2 155	NC_008596.1
Mycobacterium Spyr1	NC_014811.1; NC_014812.1; NC_014814.1
Mycobacterium tuberculosis CDC1551	NC_002755.2
Mycobacterium tuberculosis F11	NC_009565.1
Mycobacterium tuberculosis H37Ra	NC_009525.1
Mycobacterium tuberculosis H37Rv	NC_000962.2
Mycobacterium tuberculosis KZN 1435	NC_012943.1
Mycobacterium ulcerans Agy99	NC_005916.1; NC_008611.1
Mycobacterium vanbaalenii PYR 1	NC_008726.1
Mycoplasma agalactiae PG2	NC_009497.1
Mycoplasma agalactiae	NC_013948.1
Mycoplasma arthritidis 158L3 1	NC_011025.1
Mycoplasma bovis Hubei 1	NC_015725.1
Mycoplasma bovis PG45	NC_014760.1
Mycoplasma capricolum ATCC 27343	NC_007633.1
Mycoplasma conjunctivae HRC 581	NC_012806.1
Mycoplasma crocodyli MP145	NC_014014.1
Mycoplasma fermentans JER	NC_014552.1
Mycoplasma fermentans M64	NC_014921.1
Mycoplasma gallisepticum R low	NC_004829.2
Mycoplasma genitalium G37	NC_000908.2
Mycoplasma haemofelis Langford 1	NC_014970.1

Mycoplasma hominis ATCC 23114	NC_013511.1
Mycoplasma hyopneumoniae 232	NC_006360.1
Mycoplasma hyopneumoniae 7448	NC_007332.1
Mycoplasma hyopneumoniae J	NC_007295.1
Mycoplasma hyorhinis HUB 1	NC_014448.1
Mycoplasma leachii PG50	NC_014751.1
Mycoplasma mobile 163K	NC_006908.1
Mycoplasma mycoides capri LC 95010	NC_015407.1; NC_015431.1
Mycoplasma mycoides SC PG1	NC_005364.2
Mycoplasma penetrans HF 2	NC_004432.1
Mycoplasma pneumoniae M129	NC_000912.1
Mycoplasma pulmonis UAB CTIP	NC_002771.1
Mycoplasma putrefaciens KS1	NC_015946.1
Mycoplasma suis Illinois	NC_015155.1
Mycoplasma suis KI3806	NC_015153.1
Mycoplasma synoviae 53	NC_007294.1
Myxococcus fulvus HW 1	NC_015711.1
Myxococcus xanthus DK 1622	NC_008095.1
Nakamurella multipartita DSM 44233	NC_013235.1
Nanoarchaeum equitans Kin4 M	NC_005213.1
Natranaerobius thermophilus JW NM WN LF	NC_010715.1; NC_010718.1; NC_010724.1
Natrialba magadii ATCC 43099	NC_013922.1; NC_013923.1; NC_013924.1; NC_013925.1
Natronomonas pharaonis DSM 2160	NC_007426.1; NC_007427.1; NC_007428.1
Nautilia profundicola AmH	NC_012115.1
Neisseria gonorrhoeae FA 1090	NC_002946.2
Neisseria gonorrhoeae NCCP11945	NC_011034.1; NC_011035.1
Neisseria lactamica 020 06	NC_014752.1
Neisseria meningitidis 053442	NC_010120.1
Neisseria meningitidis alpha14	NC_013016.1
Neisseria meningitidis FAM18	NC_008767.1
Neisseria meningitidis MC58	NC_003112.2
Neisseria meningitidis Z2491	NC_003116.1
Neorickettsia risticii Illinois	NC_013009.1
Neorickettsia sennetsu Miyayama	NC_007798.1
Nitratifractor salsuginis DSM 16511	NC_014935.1
Nitratiruptor SB155 2	NC_009662.1
Nitrobacter hamburgensis X14	NC_007959.1; NC_007960.1; NC_007961.1; NC_007964.1
Nitrobacter winogradskyi Nb 255	NC_007406.1
Nitrosococcus halophilus Nc4	NC_013958.1; NC_013960.1
Nitrosococcus oceani ATCC 19707	NC_007483.1; NC_007484.1
Nitrosococcus watsonii C 113	NC_014315.1; NC_014316.1; NC_014317.1
Nitrosomonas AL212	NC_015221.1; NC_015222.1; NC_015223.1
Nitrosomonas europaea ATCC 19718	NC_004757.1
Nitrosomonas eutropha C91	NC_008341.1; NC_008342.1; NC_008344.1
Nitrosomonas Is79A3	NC_015731.1
Nitrosopumilus maritimus SCM1	NC_010085.1
Nitrosospira multiformis ATCC 25196	NC_007614.1; NC_007615.1; NC_007616.1; NC_007617.1
Nocardia farcinica IFM 10152	NC_006361.1; NC_006362.1; NC_006363.1
Nocardioides JS614	NC_008697.1; NC_008699.1
Nocardiopsis dassonvillei DSM 43111	NC_014210.1; NC_014211.1
Nostoc azollae 0708	NC_014248.1; NC_014249.1; NC_014250.1
Nostoc PCC 7120	NC_003240.1; NC_003241.1; NC_003267.1; NC_003270.1; NC_003272.1; NC_003273.1; NC_003276.1
Nostoc punctiforme PCC 73102	NC_010628.1; NC_010629.1; NC_010630.1; NC_010631.1; NC_010632.1; NC_010633.1
Novosphingobium aromaticivorans DSM 12444	NC_007794.1; NC_009426.1; NC_009427.1
Novosphingobium PP1Y	NC_015579.1; NC_015580.1; NC_015582.1; NC_015583.1
Oceanithermus profundus DSM 14977	NC_014753.1; NC_014761.1
Oceanobacillus iheyensis HTE831	NC_004193.1

Ochrobactrum anthropi ATCC 49188	NC_009667.1; NC_009668.1; NC_009669.1; NC_009670.1; NC_009671.1; NC_009672.1
Odoribacter splanchnicus DSM 20712	NC_015160.1
Oenococcus oeni PSU 1	NC_008528.1
Oligotropha carboxidovorans OM5	NC_011386.1
Oligotropha carboxidovorans OM5	NC_015684.1; NC_015685.1; NC_015689.1
Olsenella uli DSM 7084	NC_014363.1
Onion yellows phytoplasma OY M	NC_005303.2
Opitutus terrae PB90 1	NC_010571.1
Orientia tsutsugamushi Boryong	NC_009488.1
Orientia tsutsugamushi Ikeda	NC_010793.1
Oscillibacter valericigenes	NC_016046.1; NC_016048.1
Paenibacillus JDR 2	NC_012914.1
Paenibacillus mucilaginosus KNP414	NC_015690.1
Paenibacillus polymyxa E681	NC_014483.1
Paenibacillus polymyxa SC2	NC_014622.1; NC_014628.1
Paenibacillus Y412MC10	NC_013406.1
Paludibacter propionicigenes WB4	NC_014734.1
Pantoea ananatis LMG 20103	NC_013956.2
Pantoea At 9b	NC_014837.1; NC_014838.1; NC_014839.1; NC_014840.1; NC_014841.1; NC_014842.1
Pantoea vagans C9 1	NC_014258.1; NC_014561.1; NC_014562.1; NC_014563.1
Parabacteroides distasonis ATCC 8503	NC_009615.1
Parachlamydia acanthamoebae UV7	NC_015702.1
Paracoccus denitrificans PD1222	NC_008686.1; NC_008687.1; NC_008688.1
Parvibaculum lavamentivorans DS 1	NC_009719.1
Parvularcula bermudensis HTCC2503	NC_014414.1
Pasteurella multocida Pm70	NC_002663.1
Pectobacterium atrosepticum SCRI1043	NC_004547.2
Pectobacterium carotovorum PC1	NC_012917.1
Pectobacterium wasabiae WPP163	NC_013421.1
Pediococcus pentosaceus ATCC 25745	NC_008525.1
Pedobacter heparinus DSM 2366	NC_013061.1
Pedobacter saltans DSM 12145	NC_015177.1
Pelagibacterium halotolerans B2	NC_016078.1; NC_016079.1
Pelobacter carbinolicus DSM 2380	NC_007498.2
Pelobacter propionicus DSM 2379	NC_008607.1; NC_008608.1; NC_008609.1
Pelodictyon phaeoclathratiforme BU 1	NC_011060.1
Pelotomaculum thermopropionicum SI	NC_009454.1
Persephonella marina EX H1	NC_012439.1; NC_012440.1
Petrotoga mobilis SJ95	NC_010003.1
Phenylobacterium zucineum HLK1	NC_011143.1; NC_011144.1
Photobacterium profundum SS9	NC_005871.1; NC_006370.1; NC_006371.1
Photorhabdus asymbiotica ATCC 43949	NC_012961.1; NC_012962.1
Photorhabdus luminescens laumondii TTO1	NC_005126.1
Picrophilus torridus DSM 9790	NC_005877.1
Pirellula staleyi DSM 6068	NC_013720.1
Planctomyces brasiliensis DSM 5305	NC_015174.1
Planctomyces limnophilus DSM 3776	NC_014148.1; NC_014149.1
Polaromonas JS666	NC_007948.1; NC_007949.1; NC_007950.1
Polaromonas naphthalenivorans CJ2	NC_008757.1; NC_008758.1; NC_008759.1;
	NC_008760.1; NC_008761.1; NC_008762.1; NC_008763.1; NC_008764.1; NC_008781.1
Polymorphum gilvum SL003B 26A1	NC_015258.1; NC_015259.1
Polynucleobacter necessarius asymbioticus QLW P1DMWA 1	NC_009379.1
Polynucleobacter necessarius STIR1	NC_010531.1
Porphyromonas asaccharolytica DSM 20707	NC_015501.1
Porphyromonas gingivalis ATCC 33277	NC_010729.1
Porphyromonas gingivalis TDC60	NC_015571.1
Porphyromonas gingivalis W83	NC_002950.2

Prevotella denticola F0289	NC_015311.1
Prevotella melaninogenica ATCC 25845	NC_014370.1; NC_014371.1
Prevotella ruminicola 23	NC_014033.1
Prochlorococcus marinus AS9601	NC_008816.1
Prochlorococcus marinus CCMP1375	NC_005042.1
Prochlorococcus marinus MIT 9211	NC_009976.1
Prochlorococcus marinus MIT 9215	NC_009840.1
Prochlorococcus marinus MIT 9301	NC_009091.1
Prochlorococcus marinus MIT 9303	NC_008820.1
Prochlorococcus marinus MIT 9312	NC_007577.1
Prochlorococcus marinus MIT 9313	NC_005071.1
Prochlorococcus marinus MIT 9515	NC_008817.1
Prochlorococcus marinus NATL1A	NC_008819.1
Prochlorococcus marinus NATL2A	NC_007335.2
Prochlorococcus marinus pastoris CCMP1986	NC_005072.1
Propionibacterium acnes KPA171202	NC_006085.1
Propionibacterium acnes SK137	NC_014039.1
Propionibacterium freudenreichii shermanii CIRM BIA1	NC_014215.1
Prosthecochloris aestuarii DSM 271	NC_011059.1; NC_011061.1
Proteus mirabilis HI4320	NC_010554.1; NC_010555.1
Pseudoalteromonas atlantica T6c	NC_008228.1
Pseudoalteromonas haloplanktis TAC125	NC_007481.1; NC_007482.1
Pseudoalteromonas SM9913	NC_014800.1; NC_014803.1
Pseudogulbenkiania NH8B	NC_016002.1
Pseudomonas aeruginosa LESB58	NC_011770.1
Pseudomonas aeruginosa PA7	NC_009656.1
Pseudomonas aeruginosa PAO1	NC_002516.2
Pseudomonas aeruginosa UCBPP PA14	NC_008463.1
Pseudomonas brassicacearum NFM421	NC_015379.1
Pseudomonas entomophila L48	NC_008027.1
Pseudomonas fluorescens Pf0 1	NC_007492.2
Pseudomonas fluorescens Pf 5	NC_004129.6
Pseudomonas fluorescens SBW25	NC_009444.1; NC_012660.1
Pseudomonas fulva 12 X	NC_015556.1
Pseudomonas mendocina NK 01	NC_015410.1
Pseudomonas mendocina ymp	NC_009439.1
Pseudomonas putida F1	NC_009512.1
Pseudomonas putida GB 1	NC_010322.1
Pseudomonas putida KT2440	NC_002947.3
Pseudomonas putida S16	NC_015733.1
Pseudomonas putida W619	NC_010501.1
Pseudomonas stutzeri A1501	NC_009434.1
Pseudomonas stutzeri ATCC 17588 LMG 11199	NC_015740.1
Pseudomonas syringae B728a	NC_007005.1
Pseudomonas syringae phaseolicola 1448A	NC_005773.3; NC_007274.1; NC_007275.1
Pseudomonas syringae tomato DC3000	NC_004578.1; NC_004632.1; NC_004633.1
Pseudonocardia dioxanivorans CB1190	NC_015312.1; NC_015313.1; NC_015314.1
Pseudoxanthomonas spadix BD a59	NC_016147.1
Pseudoxanthomonas suwonensis 11 1	NC_014924.1
Psychrobacter arcticus 273 4	NC_007204.1
Psychrobacter cryohalolentis K5	NC_007968.1; NC_007969.1
Psychrobacter PRwf 1	NC_009516.1; NC_009517.1; NC_009524.1
Psychromonas ingrahamii 37	NC_008709.1
Pusillimonas T7 7	NC_015458.1; NC_015459.1
Pyrobaculum aerophilum IM2	NC_003364.1
Pyrobaculum arsenaticum DSM 13514	NC_009376.1
Pyrobaculum calidifontis JCM 11548	NC_009073.1

Pyrobaculum islandicum DSM 4184	NC_008701.1
Pyrococcus abyssi GE5	NC_000868.1; NC_001773.1
Pyrococcus furiosus DSM 3638	NC_003413.1
Pyrococcus horikoshii OT3	NC_000961.1
Pyrococcus NA2	NC_015474.1
Pyrococcus yayanosii CH1	NC_015680.1
Pyrolobus fumarii 1A	NC_015931.1
Rahnella Y9602	NC_015061.1; NC_015062.1; NC_015063.1
Ralstonia eutropha H16	NC_005241.1; NC_008313.1; NC_008314.1
Ralstonia eutropha JMP134	NC_007336.1; NC_007337.1; NC_007347.1; NC_007348.1
Ralstonia pickettii 12D	NC_012849.1; NC_012851.1; NC_012855.1; NC_012856.1; NC_012857.1
Ralstonia pickettii 12J	NC_010678.1; NC_010682.1; NC_010683.1
Ralstonia solanacearum CFBP2957	NC_014307.1
Ralstonia solanacearum GMI1000	NC_003295.1; NC_003296.1
Ralstonia solanacearum PSI07	NC_014310.1; NC_014311.1
Ramlibacter tataouinensis TTB310	NC_015677.1
Renibacterium salmoninarum ATCC 33209	NC_010168.1
Rhizobium etli CFN 42	NC_004041.2; NC_007761.1; NC_007762.1; NC_007763.1; NC_007764.1; NC_007765.1; NC_007766.1
Rhizobium etli CIAT 652	NC_010994.1; NC_010996.1; NC_010997.1; NC_010998.1
Rhizobium leguminosarum bv trifolii WSM1325	NC_012848.1; NC_012850.1; NC_012852.1; NC_012853.1; NC_012854.1; NC_012858.1
Rhizobium leguminosarum bv trifolii WSM2304	NC_011366.1; NC_011368.1; NC_011369.1; NC_011370.1; NC_011371.1
Rhizobium leguminosarum bv viciae 3841	NC_008378.1; NC_008379.1; NC_008380.1; NC_008381.1; NC_008382.1; NC_008383.1; NC_008384.1
Rhizobium NGR234	NC_000914.2; NC_012586.1; NC_012587.1
Rhodobacter capsulatus SB 1003	NC_014034.1; NC_014035.1
Rhodobacter sphaeroides 2 4 1	NC_007488.1; NC_007489.1; NC_007490.1; NC_007493.1; NC_007494.1; NC_009007.1; NC_009008.1
Rhodobacter sphaeroides ATCC 17025	NC_009428.1; NC_009429.1; NC_009430.1; NC_009431.1; NC_009432.1; NC_009433.1
Rhodobacter sphaeroides ATCC 17029	NC_009040.1; NC_009049.1; NC_009050.1
Rhodobacter sphaeroides KD131	NC_011958.1; NC_011960.1; NC_011962.1; NC_011963.1
Rhodococcus equi 103S	NC_014659.1
Rhodococcus erythropolis PR4	NC_007486.1; NC_007487.1; NC_007491.1; NC_012490.1
Rhodococcus jostii RHA1	NC_008268.1; NC_008269.1; NC_008270.1; NC_008271.1
Rhodococcus opacus B4	NC_006969.2; NC_006970.2; NC_012520.1; NC_012521.1; NC_012522.1; NC_012523.1
Rhodoferax ferrireducens T118	NC_007901.1; NC_007908.1
Rhodomicrobium vannielii ATCC 17100	NC_014664.1
Rhodopirellula baltica SH 1	NC_005027.1
Rhodopseudomonas palustris BisA53	NC_008435.1
Rhodopseudomonas palustris BisB18	NC_007925.1
Rhodopseudomonas palustris BisB5	NC_007958.1
Rhodopseudomonas palustris CGA009	NC_005296.1; NC_005297.1
Rhodopseudomonas palustris DX 1	NC_014834.1
Rhodopseudomonas palustris HaA2	NC_007778.1
Rhodopseudomonas palustris TIE 1	NC_011004.1
Rhodospirillum centenum SW	NC_011420.2
Rhodospirillum rubrum ATCC 11170	NC_007641.1; NC_007643.1
Rhodothermus marinus DSM 4252	NC_013501.1; NC_013502.1
Rhodothermus marinus SG0 5JP17 172	NC_015966.1; NC_015967.1; NC_015970.1
Rickettsia africae ESF 5	NC_012633.1; NC_012634.1
Rickettsia akari Hartford	NC_009881.1
Rickettsia bellii OSU 85 389	NC_009883.1
Rickettsia bellii RML369 C	NC_007940.1
Rickettsia canadensis McKiel	NC_009879.1
Rickettsia conorii Malish 7	NC_003103.1
Rickettsia felis URRWXCal2	NC_007109.1; NC_007110.1; NC_007111.1
Rickettsia heilongjiangensis 054	NC_015866.1
Rickettsia japonica YH	NC_016050.1
Rickettsia massiliae MTU5	NC_009897.1; NC_009900.1
Rickettsia peacockii Rustic	NC_012730.1; NC_012732.1

Rickettsia prowazekii Madrid E	NC_000963.1
Rickettsia rickettsii Iowa	NC_010263.2
Rickettsia rickettsii Sheila Smith	NC_009882.1
Rickettsia typhi Wilmington	NC_006142.1
Riemerella anatipestifer DSM 15868	NC_014738.1
Robiginitalea biformata HTCC2501	NC_013222.1
Roseburia hominis A2 183	NC_015977.1
Roseiflexus castenholzii DSM 13941	NC_009767.1
Roseiflexus RS 1	NC_009523.1
Roseobacter denitrificans OCh 114	NC_008209.1; NC_008386.1; NC_008387.1; NC_008388.1; NC_008389.1
Roseobacter litoralis Och 149	NC_015728.1; NC_015729.1; NC_015730.1; NC_015741.1
Rothia dentocariosa ATCC 17931	NC_014643.1
Rothia mucilaginosa	NC_013715.1
Rubrobacter xylanophilus DSM 9941	NC_008148.1
Ruegeria pomeroyi DSS 3	NC_003911.11; NC_006569.1
Ruegeria TM1040	NC_008042.1; NC_008043.1; NC_008044.1
Ruminococcus albus 7	NC_014824.1; NC_014825.1; NC_014826.1; NC_014827.1; NC_014833.1
Runella slithyformis DSM 19594	NC_015693.1; NC_015694.1; NC_015695.1; NC_015703.1; NC_015704.1; NC_015705.1
Saccharomonospora viridis DSM 43017	NC_013159.1
Saccharophagus degradans 2 40	NC_007912.1
Saccharopolyspora erythraea NRRL 2338	NC_009142.1
Salinibacter ruber DSM 13855	NC_007677.1; NC_007678.1
Salinibacter ruber M8	NC_014026.1; NC_014028.1; NC_014030.1; NC_014032.1
Salinispora arenicola CNS 205	NC_009953.1
Salinispora tropica CNB 440	NC_009380.1
Salmonella bongori NCTC 12419	NC_015761.1
Salmonella enterica arizonae serovar 62 z4 z23 RSK2980	NC_010067.1
Salmonella enterica serovar Agona SL483	NC_011148.1; NC_011149.1
Salmonella enterica serovar Choleraesuis SC B67	NC_006855.1; NC_006856.1; NC_006905.1
Salmonella enterica serovar Dublin CT 02021853	NC_011204.1; NC_011205.1
Salmonella enterica serovar Enteritidis P125109	NC_011294.1
Salmonella enterica serovar Gallinarum 287 91	NC_011274.1
Salmonella enterica serovar Heidelberg SL476	NC_011081.1; NC_011082.1; NC_011083.1
Salmonella enterica serovar Newport SL254	NC_009140.1; NC_011079.1; NC_011080.1
Salmonella enterica serovar Paratyphi A AKU 12601	NC_011147.1
Salmonella enterica serovar Paratyphi A ATCC 9150	NC_006511.1
Salmonella enterica serovar Paratyphi B SPB7	NC_010102.1
Salmonella enterica serovar Paratyphi C RKS4594	NC_012124.1; NC_012125.1
Salmonella enterica serovar Schwarzengrund CVM19633	NC_011092.1; NC_011093.1; NC_011094.1
Salmonella enterica serovar Typhi CT18	NC_003198.1; NC_003384.1; NC_003385.1
Salmonella enterica serovar Typhimurium LT2	NC_003197.1; NC_003277.1
Salmonella enterica serovar Typhi Ty2	NC_004631.1
Sanguibacter keddieii DSM 10542	NC_013521.1
Sebaldella termitidis ATCC 33386	NC_013517.1; NC_013518.1; NC_013519.1
Segniliparus rotundus DSM 44985	NC_014168.1
Serratia AS12	NC_015566.1
Serratia AS9	NC_015567.1
Serratia proteamaculans 568	NC_009829.1; NC_009832.1
Shewanella amazonensis SB2B	NC_008700.1
Shewanella ANA 3	NC_008573.1; NC_008577.1
Shewanella baltica OS155	NC_009035.1; NC_009036.1; NC_009037.1; NC_009038.1; NC_009052.1
Shewanella baltica OS185	NC_009661.1; NC_009665.1
Shewanella baltica OS195	NC_009997.1; NC_009998.1; NC_009999.1; NC_010000.1
Shewanella baltica OS223	NC_011663.1; NC_011664.1; NC_011665.1; NC_011668.1
Shewanella denitrificans OS217	NC_007954.1
Shewanella frigidimarina NCIMB 400	NC_008345.1
Shewanella halifaxensis HAW EB4	NC_010334.1

Shewanella loihica PV 4	NC_009092.1
Shewanella MR 4	NC_008321.1
Shewanella MR 7	NC_008320.1; NC_008322.1
Shewanella oneidensis MR 1	NC_004347.1; NC_004349.1
Shewanella pealeana ATCC 700345	NC_009901.1
Shewanella piezotolerans WP3	NC_011566.1
Shewanella putrefaciens CN 32	NC_009438.1
Shewanella sediminis HAW EB3	NC_009831.1
Shewanella violacea DSS12	NC_014012.1
Shewanella W3 18 1	NC_008750.1
Shewanella woodyi ATCC 51908	NC_010506.1
Shigella boydii CDC 3083 94	NC_010656.1; NC_010657.1; NC_010658.1; NC_010659.1; NC_010660.1; NC_010672.1
Shigella boydii Sb227	NC_007608.1; NC_007613.1
Shigella dysenteriae Sd197	NC_007606.1; NC_007607.1; NC_009344.1
Shigella flexneri 2a 2457T	NC_004741.1
Shigella flexneri 2a 301	NC_004337.2; NC_004851.1
Shigella flexneri 5 8401	NC_008258.1
Shigella sonnei Ss046	NC_007384.1; NC_007385.1; NC_009345.1; NC_009346.1; NC_009347.1
Sideroxydans lithotrophicus ES 1	NC_013959.1
Simkania negevensis Z	NC_015710.1; NC_015713.1
Sinorhizobium medicae WSM419	NC_009620.1; NC_009621.1; NC_009622.1; NC_009636.1
Sinorhizobium meliloti 1021	NC_003037.1; NC_003047.1; NC_003078.1
Sinorhizobium meliloti AK83	NC_015590.1; NC_015591.1; NC_015592.1; NC_015596.1; NC_015597.1
Slackia heliotrinireducens DSM 20476	NC_013165.1
Sodalis glossinidius morsitans	NC_007712.1; NC_007713.1; NC_007714.1; NC_007715.1
Sorangium cellulosum So ce 56	NC_010162.1
Sphaerobacter thermophilus DSM 20745	NC_013523.1; NC_013524.1
Sphingobacterium 21	NC_015277.1
Sphingobium chlorophenolicum L 1	NC_015593.1; NC_015594.1; NC_015595.1
Sphingobium japonicum UT26S	NC_014005.1; NC_014006.1; NC_014007.1; NC_014009.1; NC_014013.1
Sphingobium SYK 6	NC_015974.1; NC_015976.1
Sphingomonas wittichii RW1	NC_009507.1; NC_009508.1; NC_009511.1
Sphingopyxis alaskensis RB2256	NC_008036.1; NC_008048.1
Spirochaeta Buddy	NC_015152.1
Spirochaeta caldaria DSM 7334	NC_015732.1
Spirochaeta coccoides DSM 17374	NC_015436.1
Spirochaeta smaragdinae DSM 11293	NC_014364.1
Spirochaeta thermophila DSM 6192	NC_014484.1
Spirosoma linguale DSM 74	NC_013730.1; NC_013731.1; NC_013732.1; NC_013733.1; NC_013734.1; NC_013735.1; NC_013736.1; NC_013737.1; NC_013738.1
Stackebrandtia nassauensis DSM 44728	NC_013947.1
Staphylococcus aureus COL	NC_002951.2; NC_006629.2
Staphylococcus aureus ED98	NC_013450.1; NC_013451.1; NC_013452.1; NC_013453.1
Staphylococcus aureus JH1	NC_009619.1; NC_009632.1
Staphylococcus aureus JH9	NC_009477.1; NC_009487.1
Staphylococcus aureus MRSA252	NC_002952.2
Staphylococcus aureus MSSA476	NC_002953.3; NC_005951.1
Staphylococcus aureus Mu3	NC_009782.1
Staphylococcus aureus Mu50	NC_002758.2; NC_002774.1
Staphylococcus aureus MW2	NC_003923.1
Staphylococcus aureus N315	NC_002745.2; NC_003140.1
Staphylococcus aureus NCTC 8325	NC_007795.1
Staphylococcus aureus Newman	NC_009641.1
Staphylococcus aureus RF122	NC_007622.1
Staphylococcus aureus USA300 FPR3757	NC_007790.1; NC_007791.1; NC_007792.1; NC_007793.1
Staphylococcus aureus USA300 TCH1516	NC_010063.1; NC_010079.1; NC_012417.1
Staphylococcus carnosus TM300	NC_012121.1

Staphylococcus epidermidis ATCC 12228	NC_004461.1; NC_005003.1; NC_005004.1; NC_005005.1; NC_005006.1; NC_005007.1; NC_005008.1
Staphylococcus epidermidis RP62A	NC_002976.3; NC_006663.1
Staphylococcus haemolyticus JCSC1435	NC_007168.1; NC_007169.1; NC_007170.1; NC_007171.1
Staphylococcus lugdunensis HKU09 01	NC_013893.1
Staphylococcus pseudintermedius HKU10 03	NC_014925.1
Staphylococcus saprophyticus ATCC 15305	NC_007350.1; NC_007351.1; NC_007352.1
Staphylothermus hellenicus DSM 12710	NC_014205.1
Staphylothermus marinus F1	NC_009033.1
Starkeya novella DSM 506	NC_014217.1
Stenotrophomonas maltophilia K279a	NC_010943.1
Stenotrophomonas maltophilia R551 3	NC_011071.1
Streptobacillus moniliformis DSM 12112	NC_013515.1; NC_013516.1
Streptococcus agalactiae 2603V R	NC_004116.1
Streptococcus agalactiae A909	NC_007432.1
Streptococcus agalactiae NEM316	NC_004368.1
Streptococcus dysgalactiae equisimilis GGS 124	NC_012891.1
Streptococcus equi 4047	NC_012471.1
Streptococcus equi zooepidemicus MGCS10565	NC_011134.1
Streptococcus equi zooepidemicus	NC_012470.1
Streptococcus gallolyticus ATCC BAA 2069	NC_015215.1; NC_015219.1
Streptococcus gallolyticus UCN34	NC_013798.1
Streptococcus gordonii Challis substr CH1	NC_009785.1
Streptococcus mitis B6	NC_013853.1
Streptococcus mutans NN2025	NC_013928.1
Streptococcus mutans UA159	NC_004350.2
Streptococcus oralis Uo5	NC_015291.1
Streptococcus parasanguinis ATCC 15912	NC_015678.1
Streptococcus parauberis KCTC 11537	NC_015558.1
Streptococcus pasteurianus ATCC 43144	NC_015600.1
Streptococcus pneumoniae 670 6B	NC_014498.1
Streptococcus pneumoniae 70585	NC_012468.1
Streptococcus pneumoniae AP200	NC_014494.1
Streptococcus pneumoniae ATCC 700669	NC_011900.1
Streptococcus pneumoniae CGSP14	NC_010582.1
Streptococcus pneumoniae D39	NC_008533.1
Streptococcus pneumoniae G54	NC_011072.1
Streptococcus pneumoniae Hungary19A 6	NC_010380.1
Streptococcus pneumoniae JJA	NC_012466.1
Streptococcus pneumoniae P1031	NC_012467.1
Streptococcus pneumoniae R6	NC_003098.1
Streptococcus pneumoniae Taiwan19F 14	NC_012469.1
Streptococcus pneumoniae TCH8431 19A	NC_014251.1
Streptococcus pneumoniae TIGR4	NC_003028.3
Streptococcus pseudopneumoniae IS7493	NC_015875.1; NC_015876.1
Streptococcus pyogenes M1 GAS	NC_002737.1
Streptococcus pyogenes Manfredo	NC_009332.1
Streptococcus pyogenes MGAS10270	NC_008022.1
Streptococcus pyogenes MGAS10394	NC_006086.1
Streptococcus pyogenes MGAS10750	NC_008024.1
Streptococcus pyogenes MGAS2096	NC_008023.1
Streptococcus pyogenes MGAS315	NC_004070.1
Streptococcus pyogenes MGAS5005	NC_007297.1
Streptococcus pyogenes MGAS6180	NC_007296.1
Streptococcus pyogenes MGAS8232	NC_003485.1
Streptococcus pyogenes MGAS9429	NC_008021.1
Streptococcus pyogenes NZ131	NC_011375.1
Streptococcus pyogenes SSI 1	NC_004606.1

Streptococcus salivarius CCHSS3	NC_015760.1
Streptococcus sanguinis SK36	NC_009009.1
Streptococcus suis 05ZYH33	NC_009442.1
Streptococcus suis 98HAH33	NC_009443.1
Streptococcus suis BM407	NC_012923.1; NC_012926.1
Streptococcus suis P1 7	NC_012925.1
Streptococcus suis SC84	NC_012924.1
Streptococcus suis ST3	NC_015433.1
Streptococcus thermophilus CNRZ1066	NC_006449.1
Streptococcus thermophilus LMD 9	NC_008500.1; NC_008501.1; NC_008532.1
Streptococcus thermophilus LMG 18311	NC_006448.1
Streptococcus uberis 0140J	NC_012004.1
Streptomyces avermitilis MA 4680	NC_003155.4; NC_004719.1
Streptomyces cattleya NRRL 8057	NC_016111.1; NC_016113.1
Streptomyces coelicolor A3 2	NC_003888.3; NC_003903.1; NC_003904.1
Streptomyces flavogriseus ATCC 33331	NC_016110.1; NC_016114.1; NC_016115.1
Streptomyces griseus NBRC 13350	NC_010572.1
Streptomyces scabiei 87 22	NC_013929.1
Streptomyces SirexAA E	NC_015953.1
Streptomyces violaceusniger Tu 4113	NC_015951.1; NC_015952.1; NC_015957.1
Streptosporangium roseum DSM 43021	NC_013595.1; NC_013596.1
Sulfobacillus acidophilus TPY	NC_015757.1
Sulfolobus acidocaldarius DSM 639	NC_007181.1
Sulfolobus islandicus L D 8 5	NC_013769.1; NC_013770.1
Sulfolobus islandicus L S 2 15	NC_012589.1
Sulfolobus islandicus M 14 25	NC_012588.1
Sulfolobus islandicus M 16 27	NC_012632.1
Sulfolobus islandicus M 16 4	NC_012726.1
Sulfolobus islandicus Y G 57 14	NC_012622.1
Sulfolobus islandicus Y N 15 51	NC_012623.1; NC_012624.1
Sulfolobus solfataricus P2	NC_002754.1
Sulfolobus tokodaii 7	NC_003106.2
Sulfuricurvum kujiense DSM 16994	NC_014754.1; NC_014755.1; NC_014756.1; NC_014762.1; NC_014763.1
Sulfurihydrogenibium azorense Az Fu1	NC_012438.1
Sulfurihydrogenibium YO3AOP1	NC_010730.1
Sulfurimonas autotrophica DSM 16294	NC_014506.1
Sulfurimonas denitrificans DSM 1251	NC_007575.1
Sulfurospirillum deleyianum DSM 6946	NC_013512.1
Sulfurovum NBC37 1	NC_009663.1
Symbiobacterium thermophilum IAM 14863	NC_006177.1
Synechococcus CC9311	NC_008319.1
Synechococcus CC9605	NC_007516.1
Synechococcus CC9902	NC_007513.1
Synechococcus elongatus PCC 6301	NC_006576.1
Synechococcus elongatus PCC 7942	NC_007595.1; NC_007604.1
Synechococcus JA 2 3B a 2 13	NC_007776.1
Synechococcus JA 3 3Ab	NC_007775.1
Synechococcus PCC 7002	NC_010474.1; NC_010475.1; NC_010476.1; NC_010477.1; NC_010478.1; NC_010479.1; NC_010480.1
Synechococcus RCC307	NC_009482.1
Synechococcus WH 7803	NC_009481.1
Synechococcus WH 8102	NC_005070.1
Synechocystis PCC 6803	NC_000911.1; NC_005229.1; NC_005230.1; NC_005231.1; NC_005232.1
Syntrophobacter fumaroxidans MPOB	NC_008554.1
Syntrophobotulus glycolicus DSM 8271	NC_015172.1
Syntrophomonas wolfei Goettingen	NC_008346.1
Syntrophothermus lipocalidus DSM 12680	NC_014220.1
Syntrophus aciditrophicus SB	NC_007759.1

Taylorella asinigenitalis MCE3	NC_016043.1
Taylorella equigenitalis MCE9	NC_014914.1
Tepidanaerobacter Re1	NC_015519.1
Teredinibacter turnerae T7901	NC_012997.1
Terriglobus saanensis SP1PR4	NC_014963.1
Tetragenococcus halophilus	NC_016052.1
Thauera MZ1T	NC_011662.2; NC_011667.1
Thermaerobacter marianensis DSM 12885	NC_014831.1
Thermanaerovibrio acidaminovorans DSM 6589	NC_013522.1
Thermincola potens JR	NC_014152.1
Thermoanaerobacter brockii finnii Ako 1	NC_014964.1
Thermoanaerobacter italicus Ab9	NC_013921.1
Thermoanaerobacterium thermosaccharolyticum DSM 571	NC_014410.1
Thermoanaerobacterium xylanolyticum LX 11	NC_015555.1
Thermoanaerobacter mathranii A3	NC_014209.1
Thermoanaerobacter pseudethanolicus ATCC 33223	NC_010321.1
Thermoanaerobacter tengcongensis MB4	NC_003869.1
Thermoanaerobacter wiegelii Rt8 B1	NC_015958.1
Thermoanaerobacter X513	NC_014538.1
Thermoanaerobacter X514	NC_010320.1
Thermobaculum terrenum ATCC BAA 798	NC_013525.1; NC_013526.1
Thermobifida fusca YX	NC_007333.1
Thermobispora bispora DSM 43833	NC_014165.1
Thermococcus 4557	NC_015865.1
Thermococcus AM4	NC_016051.1
Thermococcus barophilus MP	NC_014804.1; NC_015471.1
Thermococcus gammatolerans EJ3	NC_012804.1
Thermococcus kodakarensis KOD1	NC_006624.1
Thermococcus onnurineus NA1	NC_011529.1
Thermococcus sibiricus MM 739	NC_012883.1
Thermocrinis albus DSM 14484	NC_013894.1
Thermodesulfatator indicus DSM 15286	NC_015681.1
Thermodesulfobacterium OPB45	NC_015682.1
Thermodesulfobium narugense DSM 14796	NC_015499.1
Thermodesulfovibrio yellowstonii DSM 11347	NC_011296.1
Thermofilum pendens Hrk 5	NC_008696.1; NC_008698.1
Thermomicrobium roseum DSM 5159	NC_011959.1; NC_011961.1
Thermomonospora curvata DSM 43183	NC_013510.1
Thermoplasma acidophilum DSM 1728	NC_002578.1
Thermoplasma volcanium GSS1	NC_002689.2
Thermoproteus neutrophilus V24Sta	NC_010525.1
Thermoproteus tenax Kra 1	NC_016070.1
Thermoproteus uzoniensis 768 20	NC_015315.1
Thermosediminibacter oceani DSM 16646	NC_014377.1
Thermosipho africanus TCF52B	NC_011653.1
Thermosipho melanesiensis BI429	NC_009616.1
Thermosphaera aggregans DSM 11486	NC_014160.1
Thermosynechococcus elongatus BP 1	NC_004113.1
Thermotoga lettingae TMO	NC_009828.1
Thermotoga maritima MSB8	NC_000853.1
Thermotoga naphthophila RKU 10	NC_013642.1
Thermotoga neapolitana DSM 4359	NC_011978.1
Thermotoga petrophila RKU 1	NC_009486.1
Thermotoga RQ2	NC_010483.1
Thermotoga thermarum DSM 5069	NC_015707.1
Thermovibrio ammonificans HB 1	NC_014917.1; NC_014926.1
Thermovirga lienii DSM 17291	NC_016148.1; NC_016149.1

Thermus scotoductus SA 01	NC_014974.1; NC_014975.1
Thermus thermophilus HB27	NC_005835.1; NC_005838.1
Thermus thermophilus HB8	NC_006461.1; NC_006462.1; NC_006463.1
Thioalkalimicrobium cyclicum ALM1	NC_015581.1
Thioalkalivibrio K90mix	NC_013889.1; NC_013930.1
Thioalkalivibrio sulfidophilus HL EbGr7	NC_011901.1
Thiobacillus denitrificans ATCC 25259	NC_007404.1
Thiomicrospira crunogena XCL 2	NC_007520.2
Thiomonas intermedia K12	NC_014153.1; NC_014154.1; NC_014155.1
Tolumonas auensis DSM 9187	NC_012691.1
Treponema azotonutricium ZAS 9	NC_015577.1
Treponema brennaborense DSM 12168	NC_015500.1
Treponema denticola ATCC 35405	NC_002967.9
Treponema pallidum Nichols	NC_000919.1
Treponema pallidum SS14	NC_010741.1
Treponema paraluiscuniculi Cuniculi A	NC_015714.1
Treponema primitia ZAS 2	NC_015578.1
Treponema succinifaciens DSM 2489	NC_015385.1; NC_015386.1
Trichodesmium erythraeum IMS101	NC_008312.1
Tropheryma whipplei TW08 27	NC_004551.1
Tropheryma whipplei Twist	NC_004572.3
Truepera radiovictrix DSM 17093	NC_014221.1
Tsukamurella paurometabola DSM 20162	NC_014158.1; NC_014159.1
uncultured methanogenic archaeon RC I	NC_009464.1
uncultured Termite group 1 bacterium phylotype Rs D17	NS_000191.1; NS_000192.1; NS_000193.1; NS_000194.1
Ureaplasma parvum serovar 3 ATCC 27815	NC_010503.1
Ureaplasma parvum serovar 3 ATCC 700970	NC_002162.1
Ureaplasma urealyticum serovar 10 ATCC 33699	NC_011374.1
Variovorax paradoxus EPS	NC_014931.1
Variovorax paradoxus S110	NC_012791.1; NC_012792.1
Veillonella parvula DSM 2008	NC_013520.1
Verminephrobacter eiseniae EF01 2	NC_008771.1; NC_008786.1
Verrucosispora maris AB 18 032	NC_015409.1; NC_015434.1
Vibrio anguillarum 775	NC_015633.1; NC_015637.1
Vibrio cholerae M66 2	NC_012578.1; NC_012580.1
Vibrio cholerae MJ 1236	NC_012667.1; NC_012668.1
Vibrio cholerae O1 2010EL 1786	NC_016445.1; NC_016446.1
Vibrio cholerae O1 biovar El Tor N16961	NC_002505.1; NC_002506.1
Vibrio cholerae O395	NC_009456.1; NC_009457.1
Vibrio Ex25	NC_013456.1; NC_013457.1
Vibrio fischeri ES114	NC_006840.2; NC_006841.2; NC_006842.1
Vibrio fischeri MJ11	NC_011184.1; NC_011185.1; NC_011186.1
Vibrio harveyi ATCC BAA 1116	NC_009777.1; NC_009783.1; NC_009784.1
Vibrio parahaemolyticus RIMD 2210633	NC_004603.1; NC_004605.1
Vibrio splendidus LGP32	NC_011744.2; NC_011753.2
Vibrio vulnificus CMCP6	NC_004459.3; NC_004460.2
Vibrio vulnificus MO6 24 O	NC_014965.1; NC_014966.1
Vibrio vulnificus YJ016	NC_005128.1; NC_005139.1; NC_005140.1
Vulcanisaeta distributa DSM 14429	NC_014537.1
Vulcanisaeta moutnovskia 768 28	NC_015151.1
Waddlia chondrophila WSU 86 1044	NC_014225.1; NC_014226.1
Weeksella virosa DSM 16922	NC_015144.1
Weissella koreensis KACC 15510	NC_015756.1; NC_015759.1
Wigglesworthia glossinidia endosymbiont of Glossina brevipalpis	NC_003425.1; NC_004344.2
Wolbachia endosymbiont of Culex quinquefasciatus Pel	NC_010981.1
Wolbachia endosymbiont of Drosophila melanogaster	NC_002978.6
Wolbachia endosymbiont TRS of Brugia malayi	NC_006833.1

Wolbachia wRi	NC_012416.1
Wolinella succinogenes DSM 1740	NC_005090.1
Xanthobacter autotrophicus Py2	NC_009717.1; NC_009720.1
Xanthomonas albilineans GPE PC73	NC_013722.1
Xanthomonas axonopodis citri 306	NC_003919.1; NC_003921.3; NC_003922.1
Xanthomonas axonopodis citrumelo F1	NC_016010.1
Xanthomonas campestris 8004	NC_007086.1
Xanthomonas campestris ATCC 33913	NC_003902.1
Xanthomonas campestris B100	NC_010688.1
Xanthomonas campestris vesicatoria 85 10	NC_007504.1; NC_007505.1; NC_007506.1; NC_007507.1; NC_007508.1
Xanthomonas oryzae KACC10331	NC_006834.1
Xanthomonas oryzae MAFF 311018	NC_007705.1
Xanthomonas oryzae PXO99A	NC_010717.1
Xenorhabdus bovienii SS 2004	NC_013892.1
Xenorhabdus nematophila ATCC 19061	NC_014170.1; NC_014228.1
Xylanimonas cellulosilytica DSM 15894	NC_013530.1; NC_013531.1
Xylella fastidiosa 9a5c	NC_002488.3; NC_002489.3; NC_002490.1
Xylella fastidiosa M12	NC_010513.1
Xylella fastidiosa M23	NC_010577.1; NC_010579.1
Xylella fastidiosa Temecula1	NC_004554.1; NC_004556.1
Yersinia enterocolitica 8081	NC_008791.1; NC_008800.1
Yersinia enterocolitica palearctica 105 5R r	NC_015224.1; NC_015475.1
Yersinia pestis Angola	NC_010157.1; NC_010158.1; NC_010159.1
Yersinia pestis Antiqua	NC_008120.1; NC_008121.1; NC_008122.1; NC_008150.1
Yersinia pestis biovar Microtus 91001	NC_005810.1; NC_005813.1; NC_005814.1; NC_005815.1; NC_005816.1
Yersinia pestis CO92	NC_003131.1; NC_003132.1; NC_003134.1; NC_003143.1
Yersinia pestis KIM 10	NC_004088.1; NC_004838.1
Yersinia pestis Nepal516	NC_008118.1; NC_008119.1; NC_008149.1
Yersinia pestis Pestoides F	NC_009377.1; NC_009378.1; NC_009381.1
Yersinia pestis Z176003	NC_014017.1; NC_014022.1; NC_014027.1; NC_014029.1
Yersinia pseudotuberculosis IP 31758	NC_009704.1; NC_009705.1; NC_009708.1
Yersinia pseudotuberculosis IP 32953	NC_006153.2; NC_006154.1; NC_006155.1
Yersinia pseudotuberculosis PB1	NC_010634.1; NC_010635.1
Yersinia pseudotuberculosis YPIII	NC_010465.1
Zobellia galactanivorans	NC_015844.1
Zunongwangia profunda SM A87	NC_014041.1
Zymomonas mobilis NCIMB 11163	NC_013355.1; NC_013356.1; NC_013357.1; NC_013358.1
Zymomonas mobilis pomaceae ATCC 29192	NC_015709.1; NC_015715.1; NC_015716.1
Zymomonas mobilis ZM4	NC_006526.2
Abiotrophia defectiva ATCC 49176	NZ_GG665858.1
Acaryochloris CCMEE 5410	NZ_AFEJ01000001.1
Acetivibrio cellulolyticus CD2	NZ_AEDB02000001.1
Acetobacteraceae bacterium AT 5844	NZ_AGEZ01000001.1
Acetobacter aceti NBRC 14818	NZ_BABW01000001.1
Acetobacter pomorum DM001	NZ_AEUP01000001.1
Acetobacter tropicalis NBRC 101654	NZ_BABS01000001.1
Acetonema longum DSM 6540	NZ_AFGF01000001.1
Achromobacter piechaudii ATCC 43553	NZ_GG770409.1
Achromobacter SY8	NZ_AGUF01000001.1
Acidaminococcus D21	NZ_ACGB01000001.1
Acidiphilium PM	NZ_AFPR01000001.1
Acidithiobacillus caldus ATCC 51756	NZ_ACVD01000001.1
Acidovorax delafieldii 2AN	NZ_ACQT01000001.1
Acidovorax NO 1	NZ_AGTS01000001.1
Acidovorax radicis N35	NZ_AFBG01000001.1
Aciduliprofundum boonei T469	NZ_DS990515.1
Acinetobacter 6013113	NZ_GL891496.1

Acinetobacter 6013150	NZ_GL891614.1
Acinetobacter 6014059	NZ_GL891816.1
Acinetobacter ATCC 27244	NZ_GG665949.1
Acinetobacter baumannii AB056	NZ_ADGZ01000001.1
Acinetobacter baumannii AB058	NZ_ADHA01000001.1
Acinetobacter baumannii AB059	NZ_ADHB01000001.1
Acinetobacter baumannii AB900	NZ_ABXK01000001.1
Acinetobacter baumannii ATCC 19606	NZ_GG704572.1
Acinetobacter calcoaceticus RUH2202	NZ_GG704949.1
Acinetobacter haemolyticus ATCC 19194	NZ_GG770435.1
Acinetobacter johnsonii SH046	NZ_GG704964.1
Acinetobacter junii SH205	NZ_GG705011.1
Acinetobacter lwoffii SH145	NZ_GG705055.1
Acinetobacter NBRC 100985	NZ_BAEB01000001.1
Acinetobacter P8 3 8	NZ_AFIE01000001.1
Acinetobacter radioresistens SH164	NZ_GG705131.1
Acinetobacter radioresistens SK82	NZ_ACVR01000001.1
Acinetobacter RUH2624	NZ_GG704495.1
Acinetobacter SH024	NZ_GG753600.1
Actinobacillus minor 202	NZ_ACFT01000001.1
Actinobacillus minor NM305	NZ_ACQL01000001.1
Actinobacillus pleuropneumoniae serovar 10 D13039	NZ_ADOJ01000001.1
Actinobacillus pleuropneumoniae serovar 11 56153	NZ_ADOK01000001.1
Actinobacillus pleuropneumoniae serovar 12 1096	NZ_ADOL01000001.1
Actinobacillus pleuropneumoniae serovar 13 N273	NZ_ADOM01000001.1
Actinobacillus pleuropneumoniae serovar 1 4074	NZ_ADOD01000001.1
Actinobacillus pleuropneumoniae serovar 1 4074	NZ_AACK01000001.1
Actinobacillus pleuropneumoniae serovar 2 4226	NZ_ADXN01000001.1
Actinobacillus pleuropneumoniae serovar 2 S1536	NZ_ADOE01000001.1
Actinobacillus pleuropneumoniae serovar 4 M62	NZ_ADOF01000001.1
Actinobacillus pleuropneumoniae serovar 6 Femo	NZ_ADOG01000001.1
Actinobacillus pleuropneumoniae serovar 6 Femo	NZ_ADXO01000001.1
Actinobacillus pleuropneumoniae serovar 9 CVJ13261	NZ_ADOI01000001.1
Actinobacillus ureae ATCC 25976	NZ_GL831080.1
Actinomyces coleocanis DSM 15436	NZ_DS999539.1
Actinomyces graevenitzii C83	NZ_JH470338.1
Actinomyces odontolyticus ATCC 17982	NZ_DS264585.1
Actinomyces odontolyticus F0309	NZ_GG753639.1
Actinomyces oral taxon 170 F0386	NZ_GL882504.1
Actinomyces oral taxon 171 F0337	NZ_GL637675.1
Actinomyces oral taxon 175 F0384	NZ_AFUR01000001.1
Actinomyces oral taxon 178 F0338	NZ_GL636934.1
Actinomyces oral taxon 180 F0310	NZ_GL622194.1
Actinomyces oral taxon 448 F0400	NZ_GL985606.1
Actinomyces oral taxon 848 F0332	NZ_GG703879.1
Actinomyces oral taxon 849 F0330	NZ_JH470348.1
Actinomyces oris K20	NZ_BABV01000001.1
Actinomyces urogenitalis DSM 15434	NZ_DS999574.1
Actinomyces viscosus C505	NZ_GL877174.1
Advenella kashmirensis WT001	NZ_AFQG01000001.1
Aerococcus viridans ATCC 11563	NZ_ADNT01000001.1
Aeromicrobium marinum DSM 15272	NZ_CM001024.1
Aeromonas caviae Ae398	NZ_CACP01000001.1
Afipia 1NLS2	NZ_ADVZ01000001.1
Aggregatibacter actinomycetemcomitans D7S 1	NZ_CM000912.1
Aggregatibacter segnis ATCC 33393	NZ_GL622200.1
Agrobacterium ATCC 31749	NZ_AECL01000001.1

Ahrensia R2A130	NZ_AEEB01000001.1
Alcanivorax DG881	NZ_DS989915.1
Algoriphagus PR1	NZ_CM001023.1
Alicyclobacillus acidocaldarius LAA1	NZ_ACCS01000001.1
Alistipes HGB5	NZ_AENZ01000001.1
Alistipes indistinctus YIT 12060	NZ_JH370371.1
Alistipes putredinis DSM 17216	NZ_DS499570.1
alpha proteobacterium BAL199	NZ_ABHC01000001.1
alpha proteobacterium HIMB114	NZ_GG704918.1
Alteromonadales bacterium TW 7	NZ_AAVS01000001.1
Alteromonas macleodii ATCC 27126	NZ_ABQB01000001.1
Aminomonas paucivorans DSM 12260	NZ_CM001022.1
Anaerobaculum hydrogeniformans ATCC BAA 1850	NZ_GG705294.1
Anaerococcus hydrogenalis ACS 025 V Sch4	NZ_AEXN01000001.1
Anaerococcus hydrogenalis DSM 7454	NZ_ABXA01000001.1
Anaerococcus lactolyticus ATCC 51172	NZ_GG666044.1
Anaerococcus tetradius ATCC 35098	NZ_GG666295.1
Anaerococcus vaginalis ATCC 51170	NZ_GG700527.1
Anaerofustis stercorihominis DSM 17244	NZ_DS560015.1
Anaeroglobus geminatus F0357	NZ_JH417560.1
Anaerophaga thermohalophila DSM 12881	NZ_AEWI01000001.1
Anaerostipes 3 2 56FAA	NZ_GL629687.1
Anaerostipes caccae DSM 14662	NZ_DS499719.1
Anaerotruncus colihominis DSM 17241	NZ_DS544167.1
Anaplasma marginale Mississippi	NZ_ABOP01000001.1
Anaplasma marginale Puerto Rico	NZ_ABOQ01000001.1
Anaplasma marginale Virginia	NZ_ABOR01000001.1
Arcobacter butzleri JV22	NZ_GL622205.1
Arthrobacter globiformis NBRC 12137	NZ_BAEG01000001.1
Arthrospira maxima CS 328	NZ_ABYK01000001.1
Arthrospira platensis Paraca	NZ_ACSK01000001.1
Asticcacaulis biprosthecum C19	NZ_GL883076.1
Atopobium rimae ATCC 49626	NZ_ACFE01000001.1
Atopobium vaginae DSM 15829	NZ_ADNA01000001.1
Atopobium vaginae DSM 15829	NZ_ACGK02000001.1
Atopobium vaginae PB189 T1 4	NZ_AEDQ01000001.1
Aurantimonas manganoxydans SI85 9A1	NZ_CH672387.1
Azospirillum amazonense Y2	NZ_AFBX01000001.1
Bacillus 2 A 57 CT2	NZ_GL635750.1
Bacillus 7 6 55CFAA CT2	NZ_JH414708.1
Bacillus anthracis A0174	NZ_ABLT01000001.1
Bacillus anthracis A0193	NZ_ABKF01000001.1
Bacillus anthracis A0389	NZ_ABLB01000001.1
Bacillus anthracis A0442	NZ_ABKG01000001.1
Bacillus anthracis A0465	NZ_ABLH01000001.1
Bacillus anthracis A0488	NZ_ABJC01000001.2
Bacillus anthracis A1055	NZ_AAEO01000001.3
Bacillus anthracis A2012	NZ_AAAC02000001.1
Bacillus anthracis Australia 94	NZ_AAES01000001.1
Bacillus anthracis CNEVA 9066	NZ_AAEN01000001.1
Bacillus anthracis Kruger B	NZ_AAEQ01000001.1
Bacillus anthracis Tsiankovskii I	NZ_ABDN02000001.1
Bacillus anthracis Vollum	NZ_AAEP01000001.1
Bacillus anthracis Western North America USA6153	NZ_AAER01000001.1
Bacillus B14905	NZ_AAXV01000001.1
Bacillus BT1B CT2	NZ_GL635726.1
Bacillus cereus 03BB108	NZ_ABDM02000001.1

Bacillus cereus 172560W	NZ_CM000717.1
Bacillus cereus 95 8201	NZ_CM000727.1
Bacillus cereus AH1134	NZ_ABDA02000001.1
Bacillus cereus AH1271	NZ_CM000739.1
Bacillus cereus AH1272	NZ_CM000740.1
Bacillus cereus AH1273	NZ_CM000741.1
Bacillus cereus AH603	NZ_CM000737.1
Bacillus cereus AH621	NZ_CM000719.1
Bacillus cereus AH676	NZ_CM000738.1
Bacillus cereus ATCC 10876	NZ_CM000715.1
Bacillus cereus ATCC 4342	NZ_CM000721.1
Bacillus cereus BDRD Cer4	NZ_CM000726.1
Bacillus cereus BDRD ST196	NZ_CM000725.1
Bacillus cereus BDRD ST24	NZ_CM000723.1
Bacillus cereus BDRD ST26	NZ_CM000724.1
Bacillus cereus BGSC 6E1	NZ_CM000716.1
Bacillus cereus F65185	NZ_CM000736.1
Bacillus cereus G9241	NZ_AAEK01000001.1
Bacillus cereus H3081 97	NZ_ABDL02000001.1
Bacillus cereus m1293	NZ_CM000714.1
Bacillus cereus m1550	NZ_CM000722.1
Bacillus cereus MM3	NZ_CM000718.1
Bacillus cereus NVH0597 99	NZ_ABDK02000001.1
Bacillus cereus R309803	NZ_CM000720.1
Bacillus cereus Rock1 15	NZ_CM000729.1
Bacillus cereus Rock1 3	NZ_CM000728.1
Bacillus cereus Rock3 28	NZ_CM000730.1
Bacillus cereus Rock3 29	NZ_CM000731.1
Bacillus cereus Rock3 42	NZ_CM000732.1
Bacillus cereus Rock3 44	NZ_CM000733.1
Bacillus cereus Rock4 18	NZ_CM000735.1
Bacillus cereus Rock4 2	NZ_CM000734.1
Bacillus cereus SJ1	NZ_ADFM01000001.1
Bacillus cereus W	NZ_ABCZ02000001.1
Bacillus coahuilensis m4 4	NZ_ABFU01000001.1
Bacillus m3 13	NZ_ACPC01000001.1
Bacillus mycoides DSM 2048	NZ_CM000742.1
Bacillus mycoides Rock1 4	NZ_CM000743.1
Bacillus mycoides Rock3 17	NZ_CM000744.1
Bacillus NRRL B 14911	NZ_CH672355.1
Bacillus pseudomycoides DSM 12442	NZ_CM000745.1
Bacillus pumilus ATCC 7061	NZ_ABRX01000001.1
Bacillus SG 1	NZ_ABCF01000001.1
Bacillus smithii 7 3 47FAA	NZ_JH414738.1
Bacillus subtilis JH642	NZ_CM000489.1
Bacillus subtilis NCIB 3610	NZ_CM000488.1
Bacillus subtilis SMY	NZ_CM000490.1
Bacillus subtilis spizizenii ATCC 6633	NZ_ADGS01000001.1
Bacillus thuringiensis Bt407	NZ_CM000747.1
Bacillus thuringiensis IBL 200	NZ_CM000758.1
Bacillus thuringiensis IBL 4222	NZ_CM000759.1
Bacillus thuringiensis serovar andalousiensis BGSC 4AW1	NZ_CM000754.1
Bacillus thuringiensis serovar berliner ATCC 10792	NZ_CM000753.1
Bacillus thuringiensis serovar huazhongensis BGSC 4BD1	NZ_CM000756.1
Bacillus thuringiensis serovar israelensis ATCC 35646	NZ_AAJM01000001.1
Bacillus thuringiensis serovar kurstaki T03a001	NZ_CM000751.1
Bacillus thuringiensis serovar monterrey BGSC 4AJ1	NZ_CM000752.1

Bacillus thuringiensis serovar pakistani T13001	NZ_CM000750.1
Bacillus thuringiensis serovar pondicheriensis BGSC 4BA1	NZ_CM000755.1
Bacillus thuringiensis serovar pulsiensis BGSC 4CC1	NZ_CM000757.1
Bacillus thuringiensis serovar sotto T04001	NZ_CM000749.1
Bacillus thuringiensis serovar T01001	NZ_CM000748.1
Bacillus thuringiensis serovar tochigiensis BGSC 4Y1	NZ_CM000746.1
bacterium Ellin514	NZ_ABOX02000001.1
Bacteroides 1 1 14	NZ_GG774702.1
Bacteroides 1 1 30	NZ_GL945090.1
Bacteroides 1 1 6	NZ_GG695899.1
Bacteroides 20 3	NZ_GG774969.1
Bacteroides 2 1 16	NZ_GG705209.1
Bacteroides 2 1 22	NZ_GG705173.1
Bacteroides 2 1 33B	NZ_GG705149.1
Bacteroides 2 1 56FAA	NZ_GL945043.1
Bacteroides 2 1 7	NZ_EQ973175.1
Bacteroides 2 2 4	NZ_EQ973355.1
Bacteroides 3 1 19	NZ_GG774759.1
Bacteroides 3 1 23	NZ_GG774949.1
Bacteroides 3 1 33FAA	NZ_GG705227.1
Bacteroides 3 1 40A	NZ_GL635653.1
Bacteroides 3 2 5	NZ_GG693893.1
Bacteroides 4 1 36	NZ_GL622500.1
Bacteroides 4 3 47FAA	NZ_JH114352.1
Bacteroides 9 1 42FAA	NZ_EQ973125.1
Bacteroides caccae ATCC 43185	NZ_AAVM02000001.1
Bacteroides capillosus ATCC 29799	NZ_AAXG02000001.1
Bacteroides cellulosilyticus DSM 14838	NZ_EQ973486.1
Bacteroides clarus YIT 12056	NZ_GL882580.1
Bacteroides coprocola DSM 17136	NZ_DS981427.1
Bacteroides coprophilus DSM 18228	NZ_EQ973628.1
Bacteroides coprosuis DSM 18011	NZ_CM001167.1
Bacteroides D1	NZ_JH114330.1
Bacteroides D20	NZ_GG730105.1
Bacteroides D22	NZ_GG774795.1
Bacteroides D2	NZ_GG663451.1
Bacteroides dorei 5 1 36 D4	NZ_JH114320.1
Bacteroides dorei DSM 17855	NZ_DS995528.1
Bacteroides eggerthii 1 2 48FAA	NZ_GL622538.1
Bacteroides eggerthii DSM 20697	NZ_DS995508.1
Bacteroides finegoldii DSM 17565	NZ_GG688317.1
Bacteroides fluxus YIT 12057	NZ_GL882602.1
Bacteroides fragilis 3 1 12	NZ_EQ973213.1
Bacteroides intestinalis DSM 17393	NZ_ABJL02000001.1
Bacteroides ovatus 3 8 47FAA	NZ_GL945018.1
Bacteroides ovatus ATCC 8483	NZ_DS264553.1
Bacteroides ovatus SD CC 2a	NZ_ADMP01000001.1
Bacteroides ovatus SD CMC 3f	NZ_ADMO01000001.1
Bacteroides pectinophilus ATCC 43243	NZ_DS996920.1
Bacteroides plebeius DSM 17135	NZ_DS990117.1
Bacteroides stercoris ATCC 43183	NZ_DS499661.1
Bacteroides uniformis ATCC 8492	NZ_DS362217.1
Bacteroides vulgatus EK4 2	NZ_ADKN01000001.1
Bacteroides vulgatus PC510	NZ_ADKO01000001.1
Bacteroides xylanisolvens SD CC 1b	NZ_ADKP01000001.1
Bacteroidetes oral taxon 274 F0058	NZ_GG774889.1
Beggiatoa PS	

Beggiatoa SS	NZ_ABBY01000001.1
Bermanella marisrubri	NZ_CH724113.1
beta proteobacterium KB13	NZ_DS995299.1
Bifidobacterium 12 1 47BFAA	NZ_GL622625.1
Bifidobacterium adolescentis L2 32	NZ_DS264420.1
Bifidobacterium angulatum DSM 20098	NZ_GG663535.1
Bifidobacterium animalis lactis HN019	NZ_ABOT01000001.1
Bifidobacterium bifidum NCIMB 41171	NZ_DS990229.1
Bifidobacterium breve DSM 20213	NZ_GG729830.1
Bifidobacterium catenulatum DSM 16992	NZ_ABXY01000001.1
Bifidobacterium dentium ATCC 27678	NZ_ABIX02000001.1
Bifidobacterium dentium ATCC 27679	NZ_GL405225.1
Bifidobacterium dentium JCVIHMP022	NZ_AEHJ01000001.1
Bifidobacterium gallicum DSM 20093	NZ_ABXB03000001.1
Bifidobacterium longum DJO10A	NZ_AABM02000001.1
Bifidobacterium longum infantis ATCC 55813	NZ_GG666849.1
Bifidobacterium longum infantis CCUG 52486	NZ_DS990238.1
Bifidobacterium pseudocatenulatum DSM 20438	NZ_ABXX02000001.1
Bilophila 4 1 30	NZ_JH114231.1
Bilophila wadsworthia 3 1 6	NZ_GL622724.1
Bizionia argentinensis JUB59	NZ_AFXZ01000001.1
Blastopirellula marina DSM 3645	NZ_CH672376.1
Blautia hansenii DSM 20583	NZ_GG698588.1
Blautia hydrogenotrophica DSM 10507	NZ_GG657678.1
Borrelia afzelii ACA 1	NZ_ABCU02000001.1
Borrelia burgdorferi 118a	NZ_ABGI02000001.1
Borrelia burgdorferi 156a	NZ_ABCV02000001.1
Borrelia burgdorferi 29805	NZ_ABJX02000001.1
Borrelia burgdorferi 64b	NZ_ABKA02000001.1
Borrelia burgdorferi 72a	NZ_ABGJ02000001.1
Borrelia burgdorferi 80a	NZ_ABJU01000001.1
Borrelia burgdorferi 94a	NZ_ABGK02000001.1
Borrelia burgdorferi Bol26	NZ_ABCW02000001.1
Borrelia burgdorferi CA 11 2a	NZ_ABJY02000001.1
Borrelia burgdorferi WI91 23	NZ_ABJW02000001.1
Borrelia garinii Far04	NZ_ABPZ02000001.1
Borrelia garinii PBr	NZ_ABJV02000001.1
Borrelia spielmanii A14S	NZ_ABKB02000001.1
Borrelia SV1	NZ_ABJZ02000001.1
Borrelia valaisiana VS116	NZ_ABCY02000001.1
Bradyrhizobiaceae bacterium SG 6C	NZ_CM001195.1
Bradyrhizobium ORS 285	NZ_CAFH01000001.1
Bradyrhizobium ORS 375	NZ_CAFI01000001.1
Bradyrhizobium STM 3843	NZ_CAFK01000001.1
Bradyrhizobium	NZ_CAFJ01000001.1
Brenneria EniD312	NZ_CM001230.1
Brevibacillus laterosporus LMG 15441	NZ_AFRV01000001.1
Brevibacterium linens BL2	NZ_AAGP01000001.1
Brevibacterium mcbrellneri ATCC 49030	NZ_ADNU01000001.1
Brevundimonas BAL3	NZ_DS989898.1
Brevundimonas diminuta ATCC 11568	NZ_GL883082.1
Brucella 83 13	NZ_DS999649.1
Brucella abortus 2308 A	NZ_ACOR01000001.1
Brucella abortus bv 2 86 8 59	NZ_DS999896.1
Brucella abortus bv 3 Tulya	NZ_DS999883.1
Brucella abortus bv 4 292	NZ_DS999871.1
Brucella abortus bv 5 B3196	NZ_GG774507.1

Brucella abortus bv 6 870	NZ_DS999858.1
Brucella abortus bv 9 C68	NZ_EQ999630.1
Brucella abortus NCTC 8038	NZ_GG703762.1
Brucella BO1	NZ_ADEZ01000001.1
Brucella BO2	NZ_ADFA01000001.1
Brucella ceti B1 94	NZ_EQ999616.1
Brucella ceti Cudo	NZ_ACJD01000001.1
Brucella ceti M13 05 1	NZ_DS999686.1
Brucella ceti M490 95 1	NZ_EQ999599.1
Brucella ceti M644 93 1	NZ_DS999669.1
Brucella F5 99	NZ_GG663471.1
Brucella melitensis bv 1 16M	NZ_GG703778.1
Brucella melitensis bv 1 Rev 1	NZ_EQ999549.1
Brucella melitensis bv 2 63 9	NZ_ACEM01000001.1
Brucella melitensis bv 3 Ether	NZ_EQ999586.1
Brucella neotomae 5K33	NZ_EQ999575.1
Brucella NF 2653	NZ_ADFB01000001.1
Brucella NVSL 07 0026	NZ_GG770496.1
Brucella pinnipedialis B2 94	NZ_DS999839.1
Brucella pinnipedialis M163 99 10	NZ_DS999750.1
Brucella pinnipedialis M292 94 1	NZ_EQ999534.1
Brucella suis bv 3 686	NZ_DS999727.1
Brucella suis bv 4 40	NZ_GG703793.1
Brucella suis bv 5 513	NZ_DS999708.1
Bryantella formatexigens DSM 14469	NZ_ACCL02000001.1
Buchnera aphidicola LSR1 Acyrthosiphon pisum	NZ_ACFK01000001.1
Bulleidia extructa W1219	NZ_ADFR01000001.1
Burkholderia ambifaria IOP40 10	NZ_ABLC01000001.1
Burkholderia ambifaria MEX 5	NZ_ABLK01000001.1
Burkholderia cenocepacia PC184	NZ_CH482377.1
Burkholderia Ch1 1	NZ_ADNR01000001.1
Burkholderia dolosa AUO158	NZ_CH482380.1
Burkholderia graminis C4D1M	NZ_ABLD01000001.1
Burkholderia H160	NZ_ABYL01000001.1
Burkholderiales bacterium 1 1 47	NZ_GL383992.1
Burkholderia mallei 2002721280	NZ_CH899687.1
Burkholderia mallei ATCC 10399	NZ_CH899678.1
Burkholderia mallei FMH	NZ_DS264094.1
Burkholderia mallei GB8 horse 4	NZ_AAHO01000001.1
Burkholderia mallei JHU	NZ_DS264106.1
Burkholderia mallei PRL 20	NZ_AAZP01000001.1
Burkholderia multivorans CGD1	NZ_ACFB01000001.1
Burkholderia multivorans CGD2M	NZ_ACFD01000001.1
Burkholderia multivorans CGD2	NZ_ACFC01000001.1
Burkholderia oklahomensis C6786	NZ_ABBG01000001.1
Burkholderia oklahomensis EO147	NZ_ABBF01000001.1
Burkholderia pseudomallei 1106b	NZ_CM000774.1
Burkholderia pseudomallei 112	NZ_ABBP01000001.1
Burkholderia pseudomallei 14	NZ_ABBJ01000001.1
Burkholderia pseudomallei 1655	NZ_CH899711.1
Burkholderia pseudomallei 1710a	NZ_CM000832.1
Burkholderia pseudomallei 305	NZ_AAYX01000001.1
Burkholderia pseudomallei 406e	NZ_CH899724.1
Burkholderia pseudomallei 576	NZ_ACCE01000001.1
Burkholderia pseudomallei 7894	NZ_ABBO01000001.1
Burkholderia pseudomallei 91	NZ_ABBK01000001.1
Burkholderia pseudomallei 9	NZ_ABBL01000001.1

Burkholderia pseudomallei B7210	NZ_ABBN01000001.1
Burkholderia pseudomallei BCC215	NZ_ABBR01000001.1
Burkholderia pseudomallei DM98	NZ_ABBI01000001.1
Burkholderia pseudomallei MSHR346	NZ_ACOJ01000001.1
Burkholderia pseudomallei NCTC 13177	NZ_ABBQ01000001.1
Burkholderia pseudomallei Pakistan 9	NZ_ACKA01000001.1
Burkholderia pseudomallei Pasteur 52237	NZ_CH899740.1
Burkholderia pseudomallei S13	NZ_CH899756.1
Burkholderia thailandensis Bt4	NZ_ABBH01000001.1
Burkholderia thailandensis E264	NZ_CM000438.1
Burkholderia thailandensis MSMB43	NZ_ABBM01000001.1
Burkholderia thailandensis TXDOH	NZ_ABBD01000001.1
Burkholderia ubonensis Bu	NZ_ABBE01000001.1
Butyrivibrio crossotus DSM 2876	NZ_GG663504.1
Caldalkalibacillus thermarum TA2 A1	NZ_AFCE01000001.1
Caminibacter mediatlanticus TB 2	NZ_ABCJ01000001.1
Campylobacter 10 1 50	NZ_JH414887.1
Campylobacterales bacterium GD 1	NZ_DS995286.1
Campylobacter coli JV20	NZ_GL405235.1
Campylobacter coli RM2228	NZ_AAFL01000001.1
Campylobacter fetus venerealis Azul 94	NZ_ACLG01000001.1
Campylobacter gracilis RM3268	NZ_ACYG01000001.1
Campylobacter jejuni 1336	NZ_CM000854.1
Campylobacter jejuni 260 94	NZ_AANK01000001.1
Campylobacter jejuni 414	NZ_CM000855.1
Campylobacter jejuni 81 176	NZ_AASL01000001.1
Campylobacter jejuni 84 25	NZ_AANT02000001.1
Campylobacter jejuni BH 01 0142	NZ_ABKD01000001.1
Campylobacter jejuni CF93 6	NZ_AANJ01000001.1
Campylobacter jejuni CG8421	NZ_ABGQ01000001.1
Campylobacter jejuni CG8486	NZ_AASY01000001.2
Campylobacter jejuni HB93 13	NZ_AANQ01000001.1
Campylobacter rectus RM3267	NZ_ACFU01000001.1
Campylobacter showae RM3277	NZ_ACVQ01000001.1
Campylobacter upsaliensis JV21	NZ_GL622227.1
Campylobacter upsaliensis RM3195	NZ_AAFJ01000001.1
candidate division TM7 genomosp GTL1	NZ_AAXS01000001.1
candidate division TM7 single cell isolate TM7a	
candidate division TM7 single cell isolate TM7b	NZ_ABBW01000001.1
candidate division TM7 single cell isolate TM7c	NZ_ABBX01000001.1
Candidatus Burkholderia kirkii UZHbot1	NZ_HE603793.1
Candidatus Glomeribacter gigasporarum	NZ_CAFB01000001.1
Candidatus Haloredivivus G17	NZ_AGNT01000001.1
Candidatus Nitrosoarchaeum limnia SFB1	NZ_CM001158.1
Candidatus Odyssella thessalonicensis L13	NZ_AEWF01000001.1
Candidatus Pelagibacter HTCC7211	NZ_DS995298.1
Candidatus Pelagibacter ubique HTCC1002	NZ_CH724130.1
Candidatus Poribacteria WGA A3	NZ_ADFK01000001.1
Candidatus Regiella insecticola LSR1	NZ_CM000957.1
Candidatus Sulcia muelleri Hc Homalodisca coagulata	NZ_AANL01000001.1
Capnocytophaga gingivalis ATCC 33624	NZ_ACLQ01000001.1
Capnocytophaga ochracea F0287	NZ_GL573160.1
Capnocytophaga oral taxon 329 F0087	NZ_GL891383.1
Capnocytophaga oral taxon 338 F0234	NZ_GL872413.1
Capnocytophaga sputigena ATCC 33612	NZ_ABZV01000001.1
Carboxydibrachium pacificum DSM 12653	NZ_DS999065.1
Cardiobacterium hominis ATCC 15826	NZ_GG694025.1

Cardiobacterium valvarum F0432	NZ_JH417875.1
Carnobacterium AT7	NZ_ABHH01000001.1
Catenibacterium mitsuokai DSM 15897	NZ_ACCK01000001.1
Catonella morbi ATCC 51271	NZ_GG665854.1
Centipeda periodontii DSM 2778	NZ_GL892076.1
Chlamydia muridarum MopnTet14	NZ_ACUJ01000001.3
Chlamydia muridarum Nigg	NZ_ACOV01000001.1
Chlamydia muridarum Weiss	NZ_ACOW01000001.1
Chlamydia trachomatis 6276s	NZ_ABYE01000001.1
Chlamydia trachomatis 6276	NZ_ABYD01000001.1
Chlamydia trachomatis 70s	NZ_ABYG01000001.1
Chlamydia trachomatis 70	NZ_ABYF01000001.1
Chlamydia trachomatis D s 2923	NZ_ACFJ01000001.1
Chlamydia trachomatis L2tet1	NZ_ACUI01000001.1
Chlamydophila psittaci Cal10	NZ_AEZD01000001.1
Chlorobium ferrooxidans DSM 13031	NZ_AASE01000001.1
Chryseobacterium gleum ATCC 35910	NZ_GL379781.1
Chthoniobacter flavus Ellin428	NZ_ABVL01000001.1
Citreicella SE45	NZ_GG704597.1
Citrobacter 30 2	NZ_GG657366.1
Citrobacter freundii 4 7 47CFAA	NZ_JH414876.1
Citrobacter youngae ATCC 29220	NZ_GG730299.1
Citromicrobium bathyomarinum JL354	NZ_ADAE01000001.1
Citromicrobium JLT1363	NZ_AEUE01000001.1
Clostridiales bacterium 1 7 47FAA	NZ_DS990260.1
Clostridium 7 2 43FAA	NZ_EQ999773.1
Clostridium 7 3 54FAA	NZ_JH376512.1
Clostridium asparagiforme DSM 15981	NZ_GG657586.1
Clostridium bartlettii DSM 16795	NZ_DS499552.1
Clostridium bolteae ATCC BAA 613	NZ_DS480659.1
Clostridium botulinum Bf	NZ_ABDP01000001.1
Clostridium botulinum C Eklund	NZ_ABDQ01000001.1
Clostridium botulinum D 1873	NZ_ACSJ01000001.1
Clostridium botulinum E1 BoNT E Beluga	NZ_ACSC01000001.1
Clostridium botulinum NCTC 2916	NZ_ABDO02000001.1
Clostridium butyricum 5521	NZ_ABDT01000001.2
Clostridium butyricum E4 BoNT E BL5262	NZ_ACOM01000001.1
Clostridium carboxidivorans P7	NZ_GG770677.1
Clostridium carboxidivorans P7	NZ_ACVI01000001.1
Clostridium cellulovorans 743B	NZ_BABR01000001.1
Clostridium citroniae WAL 17108	NZ_JH376420.1
Clostridium clostridioforme 2 1 49FAA	NZ_JH376870.1
Clostridium D5	NZ_GL870809.1
Clostridium difficile ATCC 43255	NZ_CM000604.1
Clostridium difficile CIP 107932	NZ_CM000659.1
Clostridium difficile NAP07	NZ_GG770744.1
Clostridium difficile NAP08	NZ_GG770710.1
Clostridium difficile QCD 23m63	NZ_CM000660.1
Clostridium difficile QCD 32g58	NZ_CM000287.1
Clostridium difficile QCD 37x79	NZ_CM000658.1
Clostridium difficile QCD 63q42	NZ_CM000637.1
Clostridium difficile QCD 66c26	NZ_CM000441.1
Clostridium difficile QCD 76w55	NZ_CM000661.1
Clostridium difficile QCD 97b34	NZ_CM000657.1
Clostridium DL VIII	NZ_CM001240.1
Clostridium hathewayi DSM 13479	NZ_GG667607.1
Clostridium hathewayi WAL 18680	NZ_JH379027.1

Clostridium HGF2	NZ_AENW01000001.1
Clostridium hiranonis DSM 13275	NZ_DS995354.1
Clostridium hylemonae DSM 15053	NZ_GG657759.1
Clostridium L2 50	NZ_DS480311.1
Clostridium leptum DSM 753	NZ_DS480331.1
Clostridium M62 1	NZ_GG730309.1
Clostridium methylpentosum DSM 5476	NZ_EQ973338.1
Clostridium nexile DSM 1787	NZ_DS995337.1
Clostridium papyrosolvens DSM 2782	NZ_ACXX02000001.1
Clostridium perfringens B ATCC 3626	NZ_ABDV01000001.1
Clostridium perfringens C JGS1495	NZ_ABDU01000001.2
Clostridium perfringens CPE F4969	NZ_ABDX01000001.1
Clostridium perfringens D JGS1721	NZ_ABOO01000001.1
Clostridium perfringens E JGS1987	NZ_ABDW01000001.1
Clostridium perfringens NCTC 8239	NZ_ABDY01000001.1
Clostridium ramosum DSM 1402	NZ_DS499649.1
Clostridium scindens ATCC 35704	NZ_DS499678.1
Clostridium spiroforme DSM 1552	NZ_DS562843.1
Clostridium sporogenes ATCC 15579	NZ_DS981517.1
Clostridium SS2 1	NZ_DS546996.1
Clostridium symbiosum WAL 14163	NZ_GL834305.1
Clostridium symbiosum WAL 14673	NZ_GL834357.1
Clostridium thermocellum DSM 2360	NZ_ACVX01000001.1
Clostridium thermocellum JW20	NZ_ABVG02000001.1
Collinsella aerofaciens ATCC 25986	NZ_AAVN02000001.1
Collinsella intestinalis DSM 13280	NZ_GG692710.1
Collinsella stercoris DSM 13279	NZ_DS995473.1
Collinsella tanakaei YIT 12063	NZ_JH126467.1
Comamonas testosteroni KF 1	NZ_AAUJ02000001.1
Comamonas testosteroni S44	NZ_ADVQ01000001.1
Commensalibacter intestini A911	NZ_AGFR01000001.1
Congregibacter litoralis KT71	NZ_CH672401.1
Coprobacillus 29 1	NZ_GL636577.1
Coprobacillus 3 3 56FAA	NZ_JH470424.1
Coprobacillus D7	NZ_EQ999878.1
Coprococcus comes ATCC 27758	NZ_GG662005.1
Coprococcus eutactus ATCC 27759	NZ_DS483520.1
Corynebacterium accolens ATCC 49725	NZ_GG666993.1
Corynebacterium accolens ATCC 49726	NZ_GL397138.1
Corynebacterium ammoniagenes DSM 20306	NZ_GG771285.1
Corynebacterium amycolatum SK46	NZ_ABZU01000001.1
Corynebacterium aurimucosum ATCC 700975	NZ_ACLH01000001.1
Corynebacterium bovis DSM 20582	NZ_AENJ01000001.1
Corynebacterium casei	NZ_CAFW01000001.1
Corynebacterium efficiens YS 314	NZ_GG700683.1
Corynebacterium genitalium ATCC 33030	NZ_CM000961.1
Corynebacterium glucuronolyticum ATCC 51866	NZ_GG667031.1
Corynebacterium glucuronolyticum ATCC 51867	NZ_GG667127.1
Corynebacterium jeikeium ATCC 43734	NZ_GG700813.1
Corynebacterium lipophiloflavum DSM 44291	NZ_GG667191.1
Corynebacterium matruchotii ATCC 14266	NZ_ACSH02000001.1
Corynebacterium matruchotii ATCC 33806	NZ_EQ973328.1
Corynebacterium nuruki S6 4	NZ_AFIZ01000001.1
Corynebacterium pseudogenitalium ATCC 33035	NZ_GL542874.1
Corynebacterium striatum ATCC 6940	NZ_GG667519.1
Corynebacterium tuberculostearicum SK141	NZ_ACVP01000001.1
Coxiella burnetii MSU Goat Q177	NZ_AAUP02000001.2

Coxiella burnetii RSA 334	NZ_AAYJ01000001.1
Crocosphaera watsonii WH 8501	NZ_AADV02000001.1
Cyanobium PCC 7001	NZ_DS990556.1
Cyanothece ATCC 51472	NZ_AGJC01000001.1
Cyanothece CCY0110	NZ_AAXW01000001.1
Cylindrospermopsis raciborskii CS 505	NZ_ACYA01000001.1
delta proteobacterium MLMS 1	NZ_AAQF01000001.1
delta proteobacterium NaphS2	NZ_ADZZ01000001.1
Dermacoccus Ellin185	NZ_AEIQ01000001.1
Desmospora 8437	NZ_GL892032.1
Desulfitobacterium metallireducens DSM 15288	NZ_AGJB01000001.1
Desulfobacter postgatei 2ac9	NZ_AGJR01000001.1
Desulfonatronospira thiodismutans ASO3 1	NZ_ACJN02000001.1
Desulfosporosinus meridiei DSM 13257	NZ_AGJA01000001.1
Desulfosporosinus OT	NZ_AGAF01000001.1
Desulfotomaculum gibsoniae DSM 7213	NZ_AGJQ01000001.1
Desulfotomaculum nigrificans DSM 574	NZ_AEVP01000001.1
Desulfovibrio 3 1 syn3	NZ_GL384287.1
Desulfovibrio 6 1 46AFAA	NZ_JH114300.1
Desulfovibrio A2	NZ_AGFG01000001.1
Desulfovibrio fructosovorans JJ	NZ_AECZ01000001.1
Desulfovibrio FW1012B	NZ_CM001368.1
Desulfovibrio piger ATCC 29098	NZ_DS996351.1
Desulfurococcus fermentans DSM 16532	NZ_AGIW01000001.1
Desulfuromonas acetoxidans DSM 684	NZ_AAEW02000001.1
Dethiobacter alkaliphilus AHT 1	NZ_ACJM01000001.1
Dethiosulfovibrio peptidovorans DSM 11002	NZ_ABTR02000001.1
Dialister invisus DSM 15470	NZ_GG698602.1
Dialister micraerophilus DSM 19965	NZ_GL878519.1
Dialister microaerophilus UPII 345 E	NZ_AENT01000001.1
Dietzia cinnamea P4	NZ_AEKG01000001.1
Dokdonia donghaensis MED134	NZ_CH672388.1
Dorea formicigenerans 4 6 53AFAA	NZ_JH126487.1
Dorea formicigenerans ATCC 27755	NZ_AAXA02000001.1
Dorea longicatena DSM 13814	NZ_DS264384.1
Dysgonomonas gadei ATCC BAA 286	NZ_GL891979.1
Dysgonomonas mossii DSM 22836	NZ_GL892004.1
Edwardsiella tarda ATCC 23685	NZ_GG739629.1
Eggerthella 1 3 56FAA	NZ_GL622579.1
Eggerthella HGA1	NZ_AEXR01000001.1
Ehrlichia chaffeensis Sapulpa	NZ_AAIF01000001.1
Eikenella corrodens ATCC 23834	NZ_EQ973316.1
Elizabethkingia anophelis Ag1	NZ_AHHG01000001.1
Endoriftia persephone Hot96 1 Hot96 2	NZ_AASF01000001.1
endosymbiont of Riftia pachyptila vent Ph05	NZ_AFOC01000001.1
endosymbiont of Tevnia jerichonana vent Tica	NZ_AFZB01000001.1
Enhydrobacter aerosaccus SK60	NZ_ACYI01000001.1
Enterobacter cancerogenus ATCC 35316	NZ_GG704863.1
Enterobacter hormaechei ATCC 49162	NZ_GL892086.1
Enterobacteriaceae bacterium 9 2 54FAA	NZ_GL622686.1
Enterobacter mori LMG 25706	NZ_GL890773.1
Enterococcus casseliflavus ATCC 12755	NZ_GL872323.1
Enterococcus casseliflavus EC10	NZ_GG692815.1
Enterococcus casseliflavus EC20	NZ_GG670300.1
Enterococcus casseliflavus EC30	NZ_GG670385.1
Enterococcus faecalis ARO1 DG	NZ_GG692898.1
Enterococcus faecalis ATCC 29200	NZ_GG668755.1

Enterococcus faecalis ATCC 4200	NZ_GG670371.1
Enterococcus faecalis CH188	NZ_GG688650.1
Enterococcus faecalis D6	NZ_GG688626.1
Enterococcus faecalis DAPTO 512	NZ_GL455784.1
Enterococcus faecalis DAPTO 516	NZ_GL455705.1
Enterococcus faecalis DS5	NZ_GG692855.1
Enterococcus faecalis E1Sol	NZ_GG692672.1
Enterococcus faecalis Fly1	NZ_GG692660.1
Enterococcus faecalis HH22	NZ_GG668819.1
Enterococcus faecalis HIP11704	NZ_GG692622.1
Enterococcus faecalis JH1	NZ_GG692686.1
Enterococcus faecalis Merz96	NZ_GG692911.1
Enterococcus faecalis PC1 1	NZ_ADMM01000001.1
Enterococcus faecalis R712	NZ_GG739821.1
Enterococcus faecalis S613	NZ_GG739716.1
Enterococcus faecalis T11	NZ_GG688637.1
Enterococcus faecalis T1	NZ_GG670345.1
Enterococcus faecalis T2	NZ_GG692833.1
Enterococcus faecalis T3	NZ_GG670361.1
Enterococcus faecalis T8	NZ_GG698872.1
Enterococcus faecalis TUSoD Ef11	NZ_ACOX02000001.1
Enterococcus faecalis TX0102	NZ_GL455093.1
Enterococcus faecalis TX0104	NZ_GG668922.1
Enterococcus faecalis TX0109	NZ_GL455412.1
Enterococcus faecalis TX0411	NZ_GL456398.1
Enterococcus faecalis TX0470	NZ_GL456509.1
Enterococcus faecalis TX0635	NZ_GL455326.1
Enterococcus faecalis TX0855	NZ_GL455640.1
Enterococcus faecalis TX0860	NZ_GL455490.1
Enterococcus faecalis TX1322	NZ_GG669017.1
Enterococcus faecalis TX2134	NZ_GL455562.1
Enterococcus faecalis TX4248	NZ_GL454407.1
Enterococcus faecalis X98	NZ_GG688423.1
Enterococcus faecium 1 141 733	NZ_GG688461.1
Enterococcus faecium 1 230 933	NZ_GG692537.1
Enterococcus faecium 1 231 408	NZ_GG688547.1
Enterococcus faecium 1 231 410	NZ_GG692468.1
Enterococcus faecium 1 231 501	NZ_GG688436.1
Enterococcus faecium 1 231 502	NZ_GG688486.1
Enterococcus faecium C68	NZ_GG703698.1
Enterococcus faecium Com12	NZ_GG670306.1
Enterococcus faecium Com15	NZ_GG670325.1
Enterococcus faecium D344SRF	NZ_ACZZ01000001.1
Enterococcus faecium DO	NZ_AAAK03000001.1
Enterococcus faecium DO	NZ_ACIY01000001.1
Enterococcus faecium E1039	NZ_ACOS01000001.1
Enterococcus faecium E1071	NZ_ABQI01000001.1
Enterococcus faecium E1162	NZ_ABQJ01000001.1
Enterococcus faecium E1636	NZ_ABRY01000001.1
Enterococcus faecium E1679	NZ_ABSC01000001.1
Enterococcus faecium E980	NZ_ABQA01000001.1
Enterococcus faecium TC 6	NZ_GG703573.1
Enterococcus faecium TX0082	NZ_GL455858.1
Enterococcus faecium TX0133a01	NZ_GL476015.1
Enterococcus faecium TX0133a04	NZ_GL454903.1
Enterococcus faecium TX0133A	NZ_GL456551.1
Enterococcus faecium TX0133B	NZ_GL456794.1

Enterococcus faecium TX0133C	NZ_GL455133.1
Enterococcus faecium TX1330	NZ_GG669057.1
Enterococcus faecium U0317	NZ_ABSW01000001.1
Enterococcus gallinarum EG2	NZ_GG670286.1
Enterococcus italicus DSM 15952	NZ_GL622241.1
Enterococcus saccharolyticus 30 1	NZ_JH376939.1
Epulopiscium N t morphotype B	NZ_ABEQ01000001.3
Eremococcus coleocola ACS 139 V Col8	NZ_AENN01000001.1
Erysipelothrix rhusiopathiae ATCC 19414	NZ_ACLK02000001.1
Erysipelotrichaceae bacterium 2 2 44A	NZ_JH126431.1
Erysipelotrichaceae bacterium 3 1 53	NZ_GL520132.1
Erysipelotrichaceae bacterium 5 2 54FAA	NZ_GG749077.1
Erythrobacter NAP1	NZ_CH672390.1
Erythrobacter SD 21	NZ_ABCG01000001.1
Escherichia 1 1 43	NZ_GG665811.1
Escherichia 3 2 53FAA	NZ_DS999459.1
Escherichia 4 1 40B	NZ_GG657415.1
Escherichia albertii TW07627	NZ_CH991859.1
Escherichia coli 101 1	NZ_AAMK02000001.1
Escherichia coli 1827 70	NZ_ADUK01000001.1
Escherichia coli 2362 75	NZ_ADUL01000001.1
Escherichia coli 53638	NZ_AAKB02000001.1
Escherichia coli 83972	NZ_GG669095.1
Escherichia coli B088	NZ_GG749125.1
Escherichia coli B171	NZ_AAJX02000001.1
Escherichia coli B185	NZ_GG749162.1
Escherichia coli B354	NZ_GG749326.1
Escherichia coli B7A	NZ_AAJT02000001.1
Escherichia coli E110019	NZ_AAJW02000001.1
Escherichia coli E22	NZ_AAJV02000001.1
Escherichia coli F11	NZ_AAJU02000001.1
Escherichia coli FVEC1302	NZ_GG774899.1
Escherichia coli FVEC1412	NZ_GG749206.1
Escherichia coli H299	NZ_GL884490.1
Escherichia coli H591	NZ_GL884408.1
Escherichia coli H736	NZ_GL883767.1
Escherichia coli M605	NZ_GL883892.1
Escherichia coli M718	NZ_GL884101.1
Escherichia coli MS 107 1	NZ_ADWV01000001.1
Escherichia coli MS 115 1	NZ_GG771708.1
Escherichia coli MS 116 1	NZ_GG773342.1
Escherichia coli MS 119 7	NZ_ADWU01000001.1
Escherichia coli MS 124 1	NZ_ADWT01000001.1
Escherichia coli MS 145 7	NZ_ADWS01000001.1
Escherichia coli MS 146 1	NZ_GG772040.1
Escherichia coli MS 175 1	NZ_GG773588.1
Escherichia coli MS 182 1	NZ_GG771869.1
Escherichia coli MS 185 1	NZ_GG774191.1
Escherichia coli MS 187 1	NZ_GG772477.1
Escherichia coli MS 196 1	NZ_GG773880.1
Escherichia coli MS 198 1	NZ_GG771311.1
Escherichia coli MS 200 1	NZ_GG773727.1
Escherichia coli MS 21 1	NZ_GG772584.1
Escherichia coli MS 45 1	NZ_GG772175.1
Escherichia coli MS 69 1	NZ_GG772327.1
Escherichia coli MS 78 1	NZ_GG773215.1
Escherichia coli MS 84 1	NZ_GG771526.1

Escherichia coli NC101	NZ_AEFA01000001.1
Escherichia coli O157 H7 EC4024	NZ_DS571061.1
Escherichia coli O157 H7 EC4042	NZ_ABHM02000001.1
Escherichia coli O157 H7 EC4045	NZ_ABHL02000001.1
Escherichia coli O157 H7 EC4076	NZ_ABHQ01000001.1
Escherichia coli O157 H7 EC4113	NZ_ABHP01000001.1
Escherichia coli O157 H7 EC4196	NZ_ABHO01000001.1
Escherichia coli O157 H7 EC4206	NZ_ABHK02000001.1
Escherichia coli O157 H7 EC4401	NZ_ABHR01000001.1
Escherichia coli O157 H7 EC4486	NZ_ABHS01000001.1
Escherichia coli O157 H7 EC4501	NZ_ABHT01000001.1
Escherichia coli O157 H7 EC508	NZ_ABHW01000001.1
Escherichia coli O157 H7 EC869	NZ_ABHU01000001.1
Escherichia coli O157 H7 FRIK2000	NZ_ACXO01000001.1
Escherichia coli O157 H7 FRIK966	NZ_ACXN01000001.1
Escherichia coli O157 H7 TW14588	NZ_CM000662.1
Escherichia coli OP50	NZ_ADBT01000001.1
Escherichia coli TA143	NZ_GL884206.1
Escherichia coli TA206	NZ_GL884168.1
Escherichia coli TA271	NZ_GL884249.1
Escherichia coli TA280	NZ_GL884332.1
Escherichia coli W	NZ_AEDF01000001.1
Escherichia TW09308	NZ_AEME01000001.1
Eubacteriaceae bacterium ACC19a	NZ_JH414546.1
Eubacteriaceae bacterium CM2	NZ_JH414513.1
Eubacteriaceae bacterium CM5	NZ_JH414596.1
Eubacterium biforme DSM 3989	NZ_DS996836.1
Eubacterium cellulosolvens 6	NZ_AEOA01000001.1
Eubacterium dolichum DSM 3991	NZ_DS483460.1
Eubacterium hallii DSM 3353	NZ_ACEP01000001.1
Eubacterium saburreum DSM 3986	NZ_GL622296.1
Eubacterium saphenum ATCC 49989	NZ_GG688422.1
Eubacterium siraeum DSM 15702	NZ_DS499508.1
Eubacterium ventriosum ATCC 27560	NZ_DS264262.1
Eubacterium yurii margaretiae ATCC 43715	NZ_GL405246.1
Faecalibacterium cf prausnitzii KLE1255	NZ_GL538213.1
Faecalibacterium prausnitzii A2 165	NZ_GG697149.2
Faecalibacterium prausnitzii M21 2	NZ_DS483479.1
Ferroplasma acidarmanus fer1	NZ_CM000428.1
Fervidobacterium pennivorans DSM 9078	NZ_AGJO01000001.1
Finegoldia magna ACS 171 V Col3	NZ_AECM01000001.1
Finegoldia magna ATCC 53516	NZ_CM000955.1
Finegoldia magna BVS033A4	NZ_AEDP01000001.1
Fischerella JSC 11	NZ_AGIZ01000001.1
Flavobacteria bacterium BAL38	NZ_AAXX01000001.1
Flavobacteria bacterium BBFL7	NZ_CH672374.1
Flavobacteria bacterium MS024 2A	NZ_ABVV01000001.1
Flavobacteria bacterium MS024 3C	NZ_ABVW01000001.1
Flavobacteriaceae bacterium HQM9	NZ_AFPB01000001.1
Flavobacteriales bacterium ALC 1	NZ_ABHI01000001.1
Flavonifractor plautii ATCC 29863	NZ_JH417617.1
Francisella novicida FTE	NZ_DS989818.1
Francisella novicida FTG	NZ_DS995363.1
Francisella novicida GA99 3548	NZ_DS264587.1
Francisella novicida GA99 3549	NZ_DS264124.1
Francisella philomiragia ATCC 25015	NZ_DS999308.1
Francisella tularensis FSC033	NZ_DS264116.1

Francisella tularensis holarctica 257	NZ_DS229038.1
Francisella tularensis holarctica FSC022	NZ_DS264133.1
Francisella tularensis holarctica FSC200	NZ_AASP01000001.1
Francisella tularensis holarctica URFT1	NZ_ABAZ01000001.1
Francisella tularensis MA00 2987	NZ_DS990210.1
Frankia CN3	NZ_AGJN01000001.1
Frankia EUN1f	NZ_ADGX01000001.1
Fructobacillus fructosus KCTC 3544	NZ_AEOP01000001.1
Fulvimarina pelagi HTCC2506	NZ_DS022272.1
Fusobacterium 11 3 2	NZ_GL945391.1
Fusobacterium 1 1 41FAA	NZ_GG770374.1
Fusobacterium 21 1A	NZ_GL945387.1
Fusobacterium 2 1 31	NZ_GL988006.1
Fusobacterium 3 1 27	NZ_GG770328.1
Fusobacterium 3 1 33	NZ_GG704455.1
Fusobacterium 3 1 36A2	NZ_GG698790.1
Fusobacterium 3 1 5R	NZ_GG657971.1
Fusobacterium 4 1 13	NZ_GG657999.1
Fusobacterium 7 1	NZ_GG658011.1
Fusobacterium D11	NZ_EQ999802.1
Fusobacterium D12	NZ_GL988012.1
Fusobacterium gonidiaformans ATCC 25563	NZ_GG658179.1
Fusobacterium mortiferum ATCC 9817	NZ_GL987987.1
Fusobacterium nucleatum ATCC 23726	NZ_ADVK01000001.1
Fusobacterium nucleatum polymorphum ATCC 10953	NZ_CM000440.1
Fusobacterium nucleatum vincentii ATCC 49256	NZ_AABF02000001.1
Fusobacterium oral taxon 370 F0437	NZ_JH378894.1
Fusobacterium periodonticum ATCC 33693	NZ_GG665876.1
Fusobacterium ulcerans ATCC 49185	NZ_GG658104.1
Fusobacterium varium ATCC 27725	NZ_GL987995.1
gamma proteobacterium HTCC2207	NZ_CH672396.1
gamma proteobacterium HTCC5015	NZ_DS990598.1
gamma proteobacterium IMCC1989	NZ_AEVK01000001.1
gamma proteobacterium IMCC2047	NZ_AEGL01000001.1
gamma proteobacterium IMCC3088	NZ_AEIG01000001.1
gamma proteobacterium NOR51 B	NZ_DS999411.1
gamma proteobacterium NOR5 3	NZ_DS999405.1
gamma proteobacterium SCGC AAA001 B15	NZ_AFHZ01000001.1
Gardnerella vaginalis 5 1	NZ_ADAN01000001.1
Gardnerella vaginalis AMD	NZ_ADAM01000001.1
Gardnerella vaginalis ATCC 14018	NZ_ADNB01000001.1
Gemella haemolysans ATCC 10379	NZ_ACDZ02000001.1
Gemella haemolysans M341	NZ_GL883582.1
Gemella moribillum M424	NZ_GL622607.1
Gemella sanguinis M325	NZ_GL883552.1
Gemmata obscuriglobus UQM 2246	NZ_ABGO01000001.1
Geobacillus G11MC16	NZ_ABVH01000001.1
get-table.sh	
Glaciecola HTCC2999	NZ_DS989810.1
Gluconacetobacter europaeus LMG 18494	NZ_CADR01000001.1
Gluconacetobacter hansenii ATCC 23769	NZ_CM000920.1
Gluconacetobacter oboediens 174Bp2	NZ_CADT01000001.1
Gluconacetobacter SXCC 1	NZ_AFCH01000001.1
Gluconobacter morbifer G707	NZ_AGQV01000001.1
Gordonia alkanivorans NBRC 16433	NZ_BACI01000001.1
Gordonia amarae NBRC 15530	NZ_BAED01000001.1
Gordonia araii NBRC 100433	NZ_BAEE01000001.1

Gordonia effusa NBRC 100432	NZ_BAEH01000001.1
Gordonia neofelifaecis NRRL B 59395	NZ_AEUD01000001.1
Gordonia polyisoprenivorans NBRC 16320	NZ_BAEI01000001.1
Granulicatella adiacens ATCC 49175	NZ_GG694015.1
Granulicatella elegans ATCC 700633	NZ_GG703805.1
Grimontia hollisae CIP 101886	NZ_ADAQ01000001.1
Haemophilus aegyptius ATCC 11116	NZ_GL878526.1
Haemophilus haemolyticus M21621	NZ_AFQQ01000001.1
Haemophilus influenzae 22 1 21	NZ_AAZD01000001.1
Haemophilus influenzae 3655	NZ_AAZF01000001.1
Haemophilus influenzae 6P18H1	NZ_ABWW01000001.1
Haemophilus influenzae 7P49H1	NZ_ABWV01000001.1
Haemophilus influenzae NT127	NZ_ACSL01000001.1
Haemophilus influenzae PittAA	NZ_AAZG01000001.1
Haemophilus influenzae PittHH	NZ_AAZH01000001.1
Haemophilus influenzae PittII	NZ_AAZI01000001.1
Haemophilus influenzae R3021	NZ_AAZJ01000001.1
Haemophilus influenzae R3021	NZ_AAZE01000001.1
Haemophilus influenzae RdAW	NZ_ACSM01000001.1
Haemophilus parainfluenzae ATCC 33392	NZ_GL872339.1
Haemophilus parainfluenzae CCUG 13788	NZ_AFNK01000001.1
Haemophilus parasuis 29755	NZ_ABKM01000001.1
Haemophilus pittmaniae HK 85	NZ_AFUV01000001.1
Hafnia alvei ATCC 51873	NZ_JH417481.1
Haladaptatus paucihalophilus DX253	NZ_AEMG01000001.1
Halobacterium DL1	NZ_AGIR01000001.1
Halomonas boliviensis LC1	NZ_JH393257.1
Halomonas GFAJ 1	NZ_AHBC01000001.1
Halomonas HAL1	NZ_AGIB01000001.1
Halomonas TD01	NZ_GL949754.1
Haloplasma contractile SSD 17B	NZ_AFNU01000001.1
Halorhabdus tiamatea SARL4B	NZ_AFNT01000001.1
Helicobacter bilis ATCC 43879	NZ_GG661874.1
Helicobacter canadensis MIT 98 5491	NZ_DS990368.1
Helicobacter canadensis MIT 98 5491	NZ_CM000776.2
Helicobacter cinaedi CCUG 18818	NZ_DS990391.1
Helicobacter pullorum MIT 98 5489	NZ_DS990441.1
Helicobacter pylori 98 10	NZ_ABSX01000001.1
Helicobacter pylori B128	NZ_ABSY01000001.1
Helicobacter pylori HPKX 438 AG0C1	NZ_ABJO01000001.1
Helicobacter pylori HPKX 438 CA4C1	NZ_ABJP01000001.1
Helicobacter suis HS1	NZ_ADGY01000001.1
Helicobacter suis HS5	NZ_ADHO01000001.1
Helicobacter winghamensis ATCC BAA 430	NZ_GG661973.1
Hoeflea phototrophica DFL 43	NZ_DS544889.1
Holdemania filiformis DSM 12042	NZ_GG657551.1
Hydrogenivirga 128 5 R1 1	NZ_ABHJ01000001.1
Hylemonella gracilis ATCC 19624	NZ_AEGR01000001.1
Idiomarina A28L	NZ_AFPO01000001.1
Idiomarina baltica OS145	NZ_CH672403.1
Janibacter HTCC2649	NZ_CH672413.1
Johnsonella ignava ATCC 51276	NZ_JH378829.1
Jonquetella anthropi E3 33 E1	NZ_GG697147.2
Kingella denitrificans ATCC 33394	NZ_GL870929.1
Kingella kingae ATCC 23330	NZ_GL891959.1
Kingella oralis ATCC 51147	NZ_GG665871.1
Klebsiella 1 1 55	NZ_GG745508.1

Klebsiella 4 1 44FAA	NZ_JH414798.1
Klebsiella MS 92 3	NZ_GL882699.1
Klebsiella pneumoniae rhinoscleromatis ATCC 13884	NZ_GG703522.1
Kordia algicida OT 1	NZ_DS544873.1
Ktedonobacter racemifer DSM 44963	NZ_ADVG01000001.1
Labrenzia aggregata IAM 12614	NZ_AAUW01000001.1
Labrenzia alexandrii DFL 11	NZ_EQ973119.1
Lachnospiraceae bacterium 1 1 57FAA	NZ_GL945294.1
Lachnospiraceae bacterium 1 4 56FAA	NZ_GL945163.1
Lachnospiraceae bacterium 2 1 46FAA	NZ_GL890503.1
Lachnospiraceae bacterium 2 1 58FAA	NZ_GL945333.1
Lachnospiraceae bacterium 3 1 46FAA	NZ_GL890520.1
Lachnospiraceae bacterium 3 1 57FAA CT1	NZ_GL945195.1
Lachnospiraceae bacterium 4 1 37FAA	NZ_GL872349.1
Lachnospiraceae bacterium 5 1 57FAA	NZ_GL945243.1
Lachnospiraceae bacterium 5 1 63FAA	NZ_GL622401.1
Lachnospiraceae bacterium 6 1 63FAA	NZ_GL890548.1
Lachnospiraceae bacterium 8 1 57FAA	NZ_GL622449.1
Lachnospiraceae bacterium 9 1 43BFAA	NZ_GL890571.1
Lachnospiraceae oral taxon 107 F0167	NZ_GL890583.1
Lactobacillus 7 1 47FAA	NZ_JH164952.1
Lactobacillus acidipiscis KCTC 13900	NZ_BACS01000001.1
Lactobacillus acidophilus ATCC 4796	NZ_GG669566.1
Lactobacillus amylolyticus DSM 11664	NZ_ADNY01000001.1
Lactobacillus animalis KCTC 3501	NZ_GL573153.1
Lactobacillus antri DSM 16041	NZ_GG700732.1
Lactobacillus brevis gravesensis ATCC 27305	NZ_GG669604.1
Lactobacillus buchneri ATCC 11577	NZ_GG669710.1
Lactobacillus coleohominis 101 4 CHN	NZ_GG698802.1
Lactobacillus coryniformis KCTC 3167	NZ_GL544587.1
Lactobacillus coryniformis torquens KCTC 3535	NZ_AEOS01000001.1
Lactobacillus crispatus 125 2 CHN	NZ_GG698760.1
Lactobacillus crispatus 214 1	NZ_ADGR01000001.1
Lactobacillus crispatus CTV 05	NZ_GL531736.1
Lactobacillus crispatus JV V01	NZ_GG669814.1
Lactobacillus crispatus MV 1A US	NZ_GG698827.1
Lactobacillus crispatus MV 3A US	NZ_GG704606.1
Lactobacillus curvatus CRL 705	NZ_AGBU01000001.1
Lactobacillus delbrueckii bulgaricus PB2003 044 T3 4	NZ_AEAT01000001.1
Lactobacillus farciminis KCTC 3681	NZ_GL575016.1
Lactobacillus fermentum 28 3 CHN	NZ_GG704699.1
Lactobacillus fermentum ATCC 14931	NZ_GG669900.1
Lactobacillus fructivorans KCTC 3543	NZ_GL622178.1
Lactobacillus gasseri 202 4	NZ_ACOZ01000001.1
Lactobacillus gasseri 224 1	NZ_ADFT01000001.1
Lactobacillus gasseri JV V03	NZ_GL379580.1
Lactobacillus gasseri MV 22	NZ_GL531761.1
Lactobacillus helveticus DSM 20075	NZ_GG700752.1
Lactobacillus hilgardii ATCC 8290	NZ_GG669992.1
Lactobacillus iners AB 1	NZ_ADHG01000001.1
Lactobacillus iners ATCC 55195	NZ_GL622333.1
Lactobacillus iners DSM 13335	NZ_GG700801.1
Lactobacillus iners LactinV 01V1 a	NZ_AEHQ01000001.1
Lactobacillus iners LactinV 03V1 b	NZ_AEHP01000001.1
Lactobacillus iners LactinV 09V1 c	NZ_AEHO01000001.1
Lactobacillus iners LactinV 11V1 d	NZ_AEHN01000001.1
Lactobacillus iners LEAF 2052A d	NZ_AEKI01000001.1

Lactobacillus iners LEAF 2053A b	NZ_AEKH01000001.1
Lactobacillus iners LEAF 2062A h1	NZ_AEKJ01000001.1
Lactobacillus iners LEAF 3008A a	NZ_AEKK01000001.1
Lactobacillus iners SPIN 1401G	NZ_AEXP01000001.1
Lactobacillus iners SPIN 2503V10 D	NZ_AEHR01000001.1
Lactobacillus iners UPII 143 D	NZ_AEXJ01000001.1
Lactobacillus iners UPII 60 B	NZ_AEXK01000001.1
Lactobacillus jensenii 115 3 CHN	NZ_GG704741.1
Lactobacillus jensenii 1153	NZ_GL545251.1
Lactobacillus jensenii 208 1	NZ_ADEX01000001.1
Lactobacillus jensenii 269 3	NZ_ACOY01000001.1
Lactobacillus jensenii 27 2 CHN	NZ_GG698814.1
Lactobacillus jensenii JV V16	NZ_CM000953.1
Lactobacillus jensenii SJ 7A US	NZ_GG704682.1
Lactobacillus johnsonii ATCC 33200	NZ_GG670120.1
Lactobacillus malefermentans KCTC 3548	NZ_BACN01000001.1
Lactobacillus mali KCTC 3596	NZ_BACP01000001.1
Lactobacillus oris PB013 T2 3	NZ_AEKL01000001.1
Lactobacillus paracasei 8700 2	NZ_DS990485.1
Lactobacillus paracasei ATCC 25302	NZ_GG670152.1
Lactobacillus parafarraginis F0439	NZ_JH414900.1
Lactobacillus plantarum ATCC 14917	NZ_GL379761.1
Lactobacillus reuteri 100 23	NZ_AAPZ02000001.1
Lactobacillus reuteri CF48 3A	NZ_GG693664.1
Lactobacillus reuteri MM2 3	NZ_GG693756.1
Lactobacillus reuteri MM4 1A	NZ_ACGX02000001.1
Lactobacillus rhamnosus HN001	NZ_ABWJ01000001.1
Lactobacillus rhamnosus LMS2 1	NZ_GG692960.1
Lactobacillus ruminis ATCC 25644	NZ_GL833109.1
Lactobacillus ruminis SPM0211	NZ_AFOJ01000001.1
Lactobacillus salivarius ACS 116 V Col5a	NZ_AEBA01000001.1
Lactobacillus salivarius ATCC 11741	NZ_GG693221.1
Lactobacillus suebicus KCTC 3549	NZ_BACO01000001.1
Lactobacillus ultunensis DSM 16047	NZ_GG693253.1
Lactobacillus vaginalis ATCC 49540	NZ_GG693412.1
Lactobacillus versmoldensis KCTC 3814	NZ_BACR01000001.1
Lactobacillus zeae KCTC 3804	NZ_BACQ01000001.1
Lautropia mirabilis ATCC 51599	NZ_GL636062.1
Leeuwenhoekiella blandensis MED217	NZ_CH672395.1
Legionella drancourtii LLAP12	JH413793.1
Legionella longbeachae D 4968	NZ_ACZG01000001.1
Lentisphaera araneosa HTCC2155	NZ_ABCK01000001.1
Leptolyngbya valderiana BDU 20041	NZ_AAZV01000001.1
Leptospira licerasiae serovar Varillal MMD0835	NZ_AFLO01000001.1
Leptospira noguchii 2006001870	NZ_AFLY01000001.1
Leptospira santarosai 2000030832	NZ_AFJN01000001.1
Leptospira weilii 2006001855	NZ_AFJM01000001.1
Leptotrichia goodfellowii F0264	NZ_ADAD01000001.1
Leptotrichia hofstadii F0254	NZ_GG700632.1
Leuconostoc argentinum KCTC 3773	NZ_AEGQ01000001.1
Leuconostoc fallax KCTC 3537	NZ_AEIZ01000001.1
Leuconostoc gelidum KCTC 3527	NZ_AEMI01000001.1
Leuconostoc inhae KCTC 3774	NZ_AEMJ01000001.1
Leuconostoc lactis KCTC 3528	NZ_AEOR01000001.1
Leuconostoc mesenteroides cremoris ATCC 19254	NZ_GG693383.1
Leuconostoc pseudomesenteroides KCTC 3652	NZ_AEOQ01000001.1
Limnobacter MED105	NZ_ABCT01000001.1

Listeria grayi DSM 20601	NZ_GL538352.1
Listeria ivanovii FSL F6 596	NZ_CM001050.1
Listeria marthii FSL S4 120	NZ_CM001047.1
Listeria monocytogenes F6900	NZ_AARU02000001.1
Listeria monocytogenes FSL F2 515	NZ_AARI02000001.1
Listeria monocytogenes FSL J1 175	NZ_AARK02000001.1
Listeria monocytogenes FSL J1 194	NZ_AARJ02000001.1
Listeria monocytogenes FSL J1 208	NZ_AARL02000001.1
Listeria monocytogenes FSL J2 003	NZ_AARM02000001.1
Listeria monocytogenes FSL J2 064	NZ_AARO02000001.1
Listeria monocytogenes FSL J2 071	NZ_AARN04000001.1
Listeria monocytogenes FSL N1 017	NZ_AARP04000001.1
Listeria monocytogenes FSL N3 165	NZ_AARQ02000001.1
Listeria monocytogenes FSL R2 503	NZ_AARR02000001.1
Listeria monocytogenes HPB2262	NZ_AATL02000001.1
Listeria monocytogenes J2818	NZ_AARX02000001.1
Listeria monocytogenes LO28	NZ_AARY02000001.1
Listeria monocytogenes serotype 1 2a F6854	NZ_AADQ01000001.1
Listeria monocytogenes serotype 4b H7858	NZ_AADR01000001.1
Loktanella vestfoldensis SKA53	NZ_CH672414.1
Lutiella nitroferrum 2002	NZ_ACIS01000001.1
Lyngbya majuscula 3L	NZ_GL890815.1
Lyngbya PCC 8106	NZ_AAVU01000001.1
Lysinibacillus fusiformis ZC1	NZ_ADJR01000001.1
Magnetospirillum magnetotacticum MS 1	
Mannheimia haemolytica PHL213	NZ_DS264608.1
Mannheimia haemolytica serotype A2 BOVINE	NZ_ACZY01000001.1
Mannheimia haemolytica serotype A2 OVINE	NZ_ACZX01000001.1
Marichromatium purpuratum 984	NZ_AFWU01000001.1
marine actinobacterium PHSC20C1	NZ_CH672415.1
marine gamma proteobacterium HTCC2080	NZ_AAVV01000001.1
marine gamma proteobacterium HTCC2143	NZ_AAVT01000001.1
marine gamma proteobacterium HTCC2148	NZ_DS999222.1
Marinobacter algicola DG893	NZ_ABCP01000001.1
Marinobacter ELB17	NZ_AAXY01000001.1
Marinobacter MnI7 9	NZ_AGTR01000001.1
Marinomonas MED121	NZ_CH672429.1
Mariprofundus ferrooxydans PV 1	NZ_DS022294.1
Maritimibacter alkaliphilus HTCC2654	NZ_CH902578.1
Megasphaera genomosp type 1 28L	NZ_ADGP01000001.1
Megasphaera micronuciformis F0359	NZ_GL538175.1
Megasphaera UPII 135 E	NZ_AFUG01000001.1
Megasphaera UPII 199 6	NZ_AFIJ01000001.1
Mesorhizobium alhagi CCNWXJ12 2	NZ_AHAM01000001.1
Mesorhizobium amorphae CCNWGS0123	NZ_AGSN01000001.1
Mesorhizobium australicum WSM2073	NZ_AGIX01000001.1
Methanobrevibacter smithii DSM 2374	NZ_GG704759.1
Methanobrevibacter smithii DSM 2375	NZ_DS996911.1
Methanolinea tarda NOBI 1	NZ_AGIY01000001.1
Methylobacter tundripaludum SV96	NZ_JH109152.1
Methylocystis ATCC 49242	NZ_AEVM01000001.1
Methylomicrobium album BG8	NZ_AFJF01000001.1
Methylophaga aminisulfidivorans MP	NZ_AFIG01000001.1
Methylophaga thiooxidans DMS010	NZ_GG657882.1
Methylophilales bacterium HTCC2181	NZ_AAUX01000001.1
Methylosinus trichosporium OB3b	NZ_ADVE01000001.1
Methyloversatilis universalis FAM5	NZ_AFHG01000001.1

Micrococcus luteus NCTC 2665	NZ_CABC01000001.1
Micrococcus luteus SK58	NZ_ADCD01000001.1
Microcoleus chthonoplastes PCC 7420	NZ_DS989841.1
Microcoleus vaginatus FGP 2	NZ_AFJC01000001.1
Micromonospora ATCC 39149	NZ_GG657738.1
Microscilla marina ATCC 23134	NZ_AAWS01000001.1
Mitsuokella multacida DSM 20544	NZ_GG697141.2
Mobiluncus curtisii ATCC 35241	NZ_GL385912.1
Mobiluncus curtisii ATCC 51333	NZ_GL622340.1
Mobiluncus curtisii holmesii ATCC 35242	NZ_GL622346.1
Mobiluncus mulieris 28 1	NZ_ADBR01000001.1
Mobiluncus mulieris ATCC 35239	NZ_GL405260.1
Mobiluncus mulieris ATCC 35243	NZ_GG668518.1
Mobiluncus mulieris FB024 16	NZ_AEGV01000001.1
Moritella PE36	NZ_ABCQ01000001.1
Mucilaginibacter paludis DSM 18603	NZ_AEIH01000001.1
Mycobacterium avium ATCC 25291	NZ_ACFI01000001.1
Mycobacterium colombiense CECT 3035	NZ_AFVW01000001.1
Mycobacterium intracellulare ATCC 13950	NZ_ABIN01000001.1
Mycobacterium kansasii ATCC 12478	NZ_CM000636.3
Mycobacterium massiliense CCUG 48898	NZ_AHAR01000001.1
Mycobacterium parascrofulaceum ATCC BAA 614	NZ_GG770553.1
Mycobacterium thermoresistibile ATCC 19527	NZ_AGVE01000001.1
Mycobacterium tuberculosis 02 1987	NZ_DS985180.1
Mycobacterium tuberculosis 210	NZ_ADAB01000001.1
Mycobacterium tuberculosis 94 M4241A	NZ_DS985170.1
Mycobacterium tuberculosis 98 R604 INH RIF EM	NZ_DS999515.1
Mycobacterium tuberculosis CPHL A	NZ_GG663497.1
Mycobacterium tuberculosis C	NZ_CH482373.1
Mycobacterium tuberculosis EAS054	NZ_DS985164.1
Mycobacterium tuberculosis GM 1503	NZ_DS986655.1
Mycobacterium tuberculosis H37Ra	NZ_AAYK01000001.1
Mycobacterium tuberculosis Haarlem	NZ_DS016976.1
Mycobacterium tuberculosis K85	NZ_GG663503.1
Mycobacterium tuberculosis KZN 4207	NZ_CM000787.2
Mycobacterium tuberculosis KZN 605	NZ_GG745507.1
Mycobacterium tuberculosis KZN R506	NZ_CM000789.2
Mycobacterium tuberculosis KZN V2475	NZ_CM000788.2
Mycobacterium tuberculosis SUMu001	NZ_GL503107.1
Mycobacterium tuberculosis SUMu002	NZ_GL544642.1
Mycobacterium tuberculosis SUMu003	NZ_GL544682.1
Mycobacterium tuberculosis SUMu004	NZ_GL544745.1
Mycobacterium tuberculosis SUMu005	NZ_GL544809.1
Mycobacterium tuberculosis SUMu006	NZ_GL544864.1
Mycobacterium tuberculosis SUMu007	NZ_GL544922.1
Mycobacterium tuberculosis SUMu008	NZ_GL544963.1
Mycobacterium tuberculosis SUMu009	NZ_GL545004.1
Mycobacterium tuberculosis SUMu010	NZ_GL545050.1
Mycobacterium tuberculosis SUMu011	NZ_GL545102.1
Mycobacterium tuberculosis SUMu012	NZ_GL545158.1
Mycobacterium tuberculosis T17	NZ_DS986672.1
Mycobacterium tuberculosis T46	NZ_GG663489.1
Mycobacterium tuberculosis T85	NZ_DS985147.1
Mycobacterium tuberculosis T92	NZ_DS985116.1
Mycoplasma alligatoris A21JP2	NZ_ADNC01000001.1
Mycoplasma anatis 1340	NZ_AFVJ01000001.1
Mycoplasma columbinum SF7	NZ_AFXA01000001.1

Mycoplasma genitalium G37	NZ_AAGX01000001.1
Mycoplasma iowae 695	NZ_AGFP01000001.1
Mycoplasma ovipneumoniae SC01	NZ_AFHO01000001.1
Natrinema pellirubrum DSM 15624	NZ_AGIN01000001.1
Natronobacterium gregoryi SP2	NZ_AGIM01000001.1
Neisseria bacilliformis ATCC BAA 1200	NZ_GL878494.1
Neisseria cinerea ATCC 14685	NZ_ACDY02000001.1
Neisseria elongata glycolytica ATCC 29315	NZ_ADBF01000001.1
Neisseria flavescens NRL30031 H210	NZ_ACEN01000001.1
Neisseria flavescens SK114	NZ_ACQV01000001.1
Neisseria gonorrhoeae 1291	NZ_DS999917.1
Neisseria gonorrhoeae 35 02	NZ_DS999959.1
Neisseria gonorrhoeae DGI18	NZ_EQ972648.1
Neisseria gonorrhoeae DGI2	NZ_GG749016.1
Neisseria gonorrhoeae F62	NZ_GG749348.1
Neisseria gonorrhoeae FA19	NZ_EQ972745.1
Neisseria gonorrhoeae FA6140	NZ_EQ972690.1
Neisseria gonorrhoeae MS11	NZ_EQ972789.1
Neisseria gonorrhoeae PID18	NZ_EQ972834.1
Neisseria gonorrhoeae PID1	NZ_EQ972883.1
Neisseria gonorrhoeae PID24 1	NZ_EQ972931.1
Neisseria gonorrhoeae PID332	NZ_EQ972985.1
Neisseria gonorrhoeae SK 92 679	NZ_EQ973032.1
Neisseria gonorrhoeae SK 93 1035	NZ_EQ973079.1
Neisseria GT4A CT1	NZ_JH164964.1
Neisseria lactamica ATCC 23970	NZ_ACEQ02000001.1
Neisseria macacae ATCC 33926	NZ_GL985618.1
Neisseria meningitidis ATCC 13091	NZ_GL397187.1
Neisseria mucosa ATCC 25996	NZ_ACDX02000001.1
Neisseria mucosa C102	NZ_GL635793.1
Neisseria oral taxon 014 F0314	NZ_GL349411.1
Neisseria polysaccharea ATCC 43768	NZ_ADBE01000001.1
Neisseria shayeganii 871	NZ_JH164926.1
Neisseria sicca ATCC 29256	NZ_ACKO02000001.1
Neisseria subflava NJ9703	NZ_ACEO02000001.1
Neisseria wadsworthii 9715	NZ_JH165159.1
Neisseria weaveri LMG 5135	NZ_AFWQ01000001.1
Neptuniibacter caesariensis	NZ_CH724125.1
Nitrobacter Nb 311A	NZ_CH672416.1
Nitrococcus mobilis Nb 231	NZ_CH672427.1
Nitrosococcus oceani AFC27	NZ_DS995300.1
Nitrosopumilus MY1	NZ_AFPU01000001.1
Nocardioidaceae bacterium Broad 1	NZ_GL873260.1
Nodularia spumigena CCY9414	NZ_AAVW01000001.1
Novosphingobium nitrogenifigens DSM 19370	NZ_GL876925.1
Novosphingobium pentaromativorans US6 1	NZ_AGFM01000001.1
Oceanibulbus indolifex HEL 45	NZ_ABID01000001.1
Oceanicaulis alexandrii HTCC2633	NZ_CH672428.1
Oceanicola batsensis HTCC2597	NZ_CH724131.1
Oceanicola granulosus HTCC2516	NZ_CH724107.1
Ochrobactrum intermedium LMG 3301	NZ_ACQA01000001.1
Octadecabacter antarcticus 238	NZ_DS990628.1
Octadecabacter antarcticus 307	NZ_DS990574.1
Oenococcus oeni ATCC BAA 1163	NZ_AAUV01000001.1
Oenococcus oeni AWRIB429	NZ_ACSE01000001.1
Olsenella oral taxon 809 F0356	NZ_JH376563.1
Opitutaceae bacterium TAV2	NZ_ABEA02000001.1

Oribacterium ACB1	NZ_JH414495.1
Oribacterium ACB7	NZ_JH414504.1
Oribacterium oral taxon 078 F0262	NZ_GG729933.1
Oribacterium oral taxon 108 F0425	NZ_AFIH01000001.1
Oribacterium sinus F0268	NZ_GG668533.1
Ornithinibacillus TW25	NZ_AEWH01000001.1
Oscillatoria PCC 6506	NZ_CACA01000001.1
Oscillochloris trichoides DG6	NZ_GL501400.1
Oxalobacteraceae bacterium IMCC9480	NZ_AEPR01000001.1
Oxalobacter formigenes HOxBLS	NZ_GG658151.1
Oxalobacter formigenes OXCC13	NZ_GG658170.1
Paenibacillus curdlanolyticus YK9	NZ_AEDD01000001.1
Paenibacillus elgii B69	NZ_AFHW01000001.1
Paenibacillus HGF5	NZ_AEXS01000001.1
Paenibacillus HGF7	NZ_AFDH01000001.1
Paenibacillus lactis 154	NZ_AGIP01000001.1
Paenibacillus larvae B 3650	NZ_ADZY02000001.1
Paenibacillus larvae BRL 230010	NZ_CH981372.1
Paenibacillus oral taxon 786 D14	NZ_GG695970.1
Paenibacillus vortex V453	NZ_ADHJ01000001.1
Pantoea aB	NZ_AEDL01000001.1
Parabacteroides D13	NZ_GG698738.1
Parabacteroides johnsonii DSM 18315	NZ_DS996440.1
Parabacteroides merdae ATCC 43184	NZ_DS264460.1
Parachlamydia acanthamoebae Hall s coccus	NZ_ACZE01000001.1
Paracoccus TRP	NZ_AEPN01000001.1
Paraprevotella clara YIT 11840	NZ_JH376577.1
Paraprevotella xylaniphila YIT 11841	NZ_GL883805.1
Parascardovia denticolens DSM 10105	NZ_CM001148.1
Parascardovia denticolens F0305	NZ_GG770319.1
Parasutterella excrementihominis YIT 11859	NZ_GL883674.1
Parvimonas micra ATCC 33270	NZ_DS483504.1
Parvimonas oral taxon 110 F0139	NZ_AFII01000001.1
Parvimonas oral taxon 393 F0440	NZ_AFUS01000001.1
Pasteurella dagmatis ATCC 43325	NZ_GG704810.1
Patulibacter I11	NZ_AGUD01000001.1
Pectobacterium carotovorum brasiliensis PBR1692	NZ_ABVX01000001.1
Pectobacterium carotovorum WPP14	NZ_ABVY01000001.1
Pediococcus acidilactici 7 4	NZ_GG730083.1
Pediococcus acidilactici DSM 20284	NZ_GL397067.1
Pedobacter BAL39	NZ_ABCM01000001.1
Pelagibaca bermudensis HTCC2601	NZ_DS022276.1
Peptoniphilus duerdenii ATCC BAA 1640	NZ_GL397071.1
Peptoniphilus harei ACS 146 V Sch2b	NZ_AENP01000001.1
Peptoniphilus indolicus ATCC 29427	NZ_JH165061.1
Peptoniphilus lacrimalis 315 B	NZ_ADDO01000001.1
Peptoniphilus oral taxon 375 F0436	NZ_AFUH01000001.1
Peptoniphilus oral taxon 386 F0131	NZ_GL349422.1
Peptoniphilus oral taxon 836 F0141	NZ_AEAA01000001.1
Peptostreptococcus anaerobius 653 L	NZ_ADJN01000001.1
Peptostreptococcus stomatis DSM 17678	NZ_ADGQ01000001.1
Phaeobacter gallaeciensis 2 10	NZ_DS544874.1
Phaeobacter gallaeciensis BS107	NZ_DS544878.1
Phascolarctobacterium YIT 12067	NZ_GL830843.1
Photobacterium angustum S14	NZ_CH902599.1
Photobacterium damselae CIP 102761	NZ_ADBS01000001.1
Photobacterium leiognathi mandapamensis svers 1 1	NZ_DF093593.1

Photobacterium profundum 3TCK	NZ_CH724134.1
Photobacterium SKA34	NZ_CH724141.1
Planctomyces maris DSM 8797	NZ_ABCE01000001.1
Planococcus donghaensis MPA1U2	NZ_AEPB01000001.1
Plautia stali symbiont	NZ_BABY01000001.1
Plesiocystis pacifica SIR 1	NZ_ABCS01000001.1
Polaribacter irgensii 23 P	NZ_CH724148.1
Polaribacter MED152	NZ_CH902588.1
Porphyromonas asaccharolytica PR426713P I	NZ_AENO01000001.1
Porphyromonas endodontalis ATCC 35406	NZ_ACNN01000001.1
Porphyromonas uenonis 60 3	NZ_ACLR01000001.1
Prevotella amnii CRIS 21A A	NZ_ADFQ01000001.1
Prevotella bergensis DSM 17361	NZ_GG704780.1
Prevotella bivia JCVIHMP010	NZ_ADFO01000001.1
Prevotella bryantii B14	NZ_ADWO01000001.1
Prevotella buccae ATCC 33574	NZ_GL586311.1
Prevotella buccae D17	NZ_GG739926.1
Prevotella buccalis ATCC 35310	NZ_ADEG01000001.1
Prevotella C561	NZ_JH114139.1
Prevotella copri DSM 18205	NZ_GG703852.1
Prevotella dentalis DSM 3688	NZ_GL982488.1
Prevotella denticola CRIS 18C A	NZ_AEXO01000001.1
Prevotella disiens FB035 09AN	NZ_AEDO01000001.1
Prevotella histicola F0411	NZ_JH376762.1
Prevotella marshii DSM 16973	NZ_GL397214.1
Prevotella melaninogenica D18	NZ_GG740010.1
Prevotella multiformis DSM 16608	NZ_GL872282.1
Prevotella multisaccharivorax DSM 17128	NZ_GL945015.1
Prevotella nigrescens ATCC 33563	NZ_GL982464.1
Prevotella oralis ATCC 33269	NZ_GL833116.1
Prevotella oral taxon 299 F0039	NZ_GG740056.1
Prevotella oral taxon 302 F0323	NZ_JH376827.1
Prevotella oral taxon 317 F0108	NZ_GG740072.1
Prevotella oral taxon 472 F0295	NZ_GG704824.1
Prevotella oris C735	NZ_GL349564.1
Prevotella oris F0302	NZ_GG703883.1
Prevotella oulorum F0390	NZ_JH114215.1
Prevotella pallens ATCC 700821	NZ_GL982513.1
Prevotella salivae DSM 15606	NZ_GL629647.1
Prevotella stercorea DSM 18206	NZ_JH379330.1
Prevotella tannerae ATCC 51259	NZ_GG700642.1
Prevotella timonensis CRIS 5C B1	NZ_ADEF01000001.1
Prevotella veroralis F0319	NZ_GG698712.1
Prochlorococcus marinus MIT 9202	NZ_DS999537.1
Propionibacterium 409 HC1	NZ_AFIK01000001.1
Propionibacterium 434 HC2	NZ_AFIL01000001.1
Propionibacterium 5 U 42AFAA	NZ_JH376566.1
Propionibacterium acnes J139	NZ_ADFS01000001.1
Propionibacterium acnes J165	NZ_ADJL01000001.1
Propionibacterium acnes SK187	NZ_ADJM01000001.1
Propionibacterium avidum ATCC 25577	NZ_JH165054.1
Propionibacterium CC003 HC2	NZ_AFUK01000001.1
Proteus mirabilis ATCC 29906	NZ_GG668576.1
Proteus penneri ATCC 35198	NZ_GG661994.1
Providencia alcalifaciens DSM 30120	NZ_ABXW01000001.1
Providencia rettgeri DSM 1131	NZ_GG705262.1
Providencia rustigianii DSM 4541	NZ_GG703817.1

Providencia stuartii ATCC 25827	NZ_DS607633.1
Pseudoalteromonas BSi20311	NZ_BADU01000001.1
Pseudoalteromonas BSi20429	NZ_BADV01000001.1
Pseudoalteromonas BSi20439	NZ_BADW01000001.1
Pseudoalteromonas BSi20480	NZ_BADX01000001.1
Pseudoalteromonas BSi20495	NZ_BADY01000001.1
Pseudoalteromonas BSi20652	NZ_BADT01000001.1
Pseudoalteromonas haloplanktis ANT 505	NZ_ADOP01000001.1
Pseudoalteromonas tunicata D2	NZ_CH959301.1
Pseudomonas 2 1 26	NZ_JH376461.1
Pseudomonas aeruginosa 2192	NZ_CH482384.1
Pseudomonas aeruginosa 39016	NZ_CM001020.1
Pseudomonas aeruginosa C3719	NZ_CH482383.1
Pseudomonas aeruginosa PAb1	NZ_ABKZ01000001.1
Pseudomonas aeruginosa PACS2	NZ_AAQW01000001.1
Pseudomonas fluorescens WH6	NZ_CM001025.1
Pseudomonas psychrotolerans L19	NZ_AHBD01000001.1
Pseudomonas savastanoi NCPPB 3335	NZ_GG774590.1
Pseudomonas syringae 642	NZ_ADGB01000001.1
Pseudomonas syringae aesculi 2250	NZ_GG700072.1
Pseudomonas syringae aesculi NCPPB3681	NZ_GG699515.1
Pseudomonas syringae FF5	NZ_GG700436.1
Pseudomonas syringae oryzae 1 6	NZ_DS996945.1
Pseudomonas syringae tabaci ATCC 11528	NZ_GG699444.1
Pseudomonas syringae tomato K40	NZ_ADFY01000001.1
Pseudomonas syringae tomato Max13	NZ_ADFZ01000001.1
Pseudomonas syringae tomato NCPPB 1108	NZ_ADGA01000001.1
Pseudomonas syringae tomato T1	NZ_ABSM01000001.1
Pseudomonas TJI 51	NZ_AEWE01000001.1
Pseudomonas UK4	NZ_ACOQ01000001.1
Pseudonocardia P1	NZ_ADUJ01000001.1
Pseudoramibacter alactolyticus ATCC 23263	NZ_GL622359.1
Pseudovibrio JE062	NZ_DS996805.1
Psychrobacter 1501 2011	NZ_GL892048.1
Psychroflexus torquis ATCC 700755	NZ_CH959305.1
Psychromonas CNPT3	NZ_CH902574.1
Pyramidobacter piscolens W5455	NZ_ADFP01000001.1
Ralstonia 5 2 56FAA	NZ_JH165001.1
Ralstonia 5 7 47FAA	NZ_GL520217.1
Ralstonia solanacearum UW551	NZ_AAKL01000001.1
Raphidiopsis brookii D9	NZ_ACYB01000001.1
Reinekea blandensis MED297	NZ_CH724149.1
Rheinheimera A13L	NZ_AFHI01000001.1
Rhizobium etli 8C 3	NZ_ABRA01000001.1
Rhizobium etli Brasil 5	NZ_ABQZ01000001.1
Rhizobium etli CIAT 894	NZ_ABRD01000001.1
Rhizobium etli GR56	NZ_ABRB01000001.1
Rhizobium etli IE4771	NZ_ABRC01000001.1
Rhizobium etli Kim 5	NZ_ABQY01000001.1
Rhodanobacter 2APBS1	NZ_AGIL01000001.1
Rhodobacteraceae bacterium KLH11	NZ_DS999531.1
Rhodobacterales bacterium HTCC2083	NZ_DS995276.1
Rhodobacterales bacterium HTCC2150	NZ_AAXZ01000001.1
Rhodobacterales bacterium HTCC2255	NZ_DS022282.1
Rhodobacterales bacterium Y4I	NZ_DS995281.1
Rhodobacter sphaeroides WS8N	NZ_CM001161.1
Rhodobacter SW2	NZ_ACYY01000001.1

Rhodococcus equi ATCC 33707	NZ_CM001149.1
Rhodococcus erythropolis SK121	NZ_ACNO01000001.1
Rhodococcus pyridinivorans AK37	NZ_AHBW01000001.1
Rickettsia endosymbiont of Ixodes scapularis	NZ_CM000770.1
Rickettsia sibirica 246	NZ_AABW01000001.1
Rickettsiella grylli	NZ_AAQJ02000001.1
Roseburia intestinalis L1 82	NZ_GG692713.1
Roseburia inulinivorans DSM 16841	NZ_ACFY01000001.1
Roseibium TrichSKD4	NZ_GL476300.1
Roseobacter AzwK 3b	NZ_ABCR01000001.1
Roseobacter CCS2	NZ_AAYB01000001.1
Roseobacter GAI101	NZ_DS999213.1
Roseobacter MED193	NZ_CH902583.1
Roseobacter SK209 2 6	NZ_AAYC01000001.1
Roseomonas cervicalis ATCC 49957	NZ_GG770777.1
Roseovarius 217	NZ_CH902584.1
Roseovarius nubinhibens ISM	NZ_CH724156.1
Roseovarius TM1035	NZ_ABCL01000001.1
Rothia dentocariosa M567	NZ_GL379574.1
Rothia mucilaginosa ATCC 25296	NZ_ACVO01000001.1
Rubrivivax benzoatilyticus JA2	NZ_AEWG01000001.1
Ruegeria R11	NZ_DS999054.1
Ruegeria TW15	NZ_AEYW01000001.1
Ruminococcaceae bacterium D16	NZ_GL890784.1
Ruminococcus 5 1 39BFAA	NZ_GG696045.1
Ruminococcus albus 8	NZ_ADKM02000001.1
Ruminococcus flavefaciens FD 1	NZ_ACOK01000001.1
Ruminococcus gnavus ATCC 29149	NZ_AAYG02000001.1
Ruminococcus lactaris ATCC 29176	NZ_DS990163.1
Ruminococcus obeum ATCC 29174	NZ_DS264289.1
Ruminococcus torques ATCC 27756	NZ_DS264343.1
Saccharomonospora azurea NA 128	NZ_AGIU01000001.1
Saccharomonospora paurometabolica YIM 90007	NZ_AGIT01000001.1
Saccharopolyspora erythraea NRRL 2338	NZ_ABFV01000001.1
Saccharopolyspora spinosa NRRL 18395	NZ_GL877878.1
Sagittula stellata E 37	NZ_AAYA01000001.1
Salinisphaera shabanensis E1L3A	NZ_AFNV01000001.1
Salmonella enterica serovar 4 5 12 i CVM23701	NZ_ABAO01000001.3
Salmonella enterica serovar Hadar RI 05P066	NZ_ABFG01000001.1
Salmonella enterica serovar Heidelberg SL486	NZ_ABEL01000001.1
Salmonella enterica serovar Javiana GA MM04042433	NZ_ABEH02000001.1
Salmonella enterica serovar Kentucky CDC 191	NZ_ABEI01000001.1
Salmonella enterica serovar Kentucky CVM29188	NZ_ABAK02000001.1
Salmonella enterica serovar Newport SL317	NZ_ABEW01000001.1
Salmonella enterica serovar Saintpaul SARA23	NZ_ABAM02000001.1
Salmonella enterica serovar Saintpaul SARA29	NZ_ABAN01000001.3
Salmonella enterica serovar Schwarzengrund SL480	NZ_ABEJ01000001.1
Salmonella enterica serovar Tennessee CDC07 0191	NZ_ACBF01000001.1
Salmonella enterica serovar Virchow SL491	NZ_ABFH02000001.1
Salmonella enterica serovar Weltevreden HI N05 537	NZ_ABFF01000001.1
SAR 116 cluster alpha proteobacterium HIMB100	NZ_AFXB01000001.1
Scardovia inopinata F0304	NZ_GG770225.1
Segniliparus rugosus ATCC BAA 974	NZ_GL622754.1
Selenomonas artemidis F0399	NZ_GL638127.1
Selenomonas flueggei ATCC 43531	NZ_GG694006.1
Selenomonas infelix ATCC 43532	NZ_JH376797.1
Selenomonas noxia ATCC 43541	NZ_GG749278.1

Selenomonas oral taxon 137 F0430	NZ_AENV01000001.1
Selenomonas oral taxon 149 67H29BP	NZ_GL397087.1
Selenomonas sputigena ATCC 35185	NZ_GG698596.1
Serratia odorifera 4Rx13	NZ_ADBX01000001.1
Serratia odorifera DSM 4582	NZ_GG753567.1
Serratia symbiotica Tucson	NZ_GL636097.1
Shewanella baltica OS183	NZ_AECY01000001.1
Shewanella benthica KT99	NZ_ABIC01000001.1
Shewanella HN 41	NZ_AFOZ01000001.1
Shigella D9	NZ_GG657384.1
Shigella dysenteriae 1012	NZ_AAMJ02000001.1
Shigella dysenteriae 1617	NZ_ADUT01000001.1
Shuttleworthia satelles DSM 14600	NZ_GG665866.1
Silicibacter lacuscaerulensis ITI 1157	NZ_GG704595.1
Silicibacter TrichCH4B	NZ_GG703514.1
Simonsiella muelleri ATCC 29453	NZ_GG770234.1
Slackia exigua ATCC 700122	NZ_GG700630.1
Solobacterium moorei F0204	NZ_GL637642.1
Sphingobacterium spiritivorum ATCC 33300	NZ_GG668630.1
Sphingobacterium spiritivorum ATCC 33861	NZ_GL379770.1
Sphingomonas KC8	NZ_AFMP01000001.1
Sphingomonas S17	NZ_AFGG01000001.1
Sphingomonas SKA58	NZ_CH959306.1
Spiroplasma melliferum KC3	NZ_AGBZ01000001.1
Sporosarcina newyorkensis	NZ_GL982997.1
Staphylococcus aureus 132	NZ_ACOT01000001.1
Staphylococcus aureus 55 2053	NZ_GG700533.1
Staphylococcus aureus 58 424	NZ_GG749054.1
Staphylococcus aureus 65 1322	NZ_GG700559.1
Staphylococcus aureus 68 397	NZ_GG700574.1
Staphylococcus aureus 930918 3	NZ_ABFA01000001.1
Staphylococcus aureus A017934 97	NZ_GG731495.1
Staphylococcus aureus A10102	NZ_ACSO01000001.1
Staphylococcus aureus A5937	NZ_ACKC01000001.1
Staphylococcus aureus A5948	NZ_ACKD01000001.1
Staphylococcus aureus A6224	NZ_ACKE01000001.1
Staphylococcus aureus A6300	NZ_ACKF01000001.1
Staphylococcus aureus A8115	NZ_ACKG01000001.1
Staphylococcus aureus A8117	NZ_ACYO01000001.1
Staphylococcus aureus A8796	NZ_ADJJ01000001.1
Staphylococcus aureus A8819	NZ_ADJK01000001.1
Staphylococcus aureus A9299	NZ_ACKH01000001.1
Staphylococcus aureus A9635	NZ_ACKI01000001.1
Staphylococcus aureus A9719	NZ_ACKJ01000001.1
Staphylococcus aureus A9754	NZ_ADJI01000001.1
Staphylococcus aureus A9763	NZ_ACKK01000001.1
Staphylococcus aureus A9765	NZ_ACSN01000001.1
Staphylococcus aureus A9781	NZ_ACKL01000001.1
Staphylococcus aureus ATCC 51811	NZ_GG774480.1
Staphylococcus aureus ATCC BAA 39	NZ_GL397103.1
Staphylococcus aureus Btn1260	NZ_GG730256.1
Staphylococcus aureus C101	NZ_GG730120.1
Staphylococcus aureus C160	NZ_GG730273.1
Staphylococcus aureus C427	NZ_GG730141.1
Staphylococcus aureus CF Marseille	NZ_CABA01000001.1
Staphylococcus aureus D139	NZ_GG730159.1
Staphylococcus aureus D30	NZ_ABFB01000001.1

Staphylococcus aureus E1410	NZ_GG700597.1
Staphylococcus aureus EMRSA16	NZ_GG770513.1
Staphylococcus aureus H19	NZ_GG730335.1
Staphylococcus aureus JKD6009	NZ_ABSA01000001.1
Staphylococcus aureus M1015	NZ_GG748996.1
Staphylococcus aureus M809	NZ_GG749299.1
Staphylococcus aureus M876	NZ_GG700614.1
Staphylococcus aureus M899	NZ_GG730190.1
Staphylococcus aureus MN8	NZ_CM000952.1
Staphylococcus aureus MR1	NZ_ACZQ01000001.1
Staphylococcus aureus Mu50 omega	NZ_BABM01000001.1
Staphylococcus aureus TCH130	NZ_GG698158.1
Staphylococcus aureus TCH70	NZ_GL538356.1
Staphylococcus aureus USA300 TCH959	NZ_GG697985.1
Staphylococcus aureus WBG10049	NZ_GG730208.1
Staphylococcus aureus WW2703 97	NZ_GG730220.1
Staphylococcus capitis SK14	NZ_ACFR01000001.1
Staphylococcus caprae C87	NZ_GL545267.1
Staphylococcus epidermidis BCM HMP0060	NZ_GG696727.1
Staphylococcus epidermidis M23864 W1	NZ_GG696773.1
Staphylococcus epidermidis M23864 W2 grey	NZ_GG749255.1
Staphylococcus epidermidis SK135	NZ_ADEY01000001.1
Staphylococcus epidermidis W23144	NZ_GG696799.1
Staphylococcus hominis C80	NZ_GL545252.1
Staphylococcus hominis SK119	NZ_ACLP01000001.1
Staphylococcus lugdunensis M23590	NZ_GL622351.1
Staphylococcus simiae CCM 7213	NZ_AEUN01000001.1
Staphylococcus warneri L37603	NZ_ACPZ01000001.1
Stenotrophomonas SKA14	NZ_DS999412.1
Stigmatella aurantiaca DW4 3 1	NZ_AAMD01000001.1
Streptococcus 2 1 36FAA	NZ_GG704939.1
Streptococcus agalactiae 18RS21	NZ_AAJO01000001.1
Streptococcus agalactiae 515	NZ_AAJP01000001.1
Streptococcus agalactiae ATCC 13813	NZ_AEQQ01000001.1
Streptococcus agalactiae CJB111	NZ_AAJQ01000001.1
Streptococcus agalactiae COH1	NZ_AAJR01000001.1
Streptococcus agalactiae H36B	NZ_AAJS01000001.1
Streptococcus anginosus 1 2 62CV	NZ_GL636615.1
Streptococcus anginosus F0211	NZ_AECT01000001.1
Streptococcus anginosus SK52	NZ_AFIM01000001.1
Streptococcus australis ATCC 700641	NZ_GL636091.1
Streptococcus bovis ATCC 700338	NZ_GL397128.1
Streptococcus C150	NZ_GL698449.1
Streptococcus C300	NZ_GL732466.1
Streptococcus constellatus pharyngis SK1060	NZ_AFUP01000001.1
Streptococcus criceti	NZ_AEUV02000001.1
Streptococcus cristatus ATCC 51100	NZ_GL732518.1
Streptococcus downei F0415	NZ_AEKN01000001.1
Streptococcus equinus ATCC 9812	NZ_GL698429.1
Streptococcus gallolyticus TX20005	NZ_GL397173.1
Streptococcus ictaluri 707 05	NZ_AEUX02000001.1
Streptococcus infantarius ATCC BAA 102	NZ_DS572674.1
Streptococcus infantis ATCC 700779	NZ_GL732439.1
Streptococcus infantis SK1076	NZ_AFNN01000001.1
Streptococcus infantis SK1302	NZ_AEDY01000001.1
Streptococcus M143	NZ_GG730092.1
Streptococcus M334	NZ_GL732486.1

Streptococcus macacae NCTC 11558	NZ_AEUW02000001.1
Streptococcus mitis ATCC 6249	NZ_GL397179.1
Streptococcus mitis NCTC 12261	NZ_AEDX01000001.1
Streptococcus mitis SK321	NZ_AEDT01000001.1
Streptococcus mitis SK564	NZ_AEDU01000001.1
Streptococcus mitis SK597	NZ_AEDV01000001.1
Streptococcus oralis ATCC 35037	NZ_AEDW01000001.1
Streptococcus oralis	NZ_GG749268.1
Streptococcus oral taxon 056 F0418	NZ_AFQU01000001.1
Streptococcus oral taxon 058 F0407	NZ_JH378872.1
Streptococcus oral taxon 071 73H25AP	NZ_GL397254.1
Streptococcus parasanguinis ATCC 903	NZ_GL732449.1
Streptococcus parasanguinis F0405	NZ_AEKM01000001.1
Streptococcus parauberis NCFD 2020	NZ_AEUT02000001.1
Streptococcus peroris ATCC 700780	NZ_GL732463.1
Streptococcus pneumoniae BS397	NZ_ABWC01000001.1
Streptococcus pneumoniae BS455	NZ_ADHN01000001.1
Streptococcus pneumoniae BS457	NZ_ABWB01000001.1
Streptococcus pneumoniae BS458	NZ_ABWA01000001.1
Streptococcus pneumoniae Canada MDR 19A	NZ_ACNU01000001.1
Streptococcus pneumoniae Canada MDR 19F	NZ_ACNV01000001.1
Streptococcus pneumoniae CCRI 1974M2	NZ_ABZT01000001.1
Streptococcus pneumoniae CCRI 1974	NZ_ABZC01000001.1
Streptococcus pneumoniae CDC0288 04	NZ_ABGF01000001.1
Streptococcus pneumoniae CDC1087 00	NZ_ABFT01000001.1
Streptococcus pneumoniae CDC1873 00	NZ_ABFS01000001.1
Streptococcus pneumoniae CDC3059 06	NZ_ABGG01000001.1
Streptococcus pneumoniae MLV 016	NZ_ABGH01000001.1
Streptococcus pneumoniae SP11 BS70	NZ_ABAC01000001.1
Streptococcus pneumoniae SP14 BS292	NZ_ABWQ01000001.1
Streptococcus pneumoniae SP14 BS69	NZ_ABAD01000001.1
Streptococcus pneumoniae SP18 BS74	NZ_ABAE01000001.1
Streptococcus pneumoniae SP195	NZ_ABGE01000001.1
Streptococcus pneumoniae SP19 BS75	NZ_ABAF01000001.1
Streptococcus pneumoniae SP23 BS72	NZ_ABAG01000001.1
Streptococcus pneumoniae SP3 BS71	NZ_AAZZ01000001.1
Streptococcus pneumoniae SP6 BS73	NZ_ABAA01000001.1
Streptococcus pneumoniae SP9 BS68	NZ_ABAB01000001.1
Streptococcus pneumoniae SP9v BS293	NZ_ABWU01000001.1
Streptococcus pneumoniae TIGR4	NZ_AAGY02000001.1
Streptococcus porcinus Jelinkova 176	NZ_AEUU02000001.1
Streptococcus pseudoporcinus SPIN 20026	NZ_AENS01000001.1
Streptococcus pyogenes ATCC 10782	NZ_GL397225.1
Streptococcus pyogenes M49 591	NZ_AAFV01000001.1
Streptococcus salivarius SK126	NZ_ACLO01000001.1
Streptococcus sanguinis ATCC 49296	NZ_GL622183.1
Streptococcus sanguinis VMC66	NZ_GL831107.1
Streptococcus suis 05HAH33	NZ_AARD01000001.1
Streptococcus suis 89 1591	NZ_AAFA03000001.1
Streptococcus urinalis 2285 97	NZ_AEUZ02000001.1
Streptococcus vestibularis ATCC 49124	NZ_GL831112.1
Streptococcus vestibularis F0396	NZ_AEKO01000001.1
Streptomyces AA4	NZ_GG657746.1
Streptomyces ACT 1	NZ_GL877172.1
Streptomyces albus J1074	NZ_DS999645.1
Streptomyces clavuligerus ATCC 27064	NZ_CM000913.1
Streptomyces clavuligerus ATCC 27064	NZ_DS570624.1

Streptomyces clavuligerus ATCC 27064	NZ_CM001015.1
Streptomyces C	NZ_GG657750.1
Streptomyces e14	NZ_GG753626.1
Streptomyces ghanaensis ATCC 14672	NZ_DS999641.1
Streptomyces griseoaurantiacus M045	NZ_AEYX01000001.1
Streptomyces griseoflavus Tu4000	NZ_GG657758.1
Streptomyces hygroscopicus ATCC 53653	NZ_GG657754.1
Streptomyces lividans TK24	NZ_GG657756.1
Streptomyces Mg1	NZ_DS570384.1
Streptomyces pristinaespiralis ATCC 25486	NZ_CM000950.1
Streptomyces roseosporus NRRL 11379	NZ_DS999647.1
Streptomyces roseosporus NRRL 15998	NZ_DS999644.1
Streptomyces S4	NZ_FR873693.1
Streptomyces SA3 actF	NZ_ADXB01000001.1
Streptomyces SA3 actG	NZ_ADXA01000001.1
Streptomyces SPB74	NZ_GG770539.1
Streptomyces SPB78	NZ_GG657742.1
Streptomyces sviceus ATCC 29083	NZ_CM000951.1
Streptomyces Tu6071	NZ_CM001165.1
Streptomyces viridochromogenes DSM 40736	NZ_GG657757.1
Streptomyces W007	NZ_AGSW01000001.1
Streptomyces zinciresistens K42	NZ_AGBF01000001.1
Subdoligranulum 4 3 54A2FAA	NZ_JH414698.1
Subdoligranulum variabile DSM 15176	NZ_GG704769.1
Succinatimonas hippei YIT 12066	NZ_GL830939.1
Sulfitobacter EE 36	NZ_CH959310.1
Sulfitobacter NAS 14 1	NZ_CH959312.1
Sulfolobus solfataricus 98 2	NZ_ACUK01000001.1
Sulfurihydrogenibium yellowstonense SS 5	NZ_ABZS01000001.1
Sutterella wadsworthensis 3 1 45B	NZ_GL636540.1
Synechococcus BL107	NZ_DS022298.1
Synechococcus CB0101	NZ_ADXL01000001.1
Synechococcus CB0205	NZ_ADXM01000001.1
Synechococcus PCC 7335	NZ_DS989904.1
Synechococcus RS9916	NZ_DS022299.1
Synechococcus RS9917	NZ_CH724158.1
Synechococcus WH 5701	NZ_CH724159.1
Synechococcus WH 7805	NZ_CH724168.1
Synechococcus WH 8016	NZ_AGIK01000001.1
Synechococcus WH 8109	NZ_GG704594.1
Synergistes 3 1 syn1	NZ_JH414690.1
Tannerella 6 1 58FAA CT1	NZ_JH414766.1
Thalassiobium R2A62	NZ_GG697169.2
Thermaerobacter subterraneus DSM 13965	NZ_AENY01000001.1
Thermanaerovibrio velox DSM 12556	NZ_CM001377.1
Thermoanaerobacter ethanolicus CCSD1	NZ_ACXY01000001.1
Thermoanaerobacter ethanolicus JW 200	NZ_AEYS01000001.1
Thermoanaerobacter X561	NZ_ACXP02000001.1
Thermobacillus composti KWC4	NZ_AGFE01000001.1
Thermosinus carboxydivorans Nor1	NZ_AAWL01000001.1
Thermotogales bacterium mesG1 Ag 4 2	NZ_AEDC01000001.1
Thermus aquaticus Y51MC23	NZ_ABVK02000001.1
Thioalkalimicrobium aerophilum AL3	NZ_AGFA01000001.1
Thioalkalivibrio thiocyanoxidans ARh 4	NZ_AGFB01000001.1
Thiocapsa marina 5811	NZ_AFWV01000001.1
Thiocystis violascens DSM 198	NZ_AGFC01000001.1
Thiorhodococcus drewsii AZ1	NZ_AFWT01000001.1

Thiorhodospira sibirica ATCC 700588	NZ_AGFD01000001.1
Thiorhodovibrio 970	NZ_AFWS01000001.1
Treponema phagedenis F0421	NZ_GL637955.1
Treponema vincentii ATCC 35580	NZ_ACYH01000001.1
Turicibacter HGF1	NZ_AEXQ01000001.1
Turicibacter PC909	NZ_ADMN01000001.1
unidentified eubacterium SCB49	NZ_ABCO01000001.1
Ureaplasma parvum serovar 14 ATCC 33697	NZ_ABER01000001.1
Ureaplasma parvum serovar 1 ATCC 27813	NZ_ABES01000001.1
Ureaplasma parvum serovar 6 ATCC 27818	NZ_AAZQ01000001.1
Ureaplasma urealyticum serovar 11 ATCC 33695	NZ_AAZS01000001.1
Ureaplasma urealyticum serovar 12 ATCC 33696	NZ_AAZT01000001.1
Ureaplasma urealyticum serovar 13 ATCC 33698	NZ_ABEV01000001.1
Ureaplasma urealyticum serovar 2 ATCC 27814	NZ_ABFL02000001.1
Ureaplasma urealyticum serovar 4 ATCC 27816	NZ_AAYO02000001.1
Ureaplasma urealyticum serovar 5 ATCC 27817	NZ_AAZR01000001.1
Ureaplasma urealyticum serovar 7 ATCC 27819	NZ_AAYP01000001.1
Ureaplasma urealyticum serovar 8 ATCC 27618	NZ_AAYN02000001.1
Ureaplasma urealyticum serovar 9 ATCC 33175	NZ_AAYQ02000001.1
Veillonella 3 1 44	NZ_GG770199.1
Veillonella 6 1 27	NZ_GG770215.1
Veillonella atypica ACS 049 V Sch6	NZ_AEDR01000001.1
Veillonella atypica ACS 134 V Col7a	NZ_AEDS01000001.1
Veillonella dispar ATCC 17748	NZ_GG667604.1
Veillonella oral taxon 158 F0412	NZ_AENU01000001.1
Veillonella oral taxon 780 F0422	NZ_AFUJ01000001.1
Veillonella parvula ATCC 17745	NZ_ADFU01000001.1
Verminephrobacter At4	NZ_AFAL01000001.1
Verrucomicrobiae bacterium DG1235	NZ_DS990592.1
Verrucomicrobium spinosum DSM 4136	NZ_ABIZ01000001.1
Vibrio alginolyticus 12G01	NZ_CH902589.1
Vibrio alginolyticus 40B	NZ_ACZB01000001.1
Vibrio AND4	NZ_ABGR01000001.1
Vibrio brasiliensis LMG 20546	NZ_AEVS01000001.1
Vibrio caribbenthicus ATCC BAA 2122	NZ_AEIU01000001.1
Vibrio cholerae 12129 1	NZ_ACFQ01000001.1
Vibrio cholerae 1587	NZ_AAUR01000001.1
Vibrio cholerae 2740 80	NZ_AAUT01000001.1
Vibrio cholerae 623 39	NZ_DS178239.1
Vibrio cholerae AM 19226	NZ_DS265224.1
Vibrio cholerae B33	NZ_DS178044.1
Vibrio cholerae B33	NZ_ACHZ01000001.1
Vibrio cholerae bv albensis VL426	NZ_ACHV01000001.1
Vibrio cholerae BX 330286	NZ_ACIA01000001.1
Vibrio cholerae CIRS101	NZ_ACVW01000001.1
Vibrio cholerae CT 5369 93	NZ_ADAL01000001.1
Vibrio cholerae INDRE 91 1	NZ_ADAK01000001.1
Vibrio cholerae MAK 757	NZ_GG774578.1
Vibrio cholerae MO10	NZ_DS990136.1
Vibrio cholerae MZO 2	NZ_DS178165.1
Vibrio cholerae MZO 3	NZ_AAUU01000001.1
Vibrio cholerae NCTC 8457	NZ_DS177946.1
Vibrio cholerae RC27	NZ_ADAI01000001.1
Vibrio cholerae RC385	NZ_GG774555.1
Vibrio cholerae RC9	NZ_ACHX01000001.1
Vibrio cholerae TM 11079 80	NZ_ACHW01000001.1
Vibrio cholerae TMA 21	NZ_ACHY01000001.1

Vibrio cholerae V51	NZ_DS179714.1
Vibrio cholerae V52	NZ_AAKJ02000001.1
Vibrio coralliilyticus ATCC BAA 450	NZ_ACZN01000001.1
Vibrio Ex25	NZ_DS267808.1
Vibrio furnissii CIP 102972	NZ_ACZP01000001.1
Vibrio harveyi 1DA3	NZ_ACZC01000001.1
Vibrio harveyi HY01	NZ_DS179404.1
Vibrio ichthyoenteri ATCC 700023	NZ_AFWF01000001.1
Vibrio MED222	NZ_CH902606.1
Vibrio metschnikovii CIP 69 14	NZ_ACZO01000001.1
Vibrio mimicus MB 451	NZ_ADAF01000001.1
Vibrio mimicus VM223	NZ_ADAJ01000001.1
Vibrio mimicus VM573	NZ_ACYV01000001.1
Vibrio mimicus VM603	NZ_ACYU01000001.1
Vibrio N418	NZ_AFWD01000001.1
Vibrionales bacterium SWAT 3	NZ_AAZW01000001.1
Vibrio nigripulchritudo ATCC 27043	NZ_AFWJ01000001.1
Vibrio ordalii ATCC 33509	NZ_AEZC01000001.1
Vibrio orientalis CIP 102891	NZ_ACZV01000001.1
Vibrio parahaemolyticus 16	NZ_DS999325.1
Vibrio parahaemolyticus AN 5034	NZ_ACFO01000001.1
Vibrio parahaemolyticus AQ3810	NZ_DS179898.1
Vibrio parahaemolyticus AQ4037	NZ_ACFN01000001.1
Vibrio parahaemolyticus K5030	NZ_ACKB01000001.1
Vibrio parahaemolyticus Peru 466	NZ_ACFM01000001.1
Vibrio RC341	NZ_ACZT01000001.1
Vibrio RC586	NZ_ADBD01000001.1
Vibrio rotiferianus DAT722	NZ_AFAJ01000001.1
Vibrio scophthalmi LMG 19158	NZ_AFWE01000001.1
Vibrio shilonii AK1	NZ_ABCH01000001.1
Vibrio sinaloensis DSM 21326	NZ_AEVT01000001.1
Vibrio splendidus 12B01	NZ_CH724170.1
Vibrio tubiashii ATCC 19109	NZ_AFWI01000001.1
Victivallis vadensis ATCC BAA 548	NZ_ABDE02000001.1
Weissella cibaria KACC 11862	NZ_AEKT01000001.1
Weissella paramesenteroides ATCC 33313	NZ_GG697128.1
Wolbachia endosymbiont of Culex quinquefasciatus JHB	NZ_DS996929.1
Wolbachia endosymbiont of Drosophila ananassae	NZ_AAGB01000001.1
Wolbachia endosymbiont of Drosophila simulans	NZ_AAGC01000001.1
Wolbachia endosymbiont of Drosophila willistoni TSC 14030 0811 24	NZ_CH899776.1
Wolbachia endosymbiont of Muscidifurax uniraptor	NZ_ACFP01000001.1
Wolbachia endosymbiont wVitB of Nasonia vitripennis	NZ_GL883616.1
Xanthomonas campestris musacearum NCPPB4381	NZ_GG699329.1
Xanthomonas campestris vasculorum NCPPB702	NZ_GG699232.1
Xanthomonas fuscans aurantifolii ICPB 10535	NZ_ACPY01000001.1
Xanthomonas fuscans aurantifolii ICPB 11122	NZ_ACPX01000001.1
Xanthomonas gardneri ATCC 19865	NZ_AEQX01000001.1
Xanthomonas perforans 91 118	NZ_AEQW01000001.1
Xanthomonas vesicatoria ATCC 35937	NZ_AEQV01000001.1
Xylella fastidiosa Dixon	NZ_AAAL02000001.1
Xylella fastidiosa sandyi Ann 1	NZ_AAAM03000001.1
Yersinia aldovae ATCC 35236	NZ_ACCB01000001.1
Yersinia bercovieri ATCC 43970	NZ_AALC02000001.1
Yersinia frederiksenii ATCC 33641	NZ_AALE02000001.1
Yersinia intermedia ATCC 29909	NZ_AALF02000001.1
Yersinia kristensenii ATCC 33638	NZ_ACCA01000001.1
Yersinia mollaretii ATCC 43969	NZ_AALD02000001.1

Yersinia pestis biovar Antiqua B42003004	NZ_AAYU01000001.1
Yersinia pestis biovar Antiqua E1979001	NZ_AAYV01000001.1
Yersinia pestis biovar Antiqua UG05 0454	NZ_AAYR01000001.1
Yersinia pestis biovar Mediaevalis K1973002	NZ_AAYT01000001.1
Yersinia pestis biovar Orientalis F1991016	NZ_ABAT01000001.1
Yersinia pestis biovar Orientalis India 195	NZ_ACNR01000001.1
Yersinia pestis biovar Orientalis IP275	NZ_AAOS02000001.1
Yersinia pestis biovar Orientalis MG05 1020	NZ_AAYS01000001.1
Yersinia pestis biovar Orientalis PEXU2	NZ_ACNS01000001.1
Yersinia pestis CA88 4125	NZ_ABCD01000001.1
Yersinia pestis FV 1	NZ_AAUB01000001.1
Yersinia pestis KIM D27	NZ_ADDC01000001.1
Yersinia pestis Nepal516	NZ_ACNQ01000001.1
Yersinia pestis Pestoides A	NZ_ACNT01000001.1
Yersinia rohdei ATCC 43380	NZ_ACCD01000001.1
Yersinia ruckeri ATCC 29473	NZ_ACCC01000001.1
Yokenella regensburgei ATCC 43003	NZ_JH417859.1
