# Supplementary material for: Ray Meta: scalable de novo metagenome assembly and profiling
Source: Genome Biol. 2012 Dec 22;13(12):R122. doi: 10.1186/gb-2012-13-12-r122 (PMC4056372; doi:10.1186/gb-2012-13-12-r122)
Supplement: Additional file 3 — Documentation and scripts to reproduce all experiments. [file gb-2012-13-12-r122-S3.BZ2 › Paper-Replication-2012/Software-Validation-on-Mock-Community-Reads/table.pdf]

Table 1: Validation of Ray Communities using NIH Human Microbiome Project mock communities.
The mock communities were described in PAPER and the repository and accession numbers are available therein.

Species	16S copies per genome	Genome length (Mb)	Even mixture				Staggered mixture			
			Expected		Actual nucleotide proportion		Expected		Actual nucle[illegible] proportio[illegible]	
			16S copies	nucleotide proportion	Illumina	454	16S copies copies	nucleotide proportion	Illumina	45[illegible]
Acinetobacter baumannii (ATCC 17978)	5	4.02	100000	5.27%	12.68%	12.60%	10000	0.26 %	0.14%	0.[illegible]
Actinomyces odontolyticus (ATCC 17982)	3	2.39	100000	5.23%	2.80%	3.23%	1000	0.02 %	0.00%	0.[illegible]
Bacillus cereus (ATCC 10987)	12	5.43	100000	2.97%	0.17%	0.29%	100000	1.49 %	0.02%	0.[illegible]
Bacteroides vulgatus (ATCC 8482)	7	5.16	100000	4.84%	2.40%	2.14%	1000	0.02 %	0.00%	0.[illegible]
Candida albicans (SC5314)	0	27.56	1120	0.00%	0.00%	0.00%	1000	0.00 %	0.00%	0.[illegible]
Clostridium beijerinckii (ATCC 51743)	14	6.00	100000	2.81%	4.73%	4.00%	100000	1.41 %	0.90%	1.[illegible]
Deinococcus radiodurans (DSM 20539)	3	3.28	100000	7.17%	50.06%	51.95%	1000	0.03 %	0.08%	0.[illegible]
Enterococcus faecalis (ATCC 47077)	4	3.36	100000	5.51%	1.44%	1.59%	1000	0.02 %	0.00%	0.[illegible]
Escherichia coli (ATCC 70096)	7	4.60	100000	4.31%	0.06%	0.09%	1000000	21.73 %	0.35%	0.[illegible]
Helicobacter pylori (ATCC 700392)	2	1.66	100000	5.45%	3.56%	3.40%	10000	0.27 %	0.02%	0.[illegible]
Lactobacillus gasseri (DSM 20243)	6	1.89	100000	2.06%	0.00%	0.02%	10000	0.10 %	0.00%	0.[illegible]
Listeria monocytogenes (ATCC BAA-679)	6	2.94	100000	3.21%	1.77%	1.99%	10000	0.16 %	0.00%	0.[illegible]
Methanobrevibacter smithii (ATCC 35061)	2	1.85	100000	6.07%	0.73%	0.69%	1000000	30.59 %	5.52%	5.[illegible]
Neisseria meningitidis (ATCC BAA-335)	4	2.27	100000	3.72%	1.00%	1.21%	10000	0.18 %	0.00%	0.[illegible]
Propionibacterium acnes (DSM 16379)	3	2.56	100000	5.60%	0.01%	0.02%	10000	0.28 %	0.00%	0.[illegible]
Pseudomonas aeruginosa (ATCC 47085)	4	6.26	100000	10.27%	0.07%	0.15%	100000	5.17 %	0.42%	0.[illegible]
Rhodobacter sphaeroides (ATCC 17023)	3	4.60	100000	10.06%	3.43%	2.96%	100000	5.07 %	27.23%	21[illegible]
Staphylococcus aureus (ATCC BAA-1718)	5	2.94	100000	3.86%	4.24%	3.64%	1000000	19.45 %	29.25%	30[illegible]
Staphylococcus epidermidis (ATCC 12228)	5	2.49	100000	3.27%	6.22%	5.58%	100000	1.64 %	24.86%	27[illegible]
Streptococcus agalactiae (ATCC BAA-611)	7	2.16	100000	2.02%	0.01%	0.06%	1000000	10.20 %	0.32%	0.[illegible]
Streptococcus mutans (ATCC 700610)	5	2.03	100000	2.66%	2.34%	2.10%	1000	0.01 %	10.48%	11[illegible]
Streptococcus pneumoniae (ATCC BAA-334)	4	2.16	100000	3.54%	1.99%	1.60%	100000	1.78 %	0.00%	0.[illegible]
