# Supplementary figures and images for: Ray Meta: scalable de novo metagenome assembly and profiling
Source: Genome Biol. 2012 Dec 22;13(12):R122. doi: 10.1186/gb-2012-13-12-r122 (PMC4056372; doi:10.1186/gb-2012-13-12-r122)

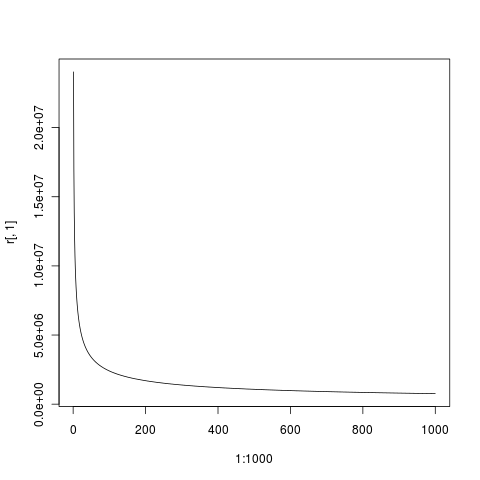

Supplement: Additional file 3 — Documentation and scripts to reproduce all experiments. [file gb-2012-13-12-r122-S3.BZ2 › Paper-Replication-2012/Simulation-1000-genomes/Sequencing/1000-bacteria-blueprint/plot.png]
